# Study of Retinoic Acid-Induced Osteoarthritis: Integrating RNA-Sequencing, Network Pharmacology, Molecular Docking, and Experimental Validation

**DOI:** 10.3390/ijms26125519

**Published:** 2025-06-09

**Authors:** Tao Lu, Zi-Yi Liu, Yang-Shuo Ge, Shuai-Yu Jiang, Qing-Ao Zhao, Dao-Fang Ding

**Affiliations:** 1 School of Traditional Chinese Medicine, Shanxi Datong University, Datong 037009, China; 03220010@sxdtdx.edu.cn (T.L.); jiangsy@sxdtdx.edu.cn (S.-Y.J.); 2 Datong Key Laboratory of Smart Medicine and Health Care for Elderly Chronic Diseases, Shanxi Datong University, Datong 037009, China; 221002011619@sxdtdx.edu.cn; 3 Institute of Rehabilitation Science, Shanghai University of Traditional Chinese Medicine, Shanghai 201203, China; 22023443@shutcm.edu.cn

**Keywords:** osteoarthritis, retinoic acid, chondrocytes, RNA-sequencing, network pharmacology, molecular docking, experimental validation

## Abstract

Osteoarthritis (OA) is a debilitating joint disorder characterized by cartilage degradation and disruption of chondrocyte homeostasis. Although retinoic acid (RA) has been used in OA models, its precise targets are not clear. A translational framework was employed, integrating RNA-sequencing results, network pharmacology prediction, computational ligand-receptor molecular docking, and biological experimental validation, to systematically elucidate RA’s disease-modifying targets in OA pathogenesis. RNA-sequencing of RA-treated chondrocytes revealed 656 differentially expressed genes (DEGs). Protein–protein interaction (PPI) network analysis and functional enrichment [Gene Ontology (GO)/Kyoto Encyclopedia of Genes and Genomes (KEGG)] highlighted key pathways, including extracellular matrix (ECM) reorganization and PI3K-Akt-mediated mechanotransduction and others. Network pharmacology analysis identified 42 shared targets between RA and OA. PPI analysis and functional enrichment (GO/KEGG) highlighted pathways including the renin–angiotensin system and the neuroactive ligand–receptor interaction, among others. Molecular docking ranked candidate targets by binding affinity of RA in descending order as MAPK14 (p38α), PTGER3 (PGE2 receptor), CA2 (CA2), and others. Five intersecting targets CA2, ACE, PTGS1 (COX-1), PGR, and EDNRA (ETAR) were identified by integrating RNA-sequencing (RNA-seq) results and network pharmacology predictions. These interactions were experimentally validated via western blot, RT-qPCR and immunofluorescence. RA increased the expression of MMP13, CA2 and ACE, and decreased the expression of COL2A1 in chondrocytes. siRNA-mediated knockdown of both CA2 (human CA2 homolog) and ACE (human ACE homolog) inhibit cartilage degradation through downregulating MMP13 and upregulating COL2A1. This study not only elucidates potential molecular mechanisms by which RA modulates chondrocyte catabolism but also offers a valuable reference for the development of novel OA therapeutics.

## 1. Introduction

OA affects approximately 7% of the global population [1] and more than 500 million people worldwide [2]. Clinically, knee OA is the most common form of arthritis, followed by hand and hip [3]. The development of OA is mainly associated with age [4], obesity [5], joint damage, inflammatory factors, and abnormal mechanical stresses within the joints [6]. With the global increase in elderly and obese populations, the incidence of OA is expected to rise, posing a profound economic and social burden [7,8]. It is worth noting that although OA is not directly life-threatening, it can cause disability, profoundly affecting patients’ emotional well-being and quality of life.

**Figure 1:**
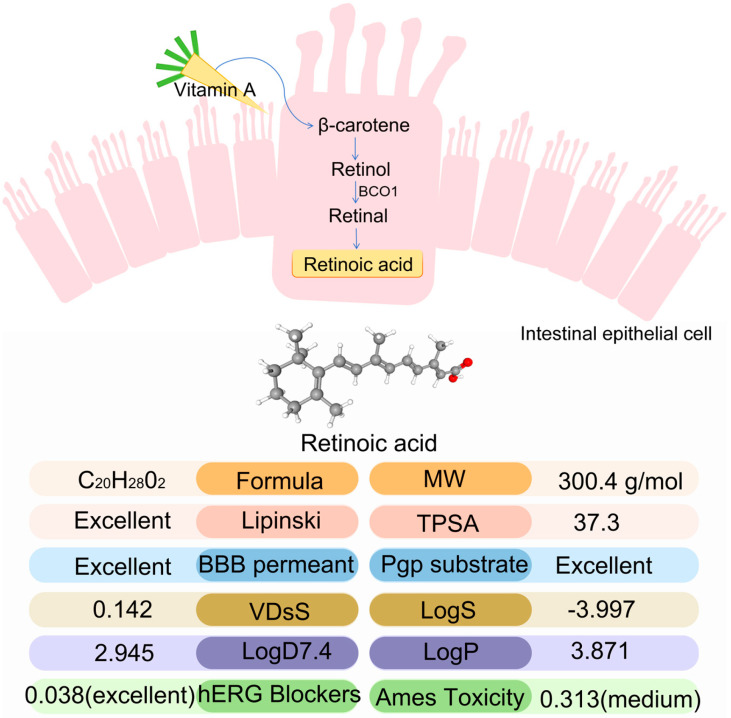
Information about RA metabolism in intestinal epithelial cells and its associated ADMET (absorption, distribution, metabolism, excretion, toxicity). Note: information on ADMET was taken from the ADMETlab 3.0 [https://admetlab3.scbdd.com (accessed on 29 January 2025)] database [20].

RA is a biologically active metabolite of vitamin A, playing a critical role in cell proliferation, differentiation, and immune responses [9]. Basic information about RA and its metabolism in vivo is illustrated in Figure 1. RA exhibits favorable pharmacokinetic properties, including good blood–brain barrier (BBB) permeability [10]. In addition, RA has been extensively studied in anti-tumor research and dermatological therapeutic studies, especially in the treatment of acute promyelocytic leukaemia (APL) [11,12]. RA has demonstrated efficacy in the treatment of dermatologic diseases; however, its clinical application is limited by notable side effects. Studies have shown that all-trans RA (ATRA) can inhibit chondrogenesis, and its toxicity can cause limb deformities [13,14]. Additionally, chronic exposure may lead to bone abnormalities in both children and adults, highlighting concerns about its long-term skeletal toxicity [15]. The study has shown bone hyperplasia to be a side effect of RA overdose, and similar changes have been observed in patients treated with isotretinoin for basal cell nevus syndrome [16]. The progression of OA is closely related to cartilage cell metabolic imbalance and ECM homeostasis disruption [17]. Pro-inflammatory cytokines such as IL-1β and TNF-α drive this imbalance by inducing matrix metalloproteinases (MMPs), particularly *MMP13*, leading to accelerated cartilage degradation [18,19]. Given this, OA-related catabolic activity can be evaluated by monitoring the expression of key genes, and RA may contribute to OA pathogenesis by modulating these catabolic pathways and gene expression profiles.

RA has been shown to influence chondrocyte catabolism through multiple mechanisms, including modulation of the cytoskeletal structure, enhancement of cell adhesion and spreading, regulation of ECM synthesis, and alteration of protein glycosylation patterns [21]. Together, these effects result in significant changes to the chondrocyte phenotype and function. Zengli Yu et al. found that RA suppressed chondrocyte metabolism and differentiation by interfering with the TGF-β3/TβRII/Smad signaling pathway, thereby impairing cartilage formation [22]. These findings were consistent with those reported previously by Maurizio Pacifici et al. [23]. However, the study has shown that inhibition of RA synthesis blocks limb bud formation, and exogenous RA supplementation can rescue this developmental defect [24]. Conversely, Kawata and colleagues successfully induced chondrogenic differentiation of pluripotent stem cells [25], demonstrating that RA synergizes with the Wnt/β-catenin signaling pathway to promote chondrocyte differentiation by regulating enhancers of chondrogenic transcription factors *SOX9*, *SOX5*, and *SOX6*, upregulating key genes like *SOX9* [26].

**Figure 2:**
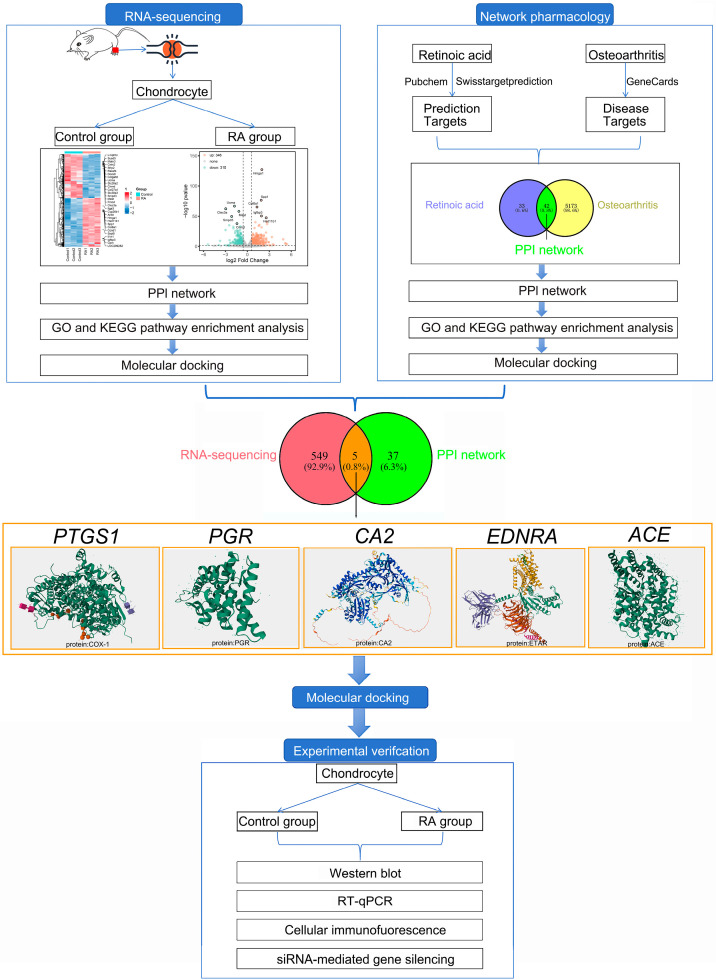
Flowchart of this study.

Given the complex and contradictory roles of RA in chondrocyte catabolism, further investigation is essential to clarify its precise effects in the context of OA. Our study aims to comprehensively evaluate the impact of RA on chondrocyte catabolism and identify the key genes and metabolic pathways involved in this process. The flow chart of this study is shown in Figure 2. Additionally, rat primary chondrocytes have been used in this experiment. Human gene symbols are applied to bioinformatics analyses.

## 2. Results

### 2.1. RNA-Seq Analyses Results

#### 2.1.1. Visualization of RNA-Seq DEGs Results by Heatmap and Volcano Plot

**Figure 3:**
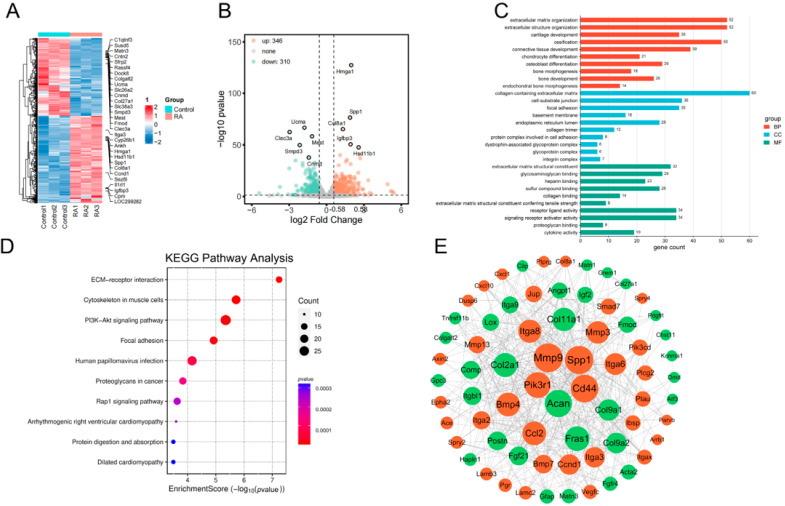
Identification and functional enrichment analysis of DEGs of RNA-Seq results. (**A**) Heatmap analysis of 656 DEGs between the control (CON) and RA groups, with expression levels ranging from low (blue) to high (red), was performed. (**B**) Volcano plot analysis of 656 DEGs between the CON and RA groups. (The upper left and upper right portions of the dashed line indicate valid differential genes i.e., *p*-value < 0.05 & log_2_FoldChange| > 0.58.) (**C**) GO analysis of enrichment between the CON and RA groups included biological process (BP), cellular component (CC), and molecular function (MF). (**D**) KEGG enrichment analysis between the CON and RA groups. (**E**) The original PPI network of therapeutic targets, where red is up-regulated genes, and green is down-regulated genes.

Using a false discovery rate (FDR) threshold of <0.05, 656 DEGs were identified through the RNA-Seq analysis. A heatmap and volcano plot were generated to visualize the transcriptional changes induced by RA in chondrocytes (Figure 3A,B); these analyses revealed three major RA-driven pathogenic axes: (1) cartilage catabolism, marked by upregulation of *MMP13* (+2.3-fold) along with suppression of *ACAN* (−1.5-fold) and *COL2A1* (−0.8-fold), coupled with activation of NF-κB-mediated inflammasome; (2) metabolic reprogramming, marked by glycolytic shift (*CA2* +1.2-fold) and lipid metabolism dysregulation (*PLA2G4A* +0.3-fold); and (3) structural reorganization, characterized by ECM destabilization (*COL27A1* −1.4-fold, *HAPLN1* −1.3-fold) and integrin signaling activation (*ITGA5* +1.9-fold).

#### 2.1.2. PPI Network of RNA-Seq DEGs

To capture the most functionally relevant interactions, we constructed a PPI network by selecting DEGs with a node degree greater than10, ensuring both focus and comprehensiveness. Central nodes within the network included *MMP9* (degree = 39), *SPP1* (degree = 38), *ACAN* (degree = 38), *CD44* (degree = 37), and *PIK3R1* (degree = 34), underscoring their pivotal roles in OA. *MMP9* and *SPP1* are known to cooperate in ECM remodeling and cell migration, while *CD44* and *PIK3R1* participate in signal transduction processes that govern cell adhesion and survival. *ACAN*, a cartilage-specific proteoglycan, serves as a crucial mediator linking matrix integrity to overall tissue homeostasis. The network was visualized using Cytoscape 3.10.0, with node sizes scaled according to degree values, as depicted in Figure 3E. This integrative analysis underscored the centrality of these hub genes in OA pathogenesis and their potential as therapeutic targets.

#### 2.1.3. GO and KEGG Pathway Analysis of RNA-Sequencing DEGs

GO enrichment analysis was performed for DEGs (Figure 3C,D). The most significantly enriched BP terms were associated with extracellular matrix organization, extracellular structure organization, cartilage development, etc. CC terms included collagen-containing extracellular matrix, cell-substrate junction, focal adhesion, etc. For MF, the most enriched terms encompassed extracellular matrix structural constituents, glycosaminoglycan binding, heparin binding, etc. Further KEGG pathway analysis revealed that the DEGs are significantly involved in key signaling pathways, including ECM-receptor interaction (corrected *p*-value = 2.89 × 10^−5^), the PI3K-Akt signaling pathway (*p* = 0.000146), and focal adhesion (*p* = 0.000146), which are critical for cell-ECM communication, cell survival, and mechanotransduction.

#### 2.1.4. Molecular Docking Validation Between RA and the Top 50 Genes Exhibiting the Largest Differential Expression in RNA-Seq DEGs

Table 1 presents the molecular docking results between RA and the top 50 genes with the most significant differential expression identified in RNA-Seq analysis. The table details each gene symbol, its corresponding protein name, and the total docking score, which indicates the predicted binding affinity between RA and the encoded protein. Docking scores range from 0.83 (*MGLL*) to 7.64 (*ACAN*), reflecting a broad spectrum of interaction strengths. Notably, *ACAN* exhibits the highest score (7.64), suggesting a strong binding affinity. Other high-scoring candidates include *KANSL2* (6.97), *SFRP2* (6.96), *CNTN2* (6.44), and *COL27A1* (6.04), all of which may serve as key molecular targets for further investigation. Moderate to high docking scores are observed for several other genes, such as *INHBE* (5.74), *POSTN* (5.73), *HAPLN1* (5.84), and *RPL35A* (5.65), which may also participate in RA-mediated chondrocyte catabolism. Conversely, genes like *MGLL* (0.83) and *ENHO* (2.01) show weak interactions, suggesting limited direct involvement in RA-binding affinity.

### 2.2. Network Pharmacology Analysis Results

#### 2.2.1. Identification of Shared Targets Between RA and OA

**Figure 4:**
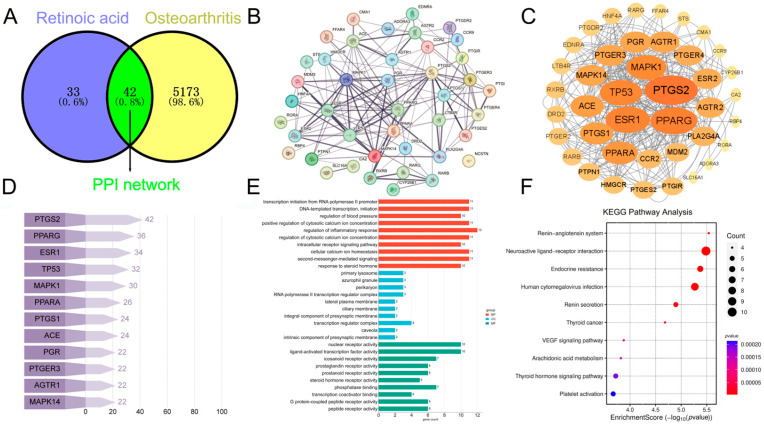
Network pharmacology analysis results: (**A**) Intersection targets of RA and OA. (**B**) PPI network constructed from the STRING. (**C**) PPI network constructed from Cytoscape 3.10.0. (**D**) Horizontal stereo sensory bar graphs of the first 12 genes in the degree. (**E**) GO enrichment analysis of RA-induced OA. (**F**) KEGG enrichment analysis of RA-induced OA.

A total of 100 potential targets of RA were retrieved from the SwissTarget prediction database. After filtering for targets with a prediction probability greater than 0, 75 candidate targets were retained. Separately, a search of the Genecards database using OA as a keyword yielded 5215 OA-related targets. These two datasets were cross-referenced using the Venny platform, identifying 42 overlapping targets shared between RA and OA, as illustrated in Figure 4A.

#### 2.2.2. Analyzing Core Molecular Targets Using PPI Network Analysis

The intersecting targets were subjected to PPI analysis (Figure 4B). The PPI network consisted of 42 nodes and 159 edges, with an average node degree of 7.57 and an average local clustering coefficient of 0.593. The PPI enrichment *p*-value was <1.0 × 10^−16^, indicating significant interaction among these proteins. The interaction data were imported into Cytoscape 3.10.0 for visualization (Figure 4C), where node size reflects connectivity (degree). Twelve key hub proteins with the highest degrees were identified (Figure 4D) (*PTGS2*:42, *PPARG*:36, *ESR1*:34, *TP53*:32, *MAPK1*:30, *PPARA*:26, *PTGS1*:24, *ACE*:24, *PGR*:22, *PTGER3*:22, *AGTR1*:22, *MAPK14*:22). Among these, *PTGS2* (42), *PPARG* (36), *ESR1* (34), *TP53* (32), and *MAPK1* (30) displayed the highest connectivity, underscoring their potential as core regulatory nodes in the RA–OA interaction network.

#### 2.2.3. GO and KEGG Pathway Analysis of DEGs in Chondrocytes Treated with RA

The 42 target genes were analyzed using the DAVID platform, and entries with *p*-value < 0.05 were selected from the GO functional enrichment analysis results. GO enrichment analysis revealed that the intersecting genes were associated with 214 MF terms, predominantly including nuclear receptor activity, ligand-activated transcription factor activity, eicosanoid receptor activity, prostaglandin receptor activity, prostanoid receptor activity, and steroid hormone receptor activity. Additionally, the intersecting genes were enriched in 2330 BP terms, primarily involved in transcription initiation from RNA polymerase II promoter, DNA-templated transcription, positive regulation of cytosolic calcium ion concentration, regulation of inflammatory response, and modulation of cytosolic calcium ion concentration. Furthermore, 119 CC terms were enriched, including primary lysosome, azurophil granule, perikaryon, RNA polymerase II transcription regulator complex, lateral plasma membrane, and ciliary membrane (Figure 4E). The KEGG pathway enrichment analysis, visualized as a bubble map (Figure 4F), identified 184 pathways, with the top 10 signaling pathways being the renin-angiotensin system, neuroactive ligand-receptor interaction, endocrine resistance, human cytomegalovirus infection, renin secretion, thyroid cancer, VEGF signaling pathway, arachidonic acid metabolism, thyroid hormone signaling pathway, and platelet activation. These findings highlight the involvement of the target genes in critical signaling pathways and BP, providing mechanistic insights into the role of RA in OA pathogenesis.

#### 2.2.4. Molecular Docking Validation Between RA and 42 RA–OA Shared Targets Identified via Network Pharmacology Analysis

The molecular docking results for 42 intersecting targets shared between RA-associated drug targets and OA-related disease targets are presented in Table 2. Docking scores range from 4.33 (*RORA*) to 8.01 (*MAPK14*), indicating a broad spectrum of binding affinities. Notably, *MAPK14* exhibits the highest docking score (8.01), suggesting a strong interaction with RA, which may highlight its central role in the molecular mechanisms. Other high-affinity targets include *MAPK1* (6.93), *AGTR2* (6.93), *CCR2* (6.91), and *CA2* (7.44), all of which may serve as key therapeutic targets. In addition, several targets, such as *PTGER3* (EP3R, 6.64), *PTGS2* (COX-2, 6.14), and *PPARG* (6.50), also show strong binding, underscoring their potential relevance in disease modulation.

Nuclear receptors and transcriptional regulators, including *HNF4A* (6.28), *PPARA* (6.29), and *ESR1* (ERα, 6.29), further emphasize the potential effects of RA on gene expression networks. While targets like *RORA* (4.33), *ADORA3* (5.56), and *DRD2* (4.53) show moderate to lower docking scores, they may contribute to the broader biological effects of RA.

### 2.3. Molecular Docking Validation Between RA and the Intersecting Targets Identified by RNA-Seq and Network Pharmacology

**Figure 5:**
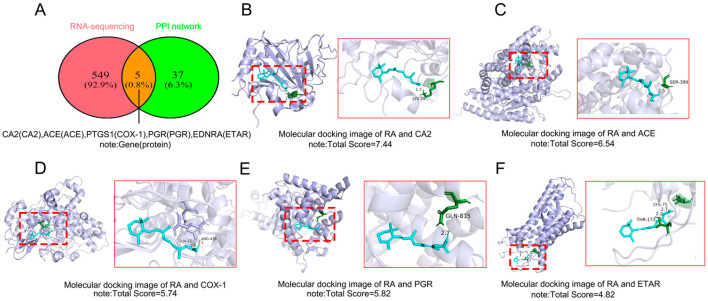
Molecular docking results: (**A**) The five intersecting target proteins were obtained from RNA-Seq and network pharmacology. (**B**–**F**) Results of molecular docking analysis between RA and CA2 (*CA2*, 7.44), ACE (*ACE*, 6.54), PGR (*PGR*, 5.82), COX-1 (*PTGS1*, 5.74), ETAR (*EDNRA*, 4.82). (The dashed box indicates the position of the right enlargement in the original docking plan.)

The target proteins identified through RNA-Seq analysis and network pharmacology were subjected to molecular docking with RA (Figure 5). Five target proteins, including *CA2*, *ACE*, *PGR*, *PTGS1*, and *EDNRA,* were identified for docking analysis (Figure 5A). Molecular docking using SYBYL-X 2.0 revealed distinct binding affinities between RA and these targets, as reflected by total scores (Figure 5B–F), with higher scores indicating stronger interactions. The ranking of binding affinities was as follows: *CA2* (7.44) > *ACE* (6.54) > *PGR* (5.82) > *PTGS1* (5.74) > *EDNRA* (4.82). Notably, *CA2* and *ACE* exhibited significantly higher docking scores than the other proteins, suggesting a stronger binding capacity. This may be attributed to their nature as zinc ion-dependent metalloenzymes, which possess active sites that are highly complementary to RA in terms of electrostatic and hydrophobic interactions. Furthermore, a high docking score generally reflects favorable physicochemical compatibility, including electrostatic complementarity and hydrophobic interaction potential. Proteins with high binding energies may play a crucial role in RA-induced OA pathogenesis and represent promising therapeutic targets that warrant further investigation. In contrast, *EDNRA*, *PTGS1*, and *PGR* displayed relatively lower scores, potentially due to the absence of metal ion coordination or suboptimal binding site architecture.

### 2.4. Experimental Validation

#### 2.4.1. Analyzing the Expression of Representative DEGs and Target Genes Identified by RNA Sequencing and Network Pharmacology

**Figure 6:**
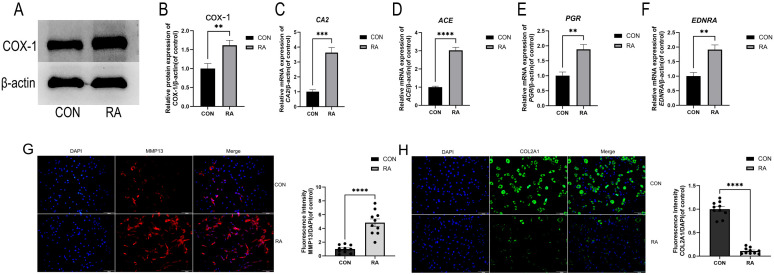
Experimental validation results: (**A**,**B**) *PTGS1* protein levels were assessed by western blotting in the indicated groups. (**C**–**F**) Relative mRNA expression levels of *CA2*/, *ACE*/β-actin, *PGR*/β-actin and *EDNRA*/β-actin were measured. Data are means ± SD. n = 3, ** *p* < 0.01, *** *p* < 0.001, **** *p* < 0.0001. (**G**) *MMP13* expression (red) was used to assess OA after a 24-h culture period, with DAPI (blue) used for nuclear counterstaining. (**H**) *COL2A1* expression (red) was used to assess the formation and maintenance of cartilage after a 24 h culture period, with DAPI (blue) used for nuclear counterstaining. Scale bar: 100 µm.

Western blotting and RT-qPCR results (Figure 6A–F) showed that the expression levels of COX-1 (*PTGS1*), CA2 (*CA2*), ACE (*ACE*), PGR (*PGR*), and ETAR (*EDNRA*) were significantly higher in the RA group than those in the CON group, suggesting that these molecules may play important roles in the pathological process of OA. Compared to the CON group, the expression level of *MMP13* was significantly upregulated in the RA group (Figure 6G), whereas *COL2A1* expression was notably downregulated (Figure 6H).

#### 2.4.2. Validation of Silencing Target Gene

**Figure 7:**
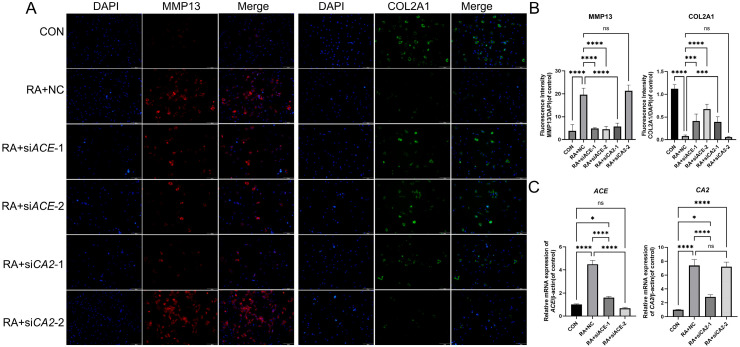
Determine the functions of *ACE* and *CA2* in RA-treated chondrocytes: (**A**) Immunofluorescence staining of MMP13 (red), COL2A1 (green), and DAPI (blue) from different treatment groups. Scale bar: 100 µm. (**B**) Quantification of fluorescence density of MMP13 and COL2A1 expression levels in different groups. (**C**) Determine the silencing efficiency of *ACE* and *CA2* by RT-qPCR. * *p* < 0.05, *** *p* < 0.001, **** *p* < 0.0001, ns means not significant.

To verify the promoted catabolism of RA through the upregulation of *ACE*/*CA2* in chondrocytes, siRNA-mediated gene silencing of *ACE* and *CA2* in chondrocytes was investigated. RA treatment significantly increased *MMP13* expression and decreased *COL2A1* expression compared to the CON group (*p* < 0.05), indicating enhanced chondrocyte catabolism. Co-treatment with RA and si*ACE-1* or si*ACE-2* or si*CA2-1* efficiently reduced *MMP13* and increased *COL2A1* (*p* < 0.001 vs. RA group), confirming both *ACE* and *CA2* as catalytic-promoting genes in RA-treated chondrocytes (Figure 7A,B). PCR results further confirmed that the expression levels of ACE and CA2 were significantly reduced after silencing ACE with siACE-1 or siACE-2, and CA2 with si*CA2*-1 (Figure 7C).

## 3. Discussion

This study systematically deciphers the molecular interplay between RA and OA through an integrative approach, including RNA-Seq, network pharmacology, molecular docking, and experimental validation. Five target proteins were screened via these methods, the expression of which was significantly increased in RA-treated chondrocytes, suggesting their central role in chondrocyte catabolism. In particular, zinc-dependent metalloenzymes (*CA2* and *ACE*) emerged as pivotal therapeutic targets through gene silencing experiments. In the following sections, we contextualize these results, explore their broader implications, and propose directions for future research.

The RNA-Seq-derived DEGs and functional enrichment analyses highlight ECM disassembly as a crucial event in RA-induced OA. Key ECM components, including *ACAN* (aggrecan) and *COL27A1*, were markedly dysregulated, aligning with prior studies linking ECM degradation to cartilage loss in OA [27,28]. In line with this, COL2A1 was significantly downregulated, while MMP13 was upregulated in RA-treated chondrocytes, echoing clinical OA phenotypes marked by collagen fragmentation and proteoglycan loss [29,30]. Notably, molecular docking identified *ACAN* as a high-affinity target (Total Score 7.64), suggesting that RA may directly impair aggrecan stability. Collectively, these findings underscore the importance of ECM preservation in therapeutic strategies, with small molecules targeting aggrecan–collagen interactions, which offer promising potential for cartilage regeneration.

Network pharmacology identified 42 shared RA-OA targets, with *PTGS2*, *PPARG*, *ESR1*, *TP53*, and *MAPK1* emerging as key nodes in the PPI network. The high docking scores of *MAPK14* (8.01) and *ACE* (6.54) highlight their structural compatibility with RA, likely mediated by zinc-coordinated active sites. *MAPK14*, a central regulator of pro-inflammatory cytokines (e.g., TNF-α, IL-1β), aligns with its established role in synovitis and cartilage catabolism [31]. Similarly, *ACE*, a key player in the renin–angiotensin system (RAS), has roles extending beyond vascular regulation. Emerging evidence links RAS activation to OA, where angiotensin II drives oxidative stress and fibrosis [32]. Our data suggest that RA exacerbates OA by hijacking these pathways, thereby amplifying inflammation and ECM degradation.

RNA-Seq analysis of chondrocytes identified key genes and signaling pathways regulated by RA, elucidating its role in cartilage degeneration. Network pharmacology revealed RA’s involvement in OA. By integrating RNA-Seq and network pharmacology datasets, the intersection analysis provided deeper mechanistic insights and pinpointed potential therapeutic targets, which were subsequently validated through molecular docking with RA. This integrated approach strengthens the reliability of the findings and provides a comprehensive understanding of RA’s molecular mechanisms in OA. Molecular docking validation between RA and the shared targets identified from RNA-Seq and network pharmacology suggests that *CA2*, a zinc-dependent metalloenzyme with the highest docking score (7.44), emerged as a novel OA mediator in OA. While *CA2* is classically associated with pH regulation [33], its overexpression in RA-induced OA implies a role in acidifying the cartilage microenvironment. This mechanism parallels ECM breakdown in cancer metastasis [34], indicating a potentially conserved pathological pathway that could be targeted with *CA2* inhibitors (e.g., acetazolamide). *ACE* not only modulates vascular tone but also interacts with *MAPK14* via angiotensin II-mediated NF-κB activation [35], creating a feed-forward loop of inflammation and ECM degradation.

Cuijiang Wang et al. found [36] that *PTGS1* was highly expressed in the synovial tissues of OA patients, with significantly increased levels compared to normal tissues [36]. In our study, *PTGS1* expression was also increased in RA-stimulated chondrocytes, supporting its role in inflammation-driven cartilage damage. *MMP13* is a key enzyme involved in the degradation of the cartilage matrix. Interestingly, *PGR* inhibition resulted in increased *MMP13* levels, suggesting that progesterone may exert a chondroprotective effect by downregulating *MMP13* through *PGR*-mediated signaling [37]. These findings imply that during the early stages of OA, *PGR* expression is upregulated as a compensatory mechanism to promote bone formation and maintain joint stability.

The elevated expression of *CA2* in OA may help to explain why SM/J mice are resistant to ectopic calcification [38]. The *EDNRA* gene encodes endothelin receptor A, which is expressed in multiple cell types and is involved in endothelin-1 signaling. Endothelin-1 has vasoconstrictive, pro-fibrotic, and pro-inflammatory properties, which may indirectly influence pain perception. Particularly in the context of OA, endothelin-1 may exacerbate joint pain through its pro-inflammatory effects [39]. Moreover, the study has shown that the *EDNRA* gene is associated with the transforming growth factor alpha (TGFα) signaling pathway in OA, where TGFα induces upregulation of *EDNRA* expression at both the mRNA and protein levels [40]. In our study, *PCR* results revealed increased *EDNRA* gene expression in the RA group, further supporting the hypothesis that RA may trigger OA-related changes. Genjun Chen et al. revealed an association between the I/D polymorphism (insertion/deletion polymorphism) of the Angiotensin-Converting Enzyme (ACE) gene and the risk of knee OA in the Chinese Han population, with the correlation being more pronounced in certain subgroups [41].

This study utilized RNA interference (RNAi) technology to systematically elucidate the regulatory functions and mechanisms of *ACE* and *CA2* in cartilage metabolism. It has confirmed that *ACE*, acting as a key upstream factor, mediated RA-induced upregulation of *MMP13* expression (*p* < 0.001), and its silencing significantly inhibited collagen degradation, which suggests that targeting *ACE* may block the process of cartilage degeneration in OA. Subsequent meta-analyses confirmed this polymorphism as a genetic risk factor for OA, especially in Asian and Caucasian populations, aligning with our observation of increased *ACE* expression in RA-treated chondrocytes [42]. Similarly, *CA2* silencing induced *the expression of COL2A1 protein and mRNA*. These findings reveal that RA disrupts cartilage homeostasis through a ‘double whammy’ mechanism (promotion of collagen degradation by *MMP13*/inhibition of *COL2A1* synthesis).

While this study elucidates the molecular mechanism underlying RA-induced OA, several limitations should be taken into consideration. First, the reliance on in vitro systems and computational predictions necessitates the further validation of in vivo OA models and human clinical cohorts. Second, the functional significance of lower-scoring targets (e.g., *EDNRA*) remains unclear; whether they contribute subtly to OA or represent false positives requires deeper investigation. Third, the temporal dynamics of target gene expression across OA stages were not assessed; longitudinal studies could reveal stage-specific vulnerabilities.

In summary, RA-induced OA appears to be driven by ECM degradation, with *CA2* and *ACE* serving as key targets of metalloenzymes. By integrating multi-omics and structural docking, this work unravels disease mechanisms and charts a course for rational drug design. Future efforts to translate these insights into therapies could disrupt OA progression, offering hope for a condition currently lacking disease-modifying treatments. RA-induced OA animal models serve as indispensable tools for elucidating disease mechanisms and advancing therapeutic discovery. While our findings highlight the translational potential of *CA2/ACE*-targeted strategies, we emphasize the need for multidisciplinary efforts that integrate advanced omics, spatial transcriptomics, and humanized OA models to bridge the gap between preclinical findings and clinical application. We strongly encourage future studies to explore the dose-dependent effects of retinoid signaling in OA pathogenesis and validate combination therapies in longitudinal cohorts. Such endeavors will ultimately accelerate the development of precision medicine for patients with OA.

## 4. Materials and Methods

### 4.1. Chondrocyte Cell Culture

In our experiments, we chose 24-h-old specific pathogen-free (SPF) female Sprague-Dawley rats provided by Shanghai XipurBiak Laboratory Animal Co., Ltd. (located in Shanghai, China.) with the laboratory animal license number SCXK (Shanghai) 2023-0009 as experimental animals; the quality certificate number of this batch of animals is NO.20230009011756. Following the method described by Wang et al. [36], 0.1% type II collagenase (BS164, Biosharp, Beijing, China) was to digest the cartilage tissues at 37 °C for a continuous period of 2-3 h. Subsequently, chondrocytes were cultured in Dulbecco’s Modified Eagle’s Medium (DMEM, Hyclone, Logan, UT, USA) containing 10% fetal bovine serum (FB-1058, Biosera, Cholet, France) and 1% penicillin-streptomycin (ST488S, Beyotime, Shanghai, China). The chondrocytes used in this experiment were all at passage 1 or passage 2 after isolation. Before RNA-seq, the chondrocytes in the CON group were cultured in a high-glucose DMEM medium containing 10% FBS for 24 h, while cells in the RA group (RA) were cultured in the same medium with 1 μM RA for 24 h [28,43]. The RA isomer used in this experiment was ATRA, purchased from Selleckchem (Item No. NSC 122758, Houston, TX, USA).

### 4.2. RNA-Seq

In the RNA-seq, RNA was extracted from three independent samples for each group and sequenced using the Illumina HiSeq 4000 instrument by Shanghai Life Genes Technology Co., Ltd. (Shanghai, China). RNA quality was assessed by the RNA Nano 6000 Assay Kit and the Bioanalyzer 2100 instrument (Agilent Technologies, Santa Clara, CA, USA). Subsequently, sequencing libraries were constructed using a NEBNext^®^ Ultra™ RNA Library Prep Kit for Illumina^®^ (NEB, lpswich, MA, USA), and fragment purification was performed by the AMPure XP system (Beckman Coulter, Beverly, CA, USA) to ensure the selection of fragments of appropriate length and quality. DEGs were identified based on a *p*-value of less than 0.05, and these DEGs were functionally annotated using the GO database. DEGs were identified based on a *p*-value < 0.05 and |log_2_FC| > 0.58 (equivalent to 1.5-fold change). The fold change (FC) in gene expression between the RA and the CON was calculated using the formula: FC = Log_2_ (FPKM_RA/FPKM_CON). Where FPKM_RA represents the FPKM value of a gene in the RA group and FPKM_CON represents the FPKM value of the same gene in the CON group. DEGs were functionally annotated using the GO database. The RNA-seq data generated in this study is available in the GEO repository under accession GSE298569.

### 4.3. Network Pharmacology Analysis

RA was searched in the Pubchem [https://pubchem.ncbi.nlm.nih.gov/ (accessed 8 November 2024)] database [44] and its SMILES was CC1=C(C(CCC1)(C)C/C=C/C(=C/C=C/C(=C/C(=O)O)/C)/C. The SMILES was entered into the SwissTargetPrediction [http://swisstargetprediction.ch// (accessed on 8 November 2024)] database [45], which in turn predicted RA targets and screened targets with a probability greater than 0. Screening and obtaining OA-related genes as well as protein targets was achieved using the Genecards [https://www.genecards.org/ (accessed on 8 November 2024)] database [46]. This comprehensive screening strategy laid the foundation for subsequent network pharmacology analyses. The potential drug targets of RA and OA-related target genes were imported into Venny 2.1 [https://bioinfogp.cnb.csic.es/tools/venny/ (accessed on 14 November 2024)] online tool, and the two sets were intersected to create custom Venny diagrams.

### 4.4. Cross-Species Gene Symbol Switch

To bridge the species gap between rat RNA-seq data and human network pharmacology targets, we performed cross-species gene symbol conversion using the following approach: first, DEGs from rat chondrocytes (RA vs. CON) were identified using their original mixed-case nomenclature. Since human gene symbols follow uppercase conventions, we mapped rat genes to their human orthologs by submitting the full names of rat genes to the STRING database [https://cn.string-db.org/ (accessed on 15 November 2024)], which provided standardized human gene symbols based on conserved orthology. This conversion enabled a direct comparison between rat-derived DEGs and human drug-target genes from network pharmacology analysis, ensuring the accurate identification of the intersection for downstream pathway mapping. The orthology-based translation accounted for potential nomenclature discrepancies while maintaining biological relevance across species.

### 4.5. PPI Network Construction

The targets of the PPI network include (1) differential genes in RNA-Seq and (2) interaction-targeting of RA drugs and OA diseases in network pharmacology. Each of these targets was imported into the STRING database [47], and the analysis was restricted to humans for protein-protein interaction (PPI) analysis. The STRING database was accessed to construct a PPI network for classifying the cross-target genes obtained by Venny, and the network was visualized using Cytoscape 3.10.0 software. The Network Work Analyzer plug-in tool was used to construct a PPI network diameter map by analyzing the density values (degrees) of the nodes in the network.

### 4.6. GO and KEGG Pathway Enrichment Analysis

To systematically explore disease mechanisms and enhance network properties, we conducted separate functional enrichment analyses for both RNA-Seq and network pharmacology data. The intersecting target-associated genes were analyzed using the DAVID database [https://david.ncifcrf.gov (accessed on 15 November 2024)] [47] for Gene Ontology (GO) functional annotation and KEGG pathway enrichment. Significant terms (*p*-value < 0.05) were visualized as bar and bubble plots through the bio-information website [http://www.bioinformatics.com.cn/ (accessed on 15 November 2024)] [48,49]. This dual analytical approach enabled comprehensive pathway investigation and network expansion for both experimental modalities.

### 4.7. Molecular Docking

The following targets were selected for molecular docking with RA using SYBYL-X 2.0 to compare the binding activity of RA to different targets. (1) The most significant genes (DEGs, |log_2_FC| ≥ 1, *p* < 0.01) among the top 50 DEGs obtained by RNA-Seq, (2) intersecting targets of RA drug targets and OA disease targets obtained by network pharmacology, (3) the intersection of RNA-Seq and network pharmacology. The specific methods were as follows: (1) we downloaded the SDF file of 2D RA from the PubChem database, converted it to MOL2 format by Open Babel software [50], used it as a ligand, and searched the Uniprot [https://www.uniprot.org/ (accessed on 15 November 2024)] database [51] for the three parts of the targets mentioned above, and the structure was chosen to be the light wavelength and molecular diameter the smallest; the smaller the value, the higher the resolution and the more accurate the structure. The obtained PDB numbers were entered into the PDB [https://www.rcsb.org/ (accessed on 16 November 2024)] database [52] and downloaded in PDB format, and the above structures were entered into the SYBYL-X 2.0 software [53] for molecular docking PDB database to obtain the 3D structure, after which the ligand RA was extracted, the water of crystallization was removed, hydrogenation and charge were added, the protein structure was repaired to generate a docking pocket, and then the RA was docked to this pocket by selecting the high-precision docking mode (GeoM), ticking the Cscore option, and leaving the rest of the parameters as default values. (2) We imported the receptor and ligand models into SYBYL-X 2.0 for operation, and after docking was completed, among all the docking results with a C-score scoring not less than 4, the highest score of Total Score was selected as the final score, which indicated a strong binding between the receptor and ligand. Final image visualization was performed using PyMOL 3.1 software.

### 4.8. Western Blot

Lysis buffer (P0013B, Beyotime, Shanghai, China), containing phenylmethylsulfonyl fluoride (PMSF), was used to lyse the cells. The lysates were centrifuged at 12,000× *g* for 10 min at 4 °C, after which a Bicinchoninic Acid (BCA) kit (Cat. No. 23227, Pierce, Waltham, MA, USA) was used to measure protein concentrations. Samples were then separated on 10% sodium dodecyl sulfate-polyacrylamide gel electrophoresis (SDS-PAGE) and transferred to polyvinylidene fluoride (PVDF) membranes. Blots were probed overnight at 4 °C with the appropriate antibodies, followed by incubation for 1 h with anti-rabbit secondary antibodies (1:10,000, 7074S, CST, Danvers, MA, USA). Proteins were visualized using enhanced chemiluminescence (Pierce Biotechnology). The primary antibodies used were specific for β-actin (1:5000, AF2811, Beyotime, Shanghai, China), and *PTGS1*(COX-1) (1:2000, A5555, Bimake, Beijing, China).

### 4.9. siRNA Experiments

Four siRNAs that target *ACE* and *CA2* (*ACE-1, ACE-2, CA2-1, CA2-2*) were designed, and the sequences were as follows:

*ACE-1* (2041 bp): 5′-ACGCAGAGGCCAACTGGCATTATAA-3′ (ORF48.0%)

*ACE-2* (2017 bp): 5′-CAGCCAAGGTGTTGTGGAACGAATA-3′ (ORF48.0%)

*CA2-1* (750 bp): 5′-CAGCCGCTGAAGAACAGAAAGATCA-3′ (ORF48.0%)

*CA2-2* (141 bp): 5′-CAGCCTCTGCTCATATGTTACGATA-3′ (ORF44.0%)

Cells were washed twice with PBS and replaced with 500 μL of antibiotic-free medium for pretreatment. Prior to siRNA preparation, the samples were centrifuged at 10,000× *g* for 1 min. A 20 μM master mix (20 pmol/μL) was prepared by dissolving 1 nmol of each siRNA in 50 μL of nuclease-free water. For transfection, 1 μL of siRNA was diluted in 50 μL of antibiotic- and serum-free medium. Separately, 1 μL of Lipofectamine™ 2000 transfection reagent (2001808, Invitrogen, Thermo Fisher Scientific, Carlsbad, CA, USA) was diluted in 50 μL of antibiotic- and serum-free medium. These two solutions were incubated at room temperature for 5 min, then combined and incubated for an additional 20 min to allow for complex formation. The siRNA–lipofectamine mixture was then added to the antibiotic- and serum-free medium, gently mixed, and incubated at 37 °C with 5% CO_2_ for 4–6 h, followed by replacement with complete medium.

### 4.10. RT-qPCR

For reverse transcription using AG11706 reagent (Accurate Biology, Changsha, China), the volume required to reach 1000 ng of RNA was first calculated based on the RNA concentration. A 20 μL reaction system consisting of Reverse Transcription Specific Water, 5× Reverse Transcription Mix, and RNA was then prepared on ice. The reverse transcription reaction was then carried out using a SimpliAmp Thermal Cycler instrument (Thermo Fisher Scientific, Carlsbad, CA, USA), setting the temperature at 37 °C for 15 min, heating at 85 °C for 5 s, and then lowering to 4 °C to maintain. After completion of reverse transcription, the cDNA was diluted 10-fold, followed by PCR reaction using AG11718 reagent (Accurate Biology, Changsha, China). 2×SYBR Green Mix, primer mixture, reverse transcription-specific water, and diluted cDNA template were added to 20 μL of the PCR reaction system. *PCR* reaction conditions were set to 95 °C pre-denaturation for 30 s, followed by 40 cycles of 95 °C denaturation for 5 s and 60 °C annealing extension for 30 s. The *PCR* reaction conditions were set to 95 °C pre-denaturation for 30 s and 60 °C annealing extension for 30 s. The *PCR* reaction was performed at the same time. The primers used are listed in Table 3, and samples were analyzed in triplicate.

### 4.11. Cellular Immunofluorescence

Chondrocytes in 24-well plates were fixed for 10 min with 4% PFA, washed twice with PBS, permeabilized for 15 min with 0.1% Triton X-100 in PBS at room temperature (RT), blocked for 1 h with 10% BSA, and probed for 1 h at RT with anti-*COL2A1* (1:200, SC- 52658, Santa, Dallas, TX, USA) and *MMP13* (1:200, Sc-30073, Santa, Dallas, TX, USA) in 1% BSA. After three rinses with PBS, the cells were probed for 30 min with a secondary antibody (1:1000, A32723, Invitrogen, Carlsbad, CA, USA) in 1% BSA at room temperature, and nuclei were stained with 10 ng/mL DAPI (1:1000, 249-186-7, Applichem, Darmstadt, Germany).

### 4.12. Statistical Analysis

Data are expressed as mean ± standard deviation (SD) from three biologically independent replicates. Statistical comparisons were performed using Student’s *t*-test (two-group comparisons) or one-way ANOVA (multi-group comparisons) in GraphPad Prism 9.5.0 (GraphPad Software, San Diego, California, USA). A *p*-value < 0.05 was considered statistically significant.

## 5. Conclusions

Molecular docking of RA with the top 50 DEGs revealed strong binding affinities for *ACAN*, *SFRP2*, and *KANSL2*, underscoring their potential roles in RA-mediated OA pathogenesis. Network pharmacology identified 42 shared targets between RA and OA. Molecular docking of these shared targets prioritized *MAPK14*, *PTGER3*, and *CA2* as high-affinity interactors, suggesting their central roles in RA-induced OA. Further intersection analysis of RNA-Seq and network pharmacology data identified five key targets (*CA2*, *ACE*, *PTGS1*, *PGR*, and *EDNRA*). Experimental validation confirmed *CA2* and *ACE* as central hubs in RA-mediated OA pathogenesis. The relevant experimental data (such as molecular docking, immunofluorescence, and Western Blot) can be found in the Supplementary Materials.

## Figures and Tables

**Table 1:** Molecular docking results for RA and top 50 genes with the largest differences in RNA-Seq results. (↓ indicates down-regulation of gene expression levels, ↑ indicates up-regulation of gene levels).

NO.	Gene	Protein Names	Total Score	Expression Level	Regulation Direction	NO.	Gene	Protein Names	Total Score	Expression Level	Regulation Direction
1	*FGF21*	FGF21	5.07	−1.275093649	↓	26	*CA12*	CAXII	5.81	−1.653209118	↓
2	*GSTM5*	GSTM5	4.77	−3.89975587	↓	27	*MATN3*	MATN3	5.26	−1.649267097	↓
3	*CNTN2*	TAG-1	6.44	−2.994323357	↓	28	*CLEC3B*	TETRANECTIN	5.47	−1.568581179	↓
4	*CLEC3A*	CLEC3A	3.73	−2.965716164	↓	29	*KRT15*	KRT15	4.53	−1.535326314	↓
5	*RPS29*	RPS29	3.4	2.002043011	↓	30	*ENHO*	ADROPIN	2.01	−1.513834939	↓
6	*ARMCX1*	ALEX1	5.18	−2.51049428	↓	31	*ACAN*	ACAN	7.64	−1.508346707	↓
7	*SFRP2*	SFRP2	6.96	−2.487056425	↓	32	*ABI3BP*	ABI3BP	5.47	−1.481523265	↓
8	*KANSL2*	NSL2	6.97	−2.469070952	↓	33	*COL27A1*	COL27A1	6.04	−1.426276819	↓
9	*ART3*	ART3	5.31	−2.436647379	↓	34	*CNMD*	CHM	3.26	−1.425761422	↓
10	*CHRDL2*	CHRDL2	4.66	−2.350714841	↓	35	*MGLL*	MGLL	0.83	−1.406530896	↓
11	*SNX22*	SNX22	4.11	−2.261867748	↓	36	*ANXA8*	ANXA8	5.61	−1.404279806	↓
12	*SMPD3*	SMPD3	4.85	−2.152491655	↓	37	*IGSF11*	IGSF11	4.1	−1.366905518	↓
13	*MAGEL2*	MAGE-L2	4.81	−1.998949503	↓	38	*ERG*	ERG	4.92	−1.359516692	↓
14	*SMIM5*	TMBIM5	3.19	−1.960175369	↑	39	*INHBE*	INHBE	5.74	−1.351823894	↓
15	*SLC38A3*	SNAT3	5.44	−1.920830616	↓	40	*RASSF4*	RASSF4	5.19	−1.35158272	↓
16	*COLGALT2*	COLGALT2	5.36	−1.913873314	↓	41	*CRYGA*	GAMMA-CRYSTALLIN A	5.36	−1.338521637	↓
17	*LYPD6B*	LYPD6B	4.96	−1.860922982	↓	42	*NCMAP*	NCMAP	3.17	−1.338181175	↓
18	*EPYC*	DSPG3	3.05	−1.750839641	↓	43	*PCOLCE2*	PCPE2	4.62	−1.330137352	↓
19	*RPL32*	RPL32	3.88	−1.748371841	↓	44	*C1QTNF3*	CTRP3	4.06	−1.32979187	↑
20	*GRAMD2A*	GRAMD2A	3.97	−1.698220337	↓	45	*NR0B1*	DAX1	3.2	−1.323829573	↑
21	*RPL35A*	RPL35A	5.65	−1.685224194	↓	46	*MIA*	MIA	4.13	−1.312747989	↓
22	*ZNF648*	ZNF648	4.66	−1.667062981	↓	47	*HAPLN1*	HAPLN1	5.84	−1.299373455	↓
23	*FGFR4*	FGFR4	5.07	−1.664759977	↓	48	*GLIS1*	GLIS1	4.43	−1.290628079	↓
24	*KCTD4*	KCTD4	4.55	−1.663897339	↓	49	*EEF1AKMT3*	METTL21B	3.79	−1.286673416	↓
25	*FASLG*	TNFSF6	4.22	−1.657606623	↓	50	*POSTN*	POSTN	5.73	−1.285222679	↓

**Table 2:** Molecular docking results for 42 intersecting targets of RA targets and OA targets.

NO.	Gene	Protein Names	Total Score	NO.	Gene	Protein Names	Total Score
1	*RXRB*	RXRB	4.98	22	*AGTR1*	AT1R	4.59
2	*RARG*	RARG	4.51	23	*PTGER3*	EP3R	6.64
3	*RARB*	RARB	6.33	24	*PGR*	PGR	5.82
4	*ADORA3*	ADORA3	5.56	25	*HNF4A*	HNF4α	6.28
5	*MAPK1*	MAPK1	6.93	26	*PLA2G4A*	cPLA2α	4.54
6	*RORA*	RORA	4.33	27	*DRD2*	DRD2	4.53
7	*MAPK14*	p38α	8.01	28	*PTGIR*	IPR	6.25
8	*PTPN1*	PTP1B	4.69	29	*PTGS1*	COX-1	5.74
9	*RBP4*	RBP4	4.93	30	*PTGS2*	COX-2	6.14
10	*PPARA*	PPARA	6.29	31	*CMA1*	CMA1	5.04
11	*SLC16A1*	MCT1	4.96	32	*FFAR4*	GPR120	5.89
12	*PPARG*	PPARG	6.5	33	*CA2*	CAII	7.44
13	*CYP26B1*	Cyp26B1	6.28	34	*HMGCR*	HMGCR	5.79
14	*PTGER4*	EP4	5.63	35	*CCR2*	CCR2	6.91
15	*PTGER2*	PTGER2	4.74	36	*ESR1*	ERα	6.29
16	*PTGES2*	PGES2	5.67	37	*ESR2*	ERβ	4.7
17	*LTB4R*	BLT1	4.75	38	*TP53*	p53	5.7
18	*PTGDR2*	CRTH2	5.24	39	*ACE*	ACE	6.54
19	*MDM2*	MDM2	5.57	40	*STS*	STS	5.99
20	*CCR9*	CCR9	6	41	*EDNRA*	ETAR	4.82
21	*AGTR2*	AT2R	6.93	42	*NCSTN*	NCSTN	5.9

**Table 3:** Primers for RT-qPCR.

Genes	Sequences
*PGR*	F: 5′-ACAAAGCCCGACACTTCC-3′
	R: 5′-CCTCTCGCCTAGTTGGTTG-3′
*CA2*	F: 5′-TACCTCTCACCCATTGTGC-3′
	R: 5′-GCCCTAAGTCAACCACCA-3′
*EDNRA*	F: 5′-GGCCCTTGGAGACCTTAT-3′
	R: 5′-GGAACAGCTTGCAGAGAAA-3′
*ACE*	F: 5′-AGAGTCCAGTCGCGTCAAC-3′
	R: 5′-GGAAGAGCAGCACCCACT-3′

Note: *PGR*: Progesterone Receptor; *CA2*: Carbonic Anhydrase 2; *EDNRA*: Endothelin Receptor Type A; *ACE*: Angiotensin Converting Enzyme; RT-qPCR: real-time fluorescence quantitative PCR.

## Data Availability

The data presented in this study are available on request from the corresponding author.

## Supplementary Materials

The following supporting information can be downloaded at: https://www.mdpi.com/article/10.3390/ijms26125519/s1.

## Abbreviations

*ACE*	Angiotensin Converting Enzyme
APL	Acute promyelocytic leukemia
BBB	Blood–brain barrier
ATRA	All-trans retinoic acid
BCO1	β-carotene oxygenase 1
BP	Biological processes
*CA2*	Carbonic Anhydrase 2
CC	Cellular components
*COL2A1*	Collagen type II alpha 1 chain
DEGs	Differentially expressed genes
DMEM	Dulbecco’s Modified Eagle’s Medium
ECM	Extracellular matrix
EDNRA	Endothelin Receptor Type A
FC	Fold change
GO	Gene ontology
KEGG	Kyoto Encyclopedia of Genes and Genomes
MF	Molecular function
*MMP13*	Matrix metalloproteinase 13
OA	Osteoarthritis
*PGR*	Progesterone Receptor
PPI	Protein-protein interaction
*PTGS1*	Prostaglandin-Endoperoxide Synthase 1
RA	Retinoic acid
RNA-Seq	RNA-sequencing
RP	Ribosomal Proteins
RPT	Receptor Type Protein Tyrosine Phosphatases
RS	Recursive Splicing
SPF	Specific pathogen-free
TGFα	Transforming growth factor alpha
siRNA	small interfering RNA
