# Supplementary figures and images for: Study of Retinoic Acid-Induced Osteoarthritis: Integrating RNA-Sequencing, Network Pharmacology, Molecular Docking, and Experimental Validation
Source: Int J Mol Sci. 2025 Jun 9;26(12):5519. doi: 10.3390/ijms26125519 (PMC12192781; doi:10.3390/ijms26125519)

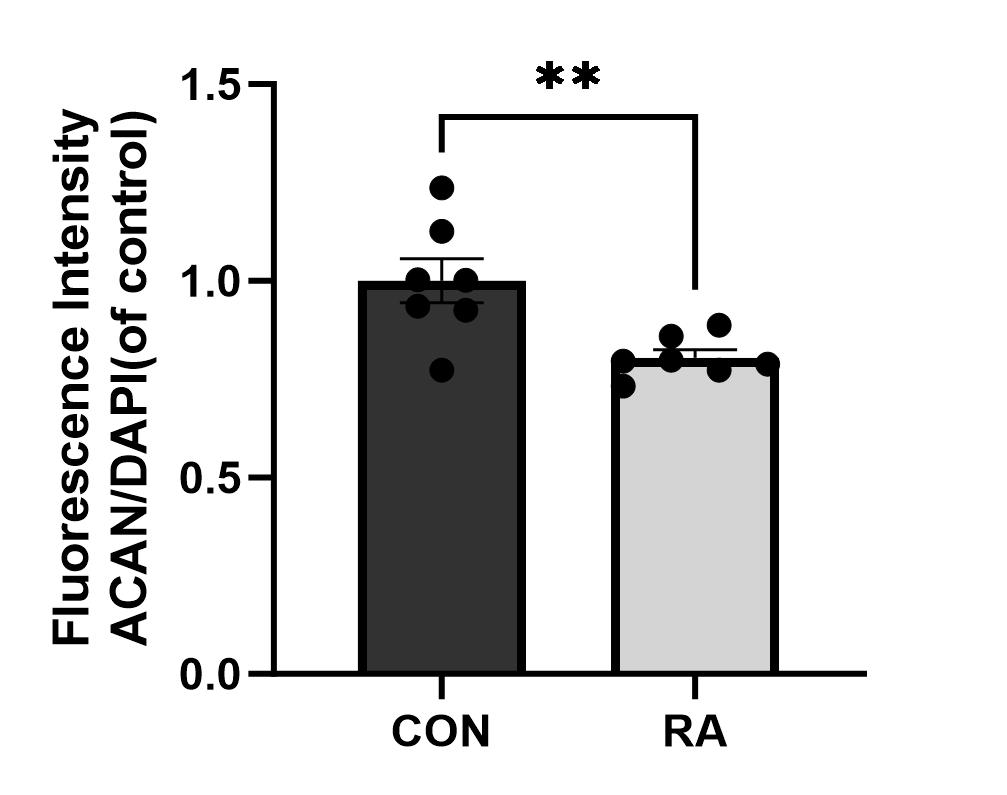

Supplement: Supplementary file 1 [file ijms-26-05519-s001.zip › Supplementary Materials/Supplementary Material S2/ACAN data.jpg]

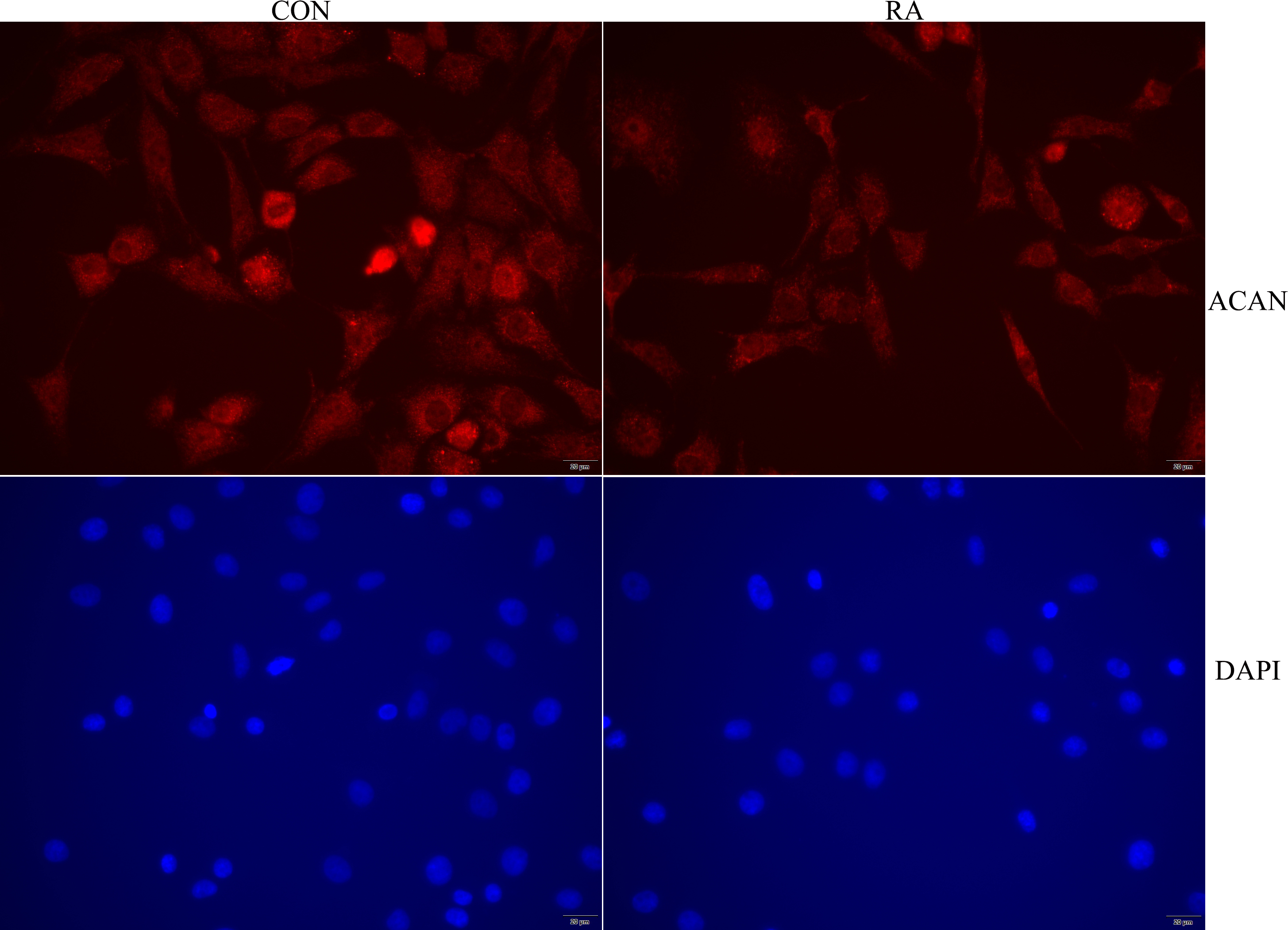

Supplement: Supplementary file 1 [file ijms-26-05519-s001.zip › Supplementary Materials/Supplementary Material S2/ACAN.jpg]

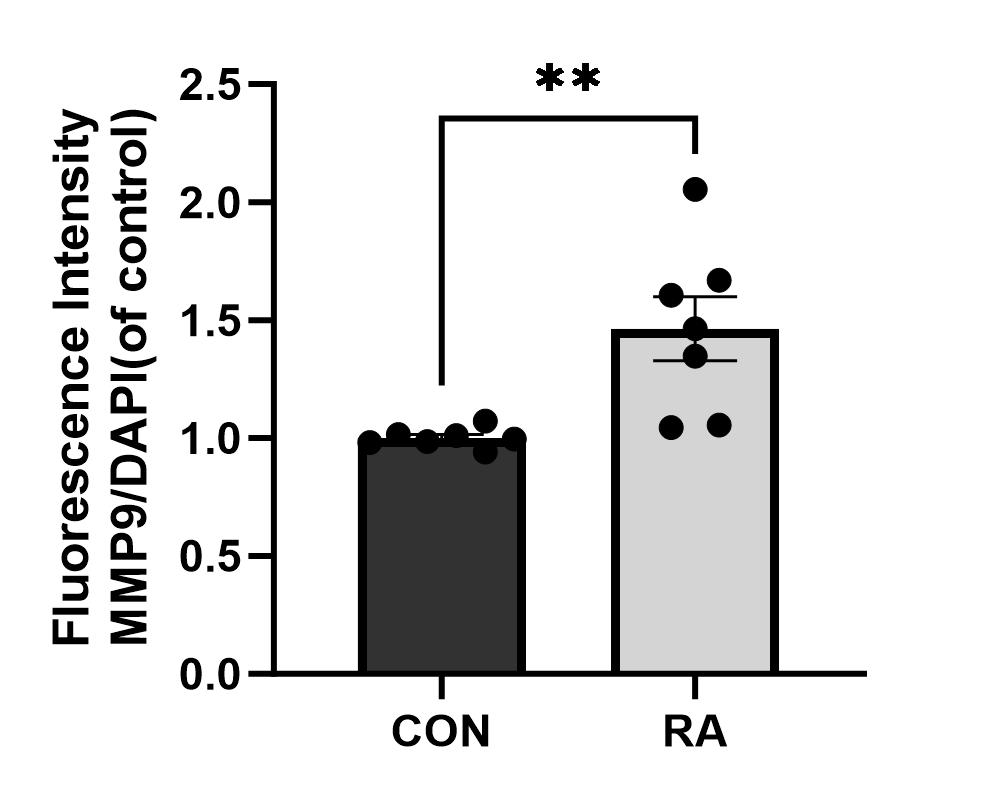

Supplement: Supplementary file 1 [file ijms-26-05519-s001.zip › Supplementary Materials/Supplementary Material S2/MMP9 data.jpg]

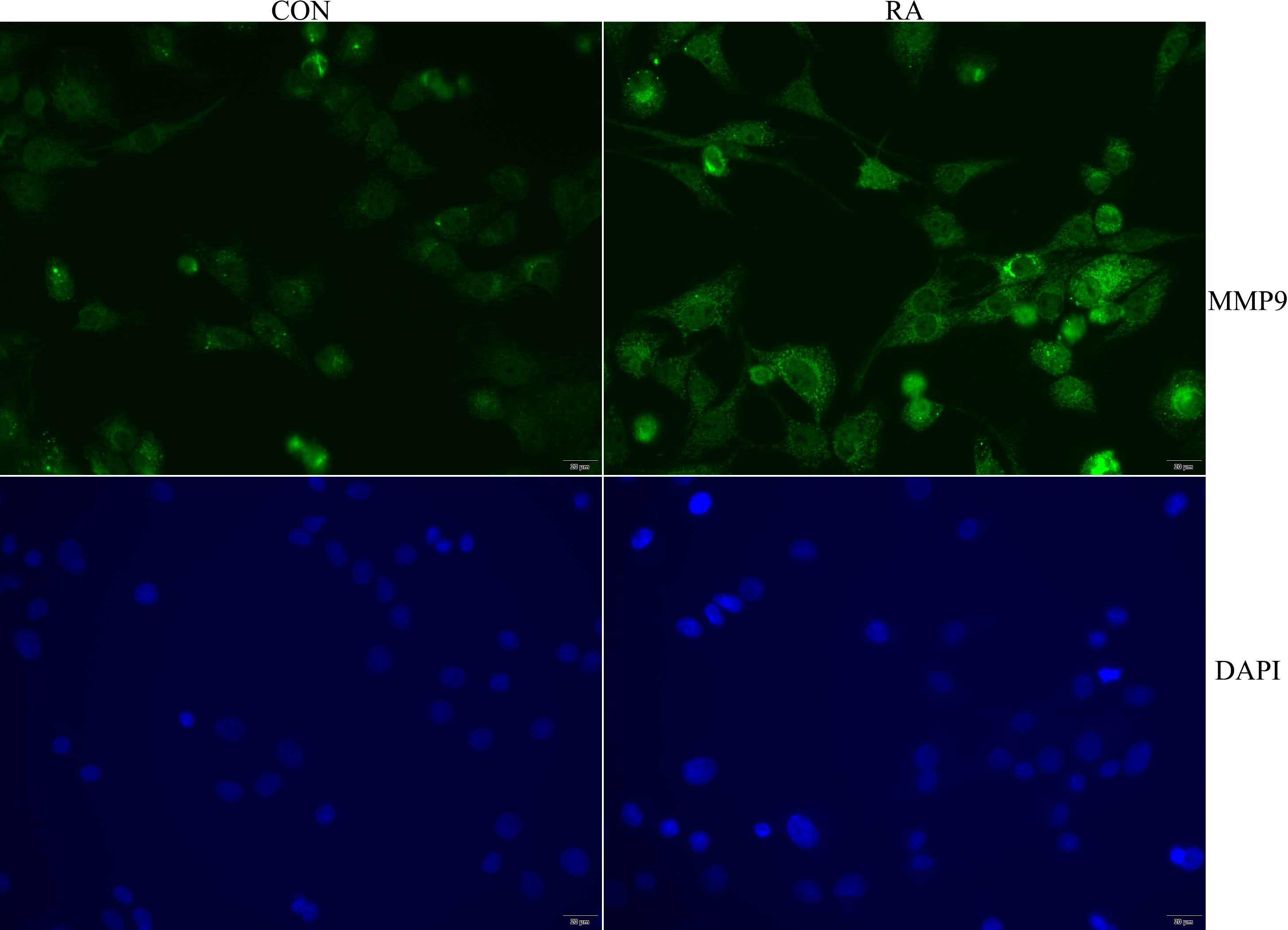

Supplement: Supplementary file 1 [file ijms-26-05519-s001.zip › Supplementary Materials/Supplementary Material S2/MMP9.jpg]

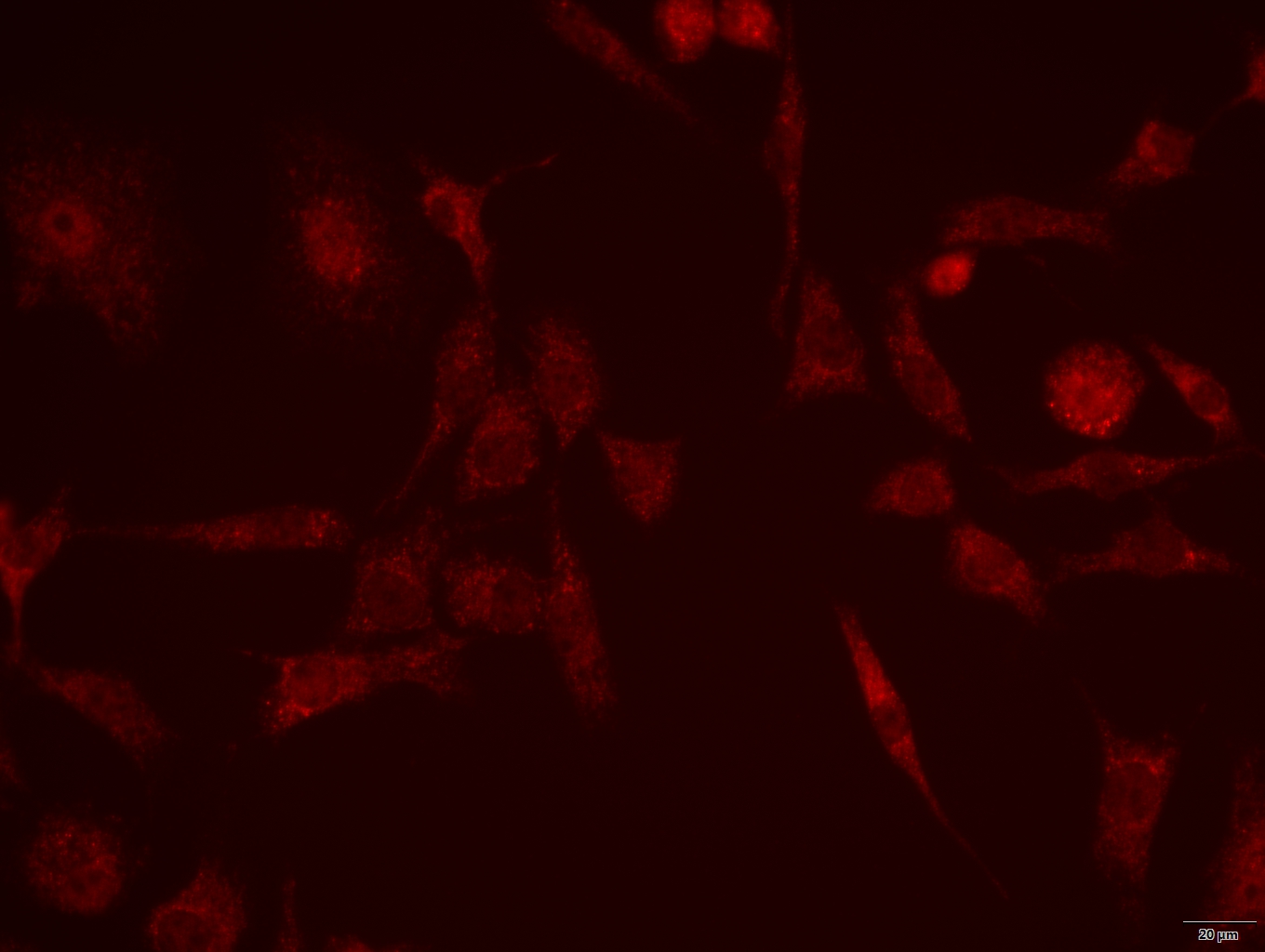

Supplement: Supplementary file 1 [file ijms-26-05519-s001.zip › Supplementary Materials/Supplementary Material S2/origin/acan/RA-acan.jpg]

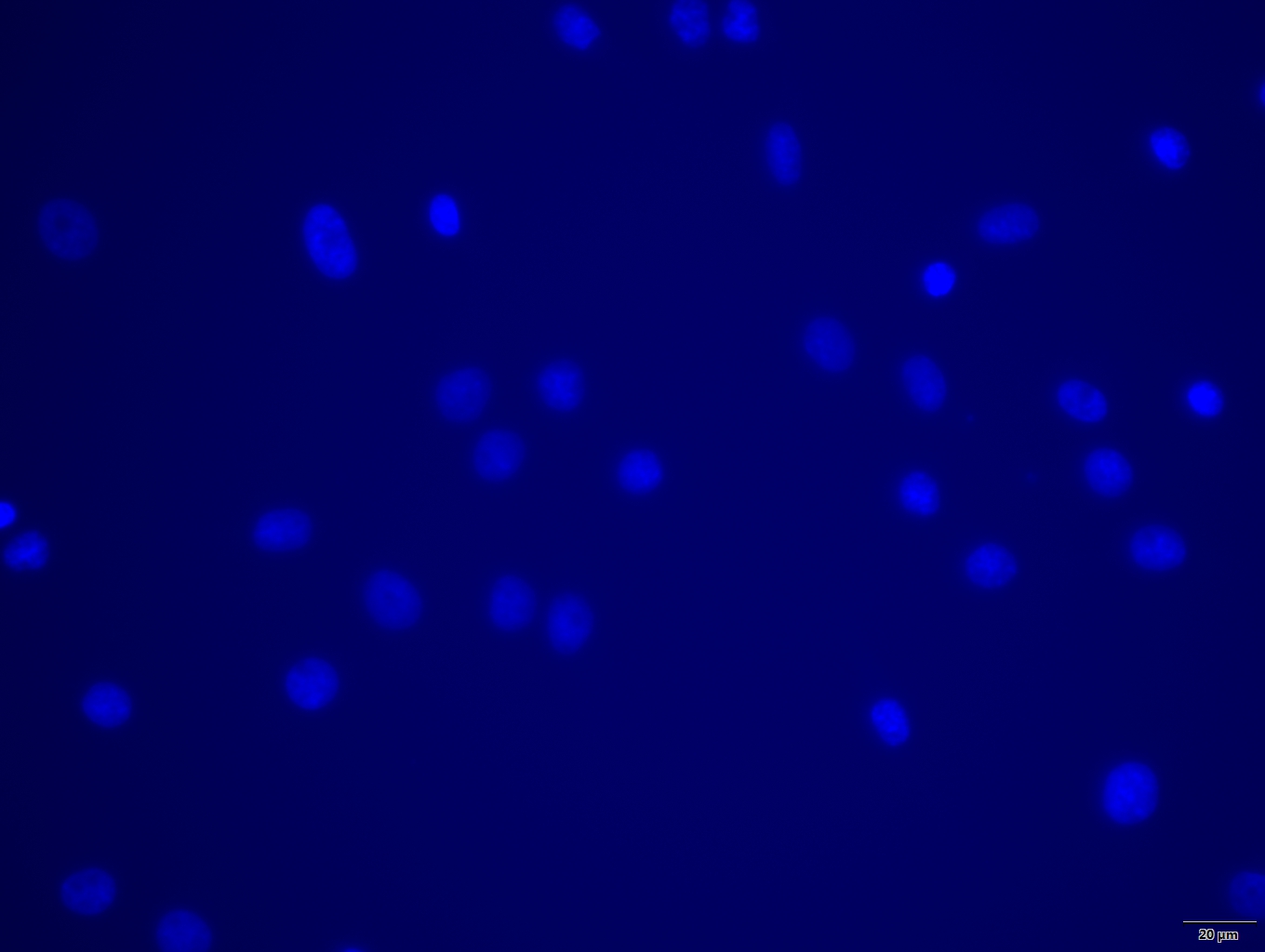

Supplement: Supplementary file 1 [file ijms-26-05519-s001.zip › Supplementary Materials/Supplementary Material S2/origin/acan/RA-DAPI.jpg]

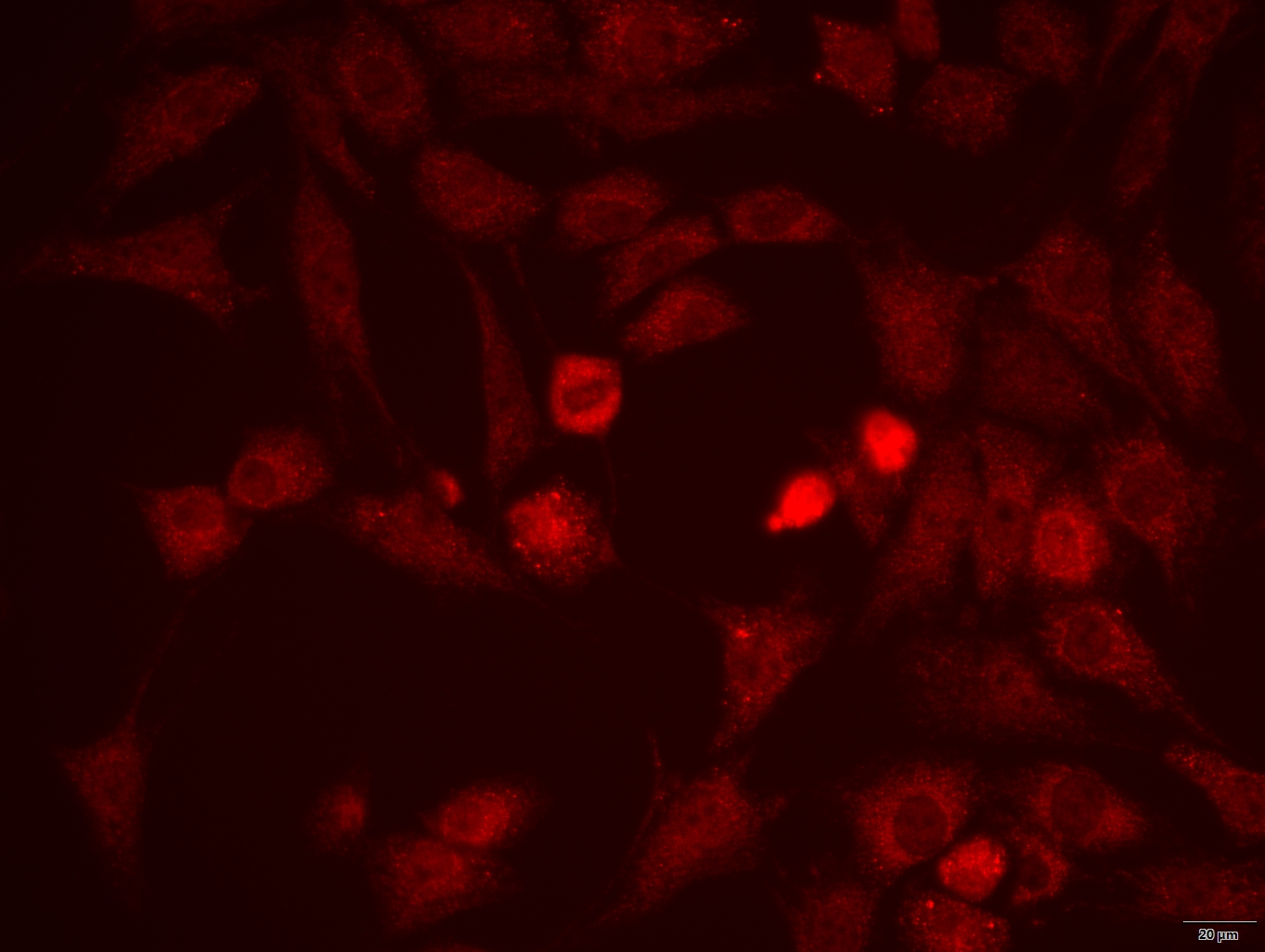

Supplement: Supplementary file 1 [file ijms-26-05519-s001.zip › Supplementary Materials/Supplementary Material S2/origin/acan/对照-acan.jpg]

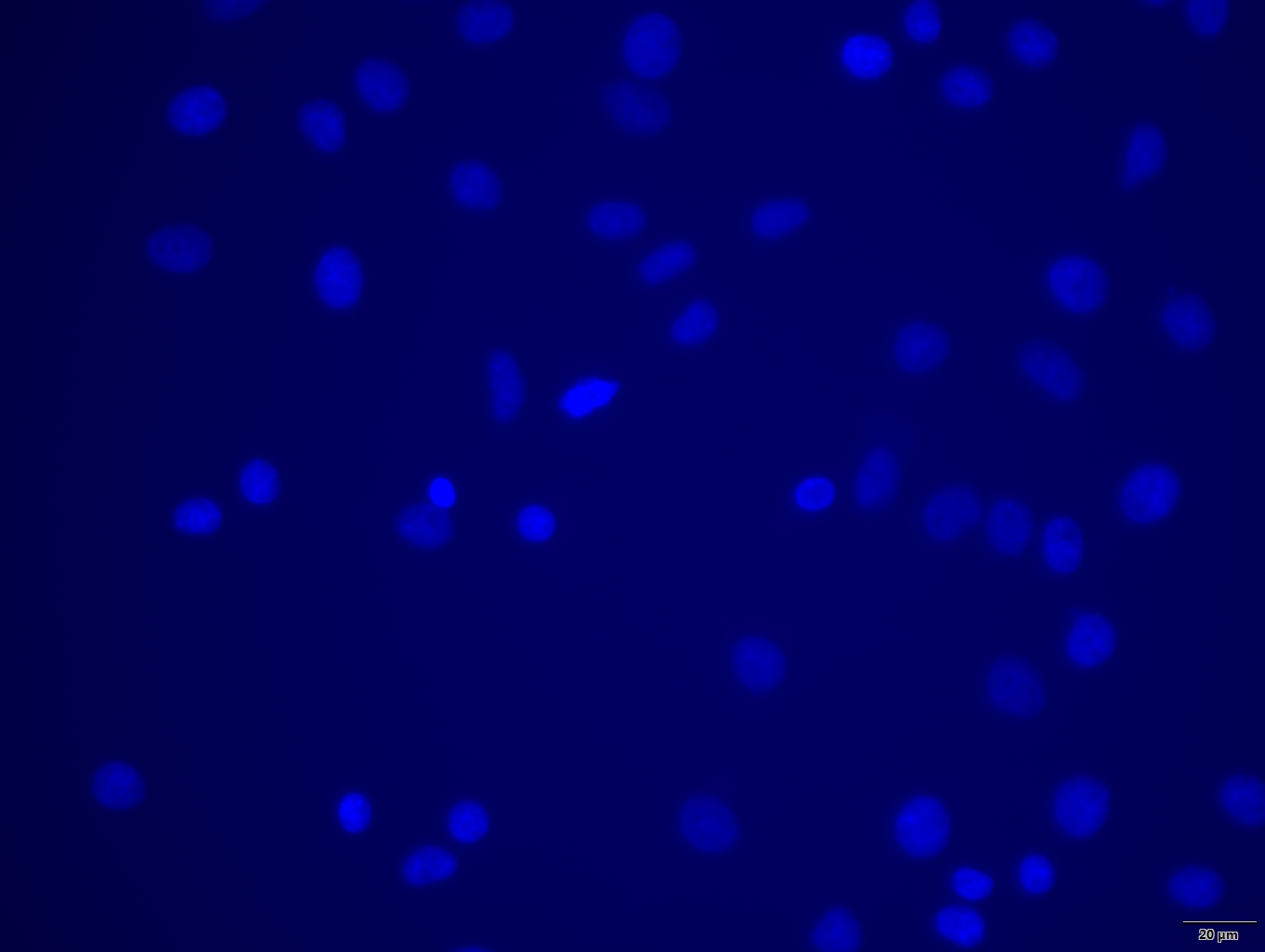

Supplement: Supplementary file 1 [file ijms-26-05519-s001.zip › Supplementary Materials/Supplementary Material S2/origin/acan/对照DAPI.jpg]

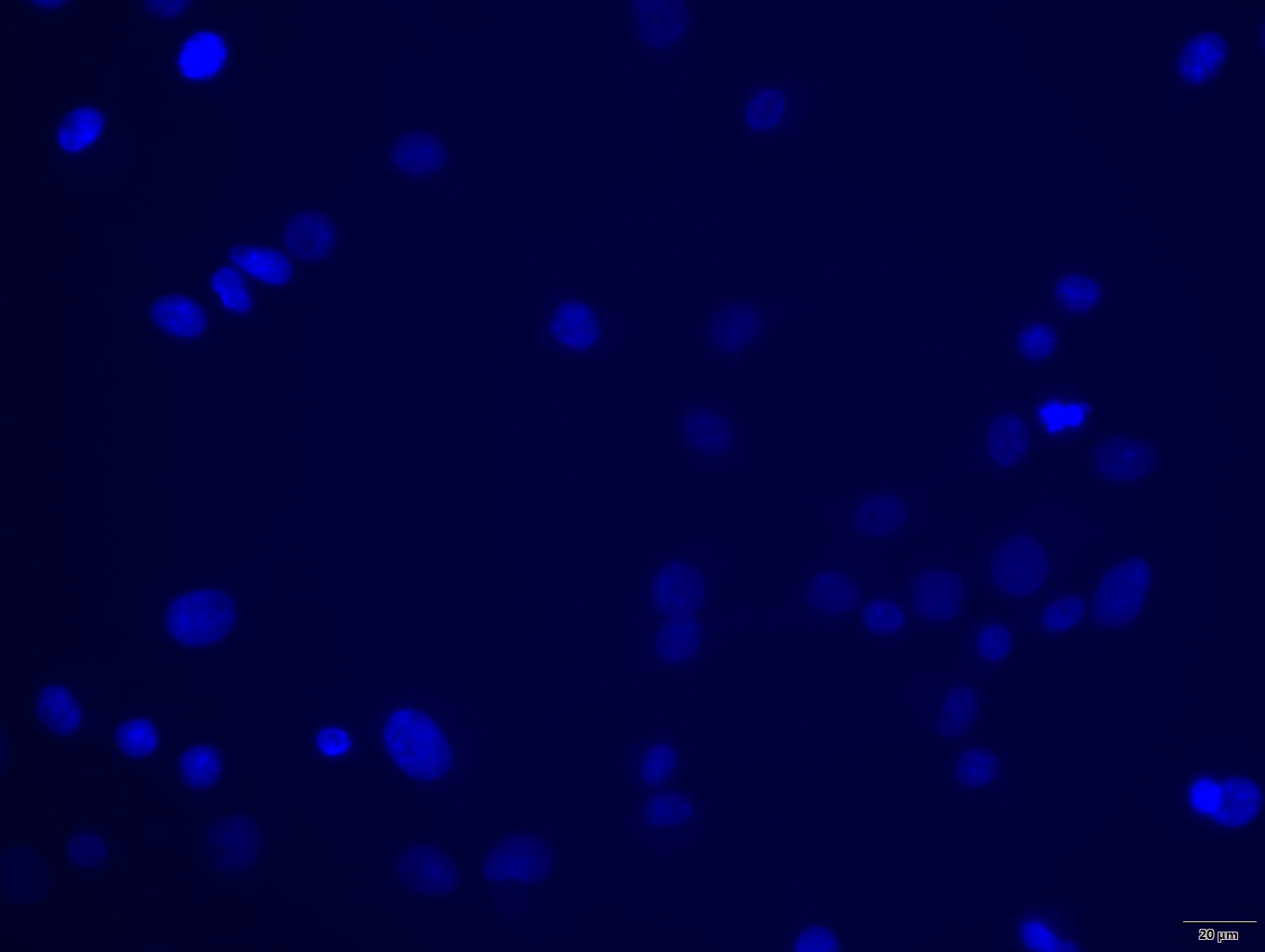

Supplement: Supplementary file 1 [file ijms-26-05519-s001.zip › Supplementary Materials/Supplementary Material S2/origin/mmp9/RA-DAPI.jpg]

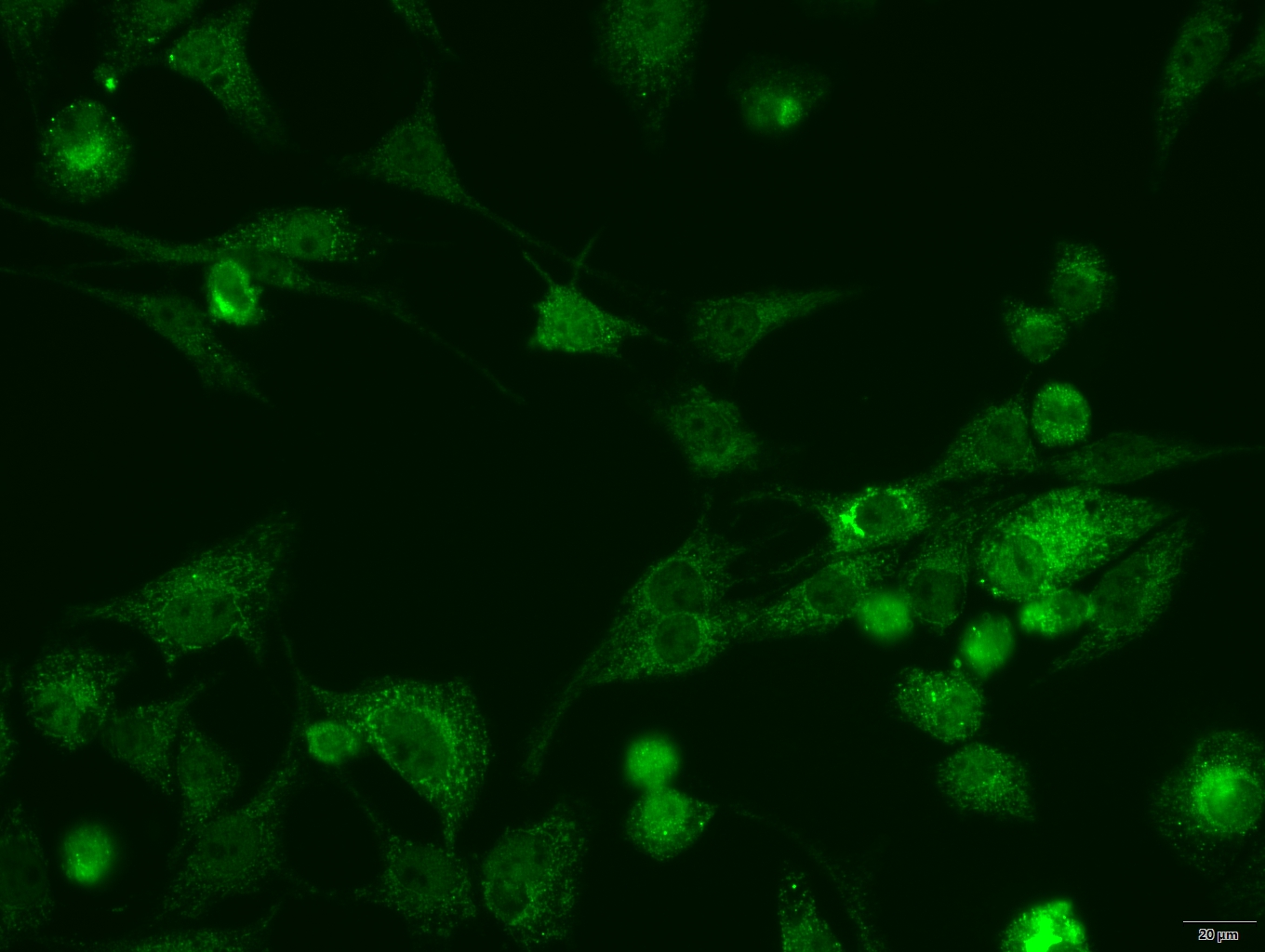

Supplement: Supplementary file 1 [file ijms-26-05519-s001.zip › Supplementary Materials/Supplementary Material S2/origin/mmp9/RA-MMP9.jpg]

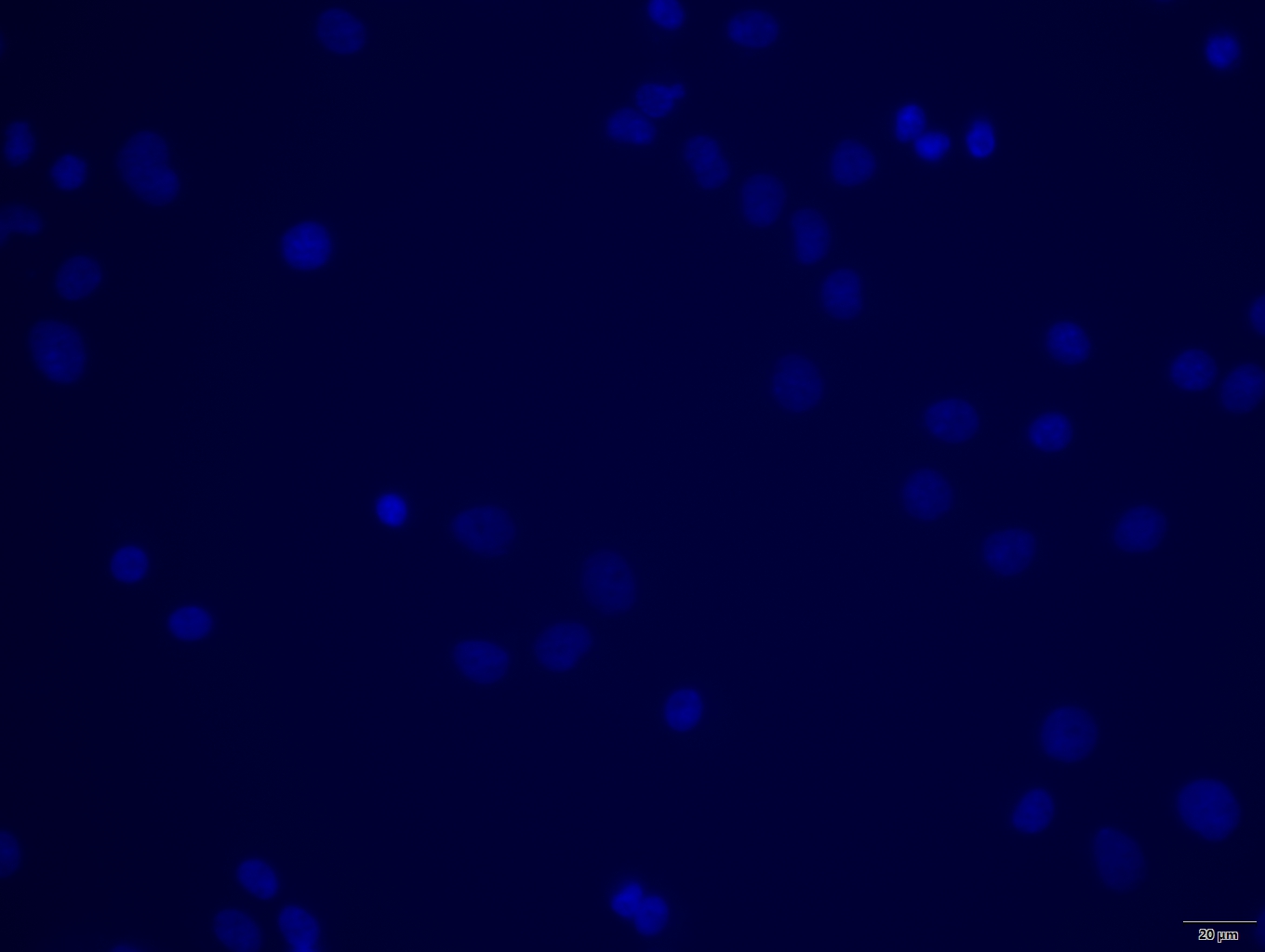

Supplement: Supplementary file 1 [file ijms-26-05519-s001.zip › Supplementary Materials/Supplementary Material S2/origin/mmp9/对照-DAPI.jpg]

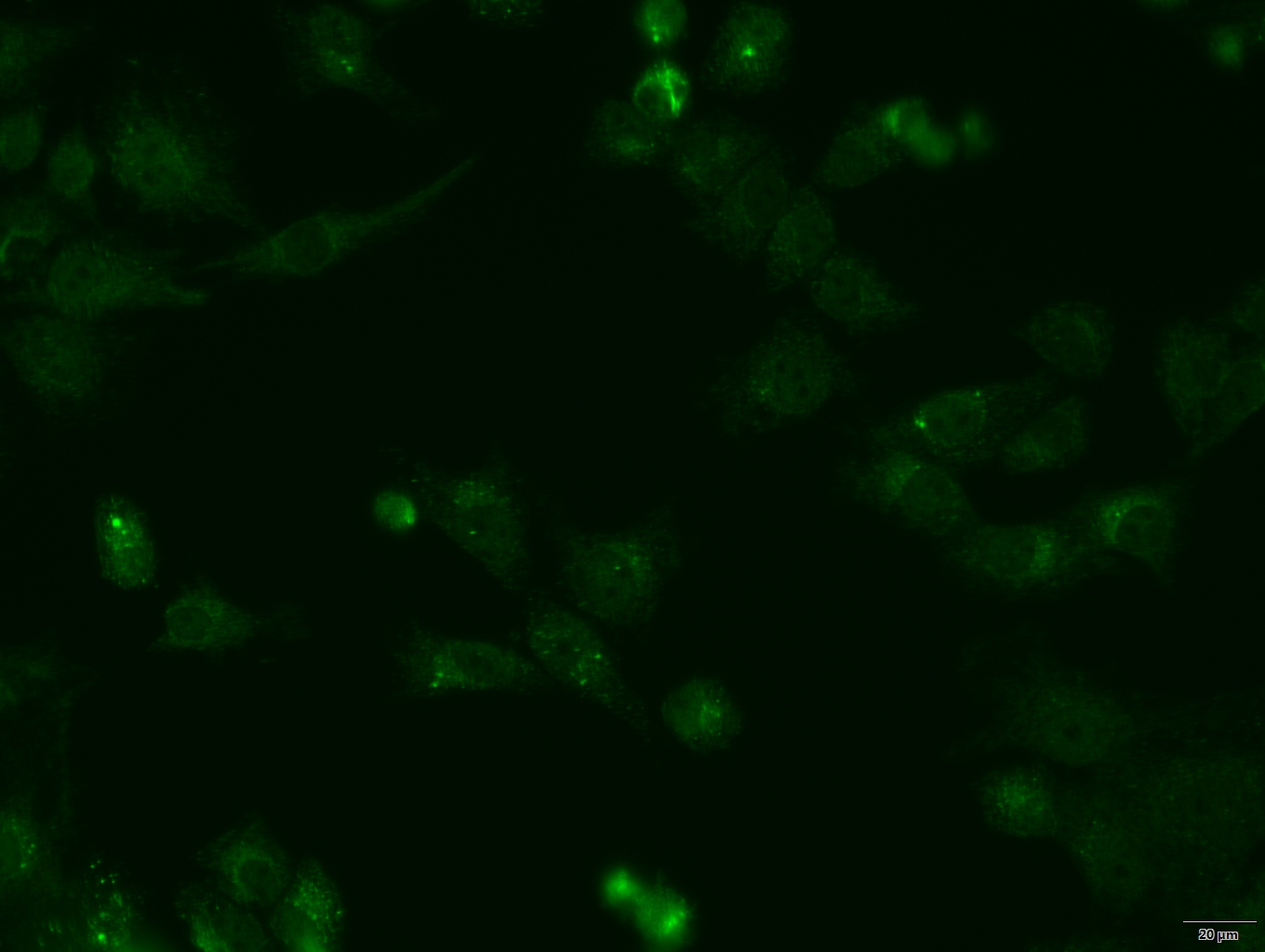

Supplement: Supplementary file 1 [file ijms-26-05519-s001.zip › Supplementary Materials/Supplementary Material S2/origin/mmp9/对照-MMP9.jpg]

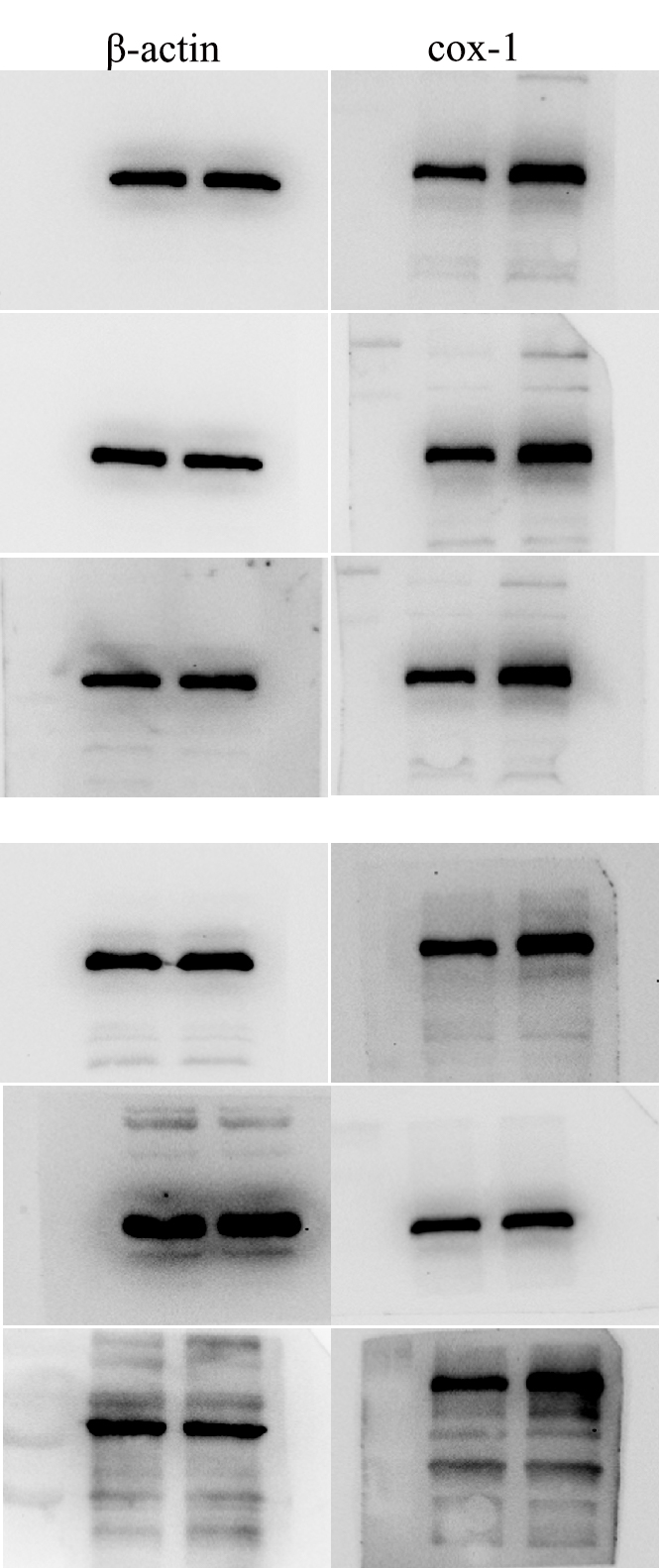

Supplement: Supplementary file 1 [file ijms-26-05519-s001.zip › Supplementary Materials/Supplementary Material S3/combined image.jpg]

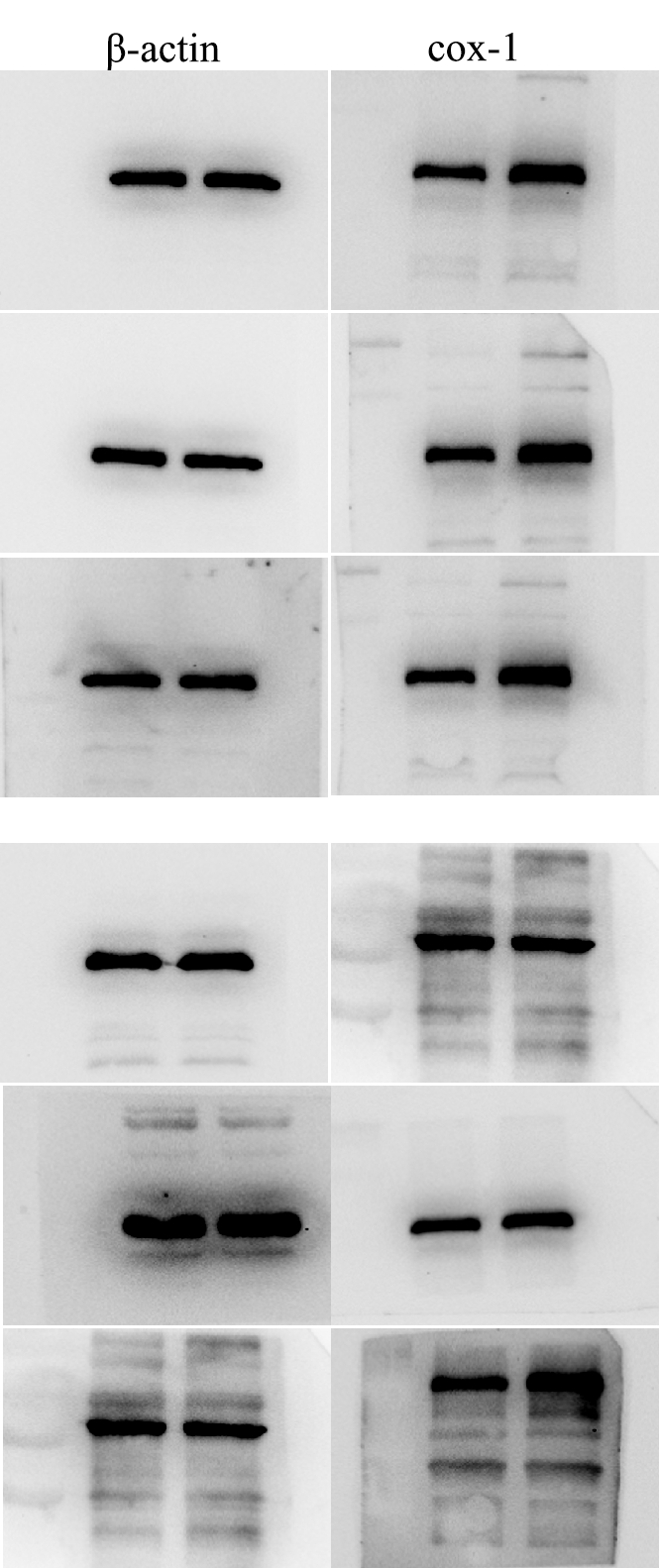

Supplement: Supplementary file 1 [file ijms-26-05519-s001.zip › Supplementary Materials/Supplementary Material S3/data/combined image(analyze).tif]

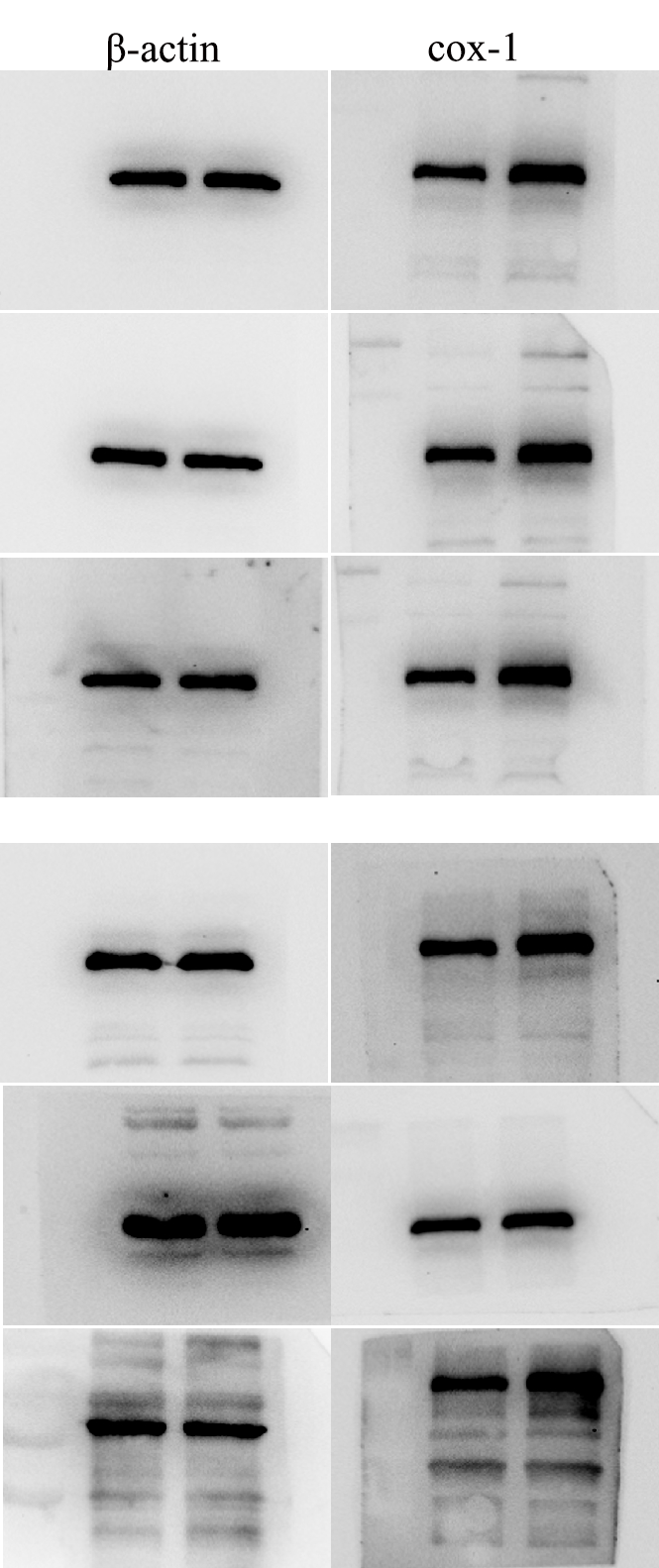

Supplement: Supplementary file 1 [file ijms-26-05519-s001.zip › Supplementary Materials/Supplementary Material S3/data/combined image.png]

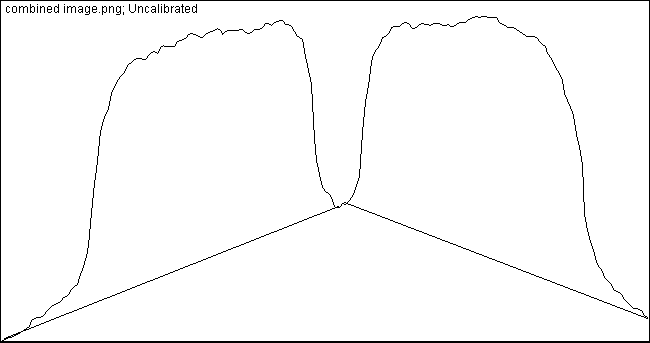

Supplement: Supplementary file 1 [file ijms-26-05519-s001.zip › Supplementary Materials/Supplementary Material S3/data/new actin-1.tif]

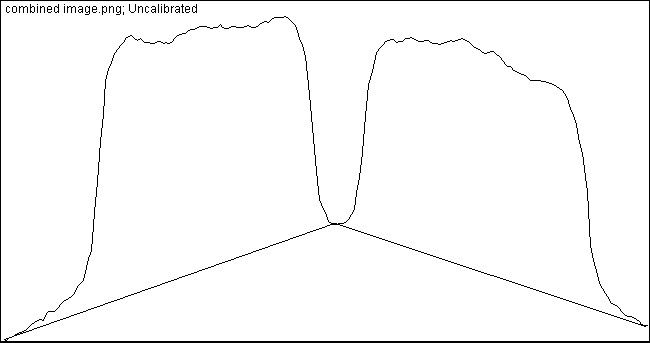

Supplement: Supplementary file 1 [file ijms-26-05519-s001.zip › Supplementary Materials/Supplementary Material S3/data/new actin-2.tif]

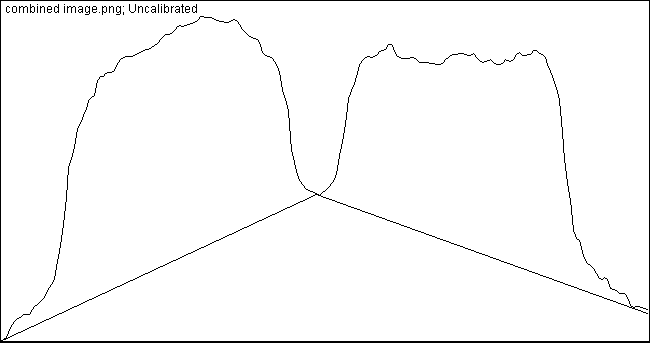

Supplement: Supplementary file 1 [file ijms-26-05519-s001.zip › Supplementary Materials/Supplementary Material S3/data/new actin-3.tif]

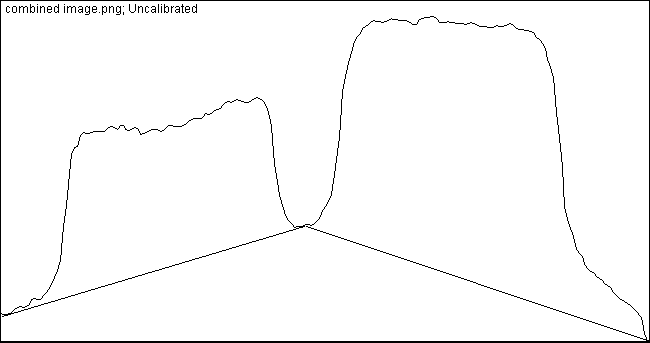

Supplement: Supplementary file 1 [file ijms-26-05519-s001.zip › Supplementary Materials/Supplementary Material S3/data/new cox1-1.tif]

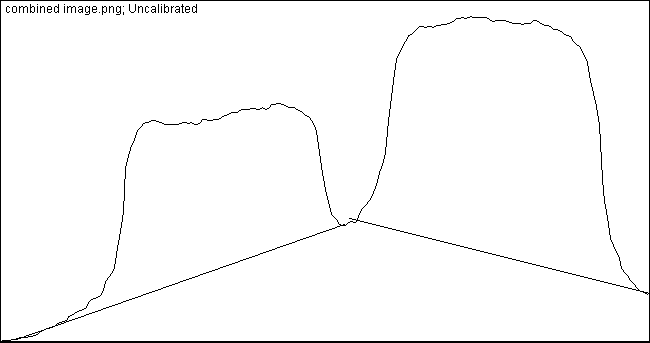

Supplement: Supplementary file 1 [file ijms-26-05519-s001.zip › Supplementary Materials/Supplementary Material S3/data/new cox1-2.tif]

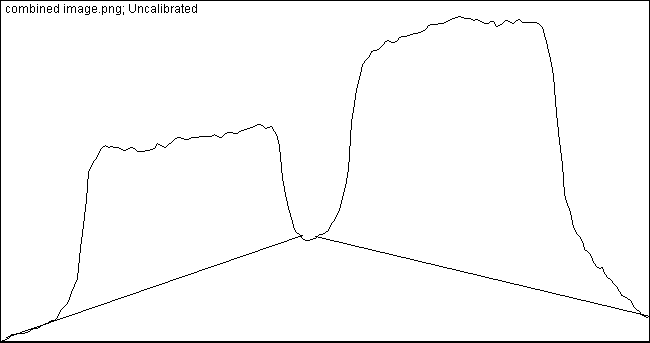

Supplement: Supplementary file 1 [file ijms-26-05519-s001.zip › Supplementary Materials/Supplementary Material S3/data/new cox1-3.tif]

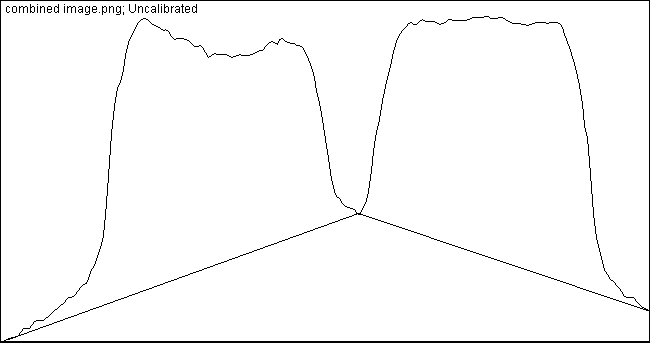

Supplement: Supplementary file 1 [file ijms-26-05519-s001.zip › Supplementary Materials/Supplementary Material S3/data/old actin-1.tif]

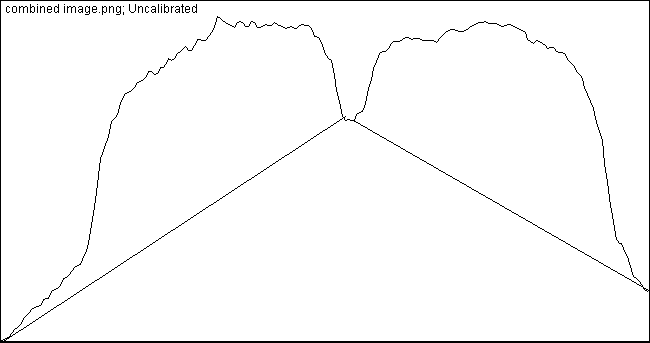

Supplement: Supplementary file 1 [file ijms-26-05519-s001.zip › Supplementary Materials/Supplementary Material S3/data/old actin-2.tif]

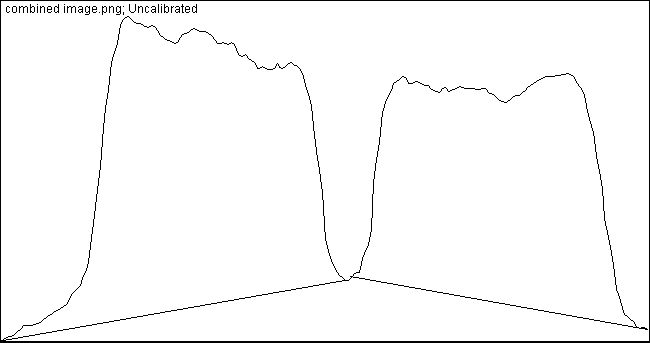

Supplement: Supplementary file 1 [file ijms-26-05519-s001.zip › Supplementary Materials/Supplementary Material S3/data/old actin-3.tif]

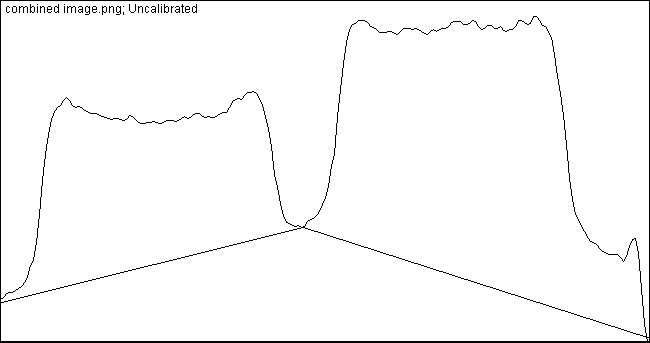

Supplement: Supplementary file 1 [file ijms-26-05519-s001.zip › Supplementary Materials/Supplementary Material S3/data/old cox1-1.tif]

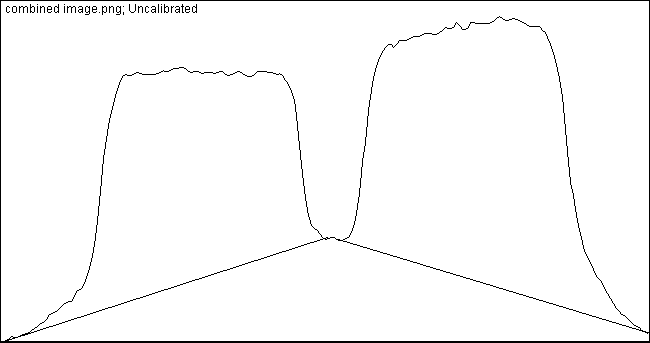

Supplement: Supplementary file 1 [file ijms-26-05519-s001.zip › Supplementary Materials/Supplementary Material S3/data/old cox1-2.tif]

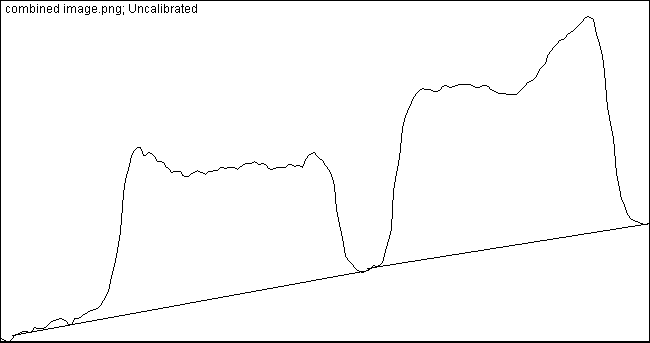

Supplement: Supplementary file 1 [file ijms-26-05519-s001.zip › Supplementary Materials/Supplementary Material S3/data/old cox1-3.tif]

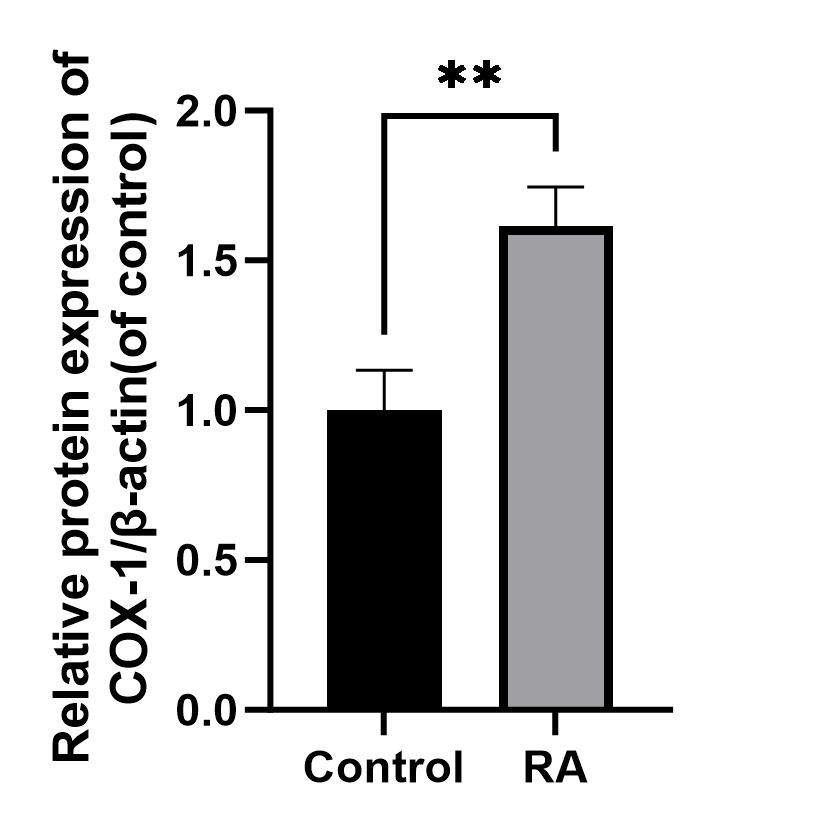

Supplement: Supplementary file 1 [file ijms-26-05519-s001.zip › Supplementary Materials/Supplementary Material S3/data analyze.jpg]

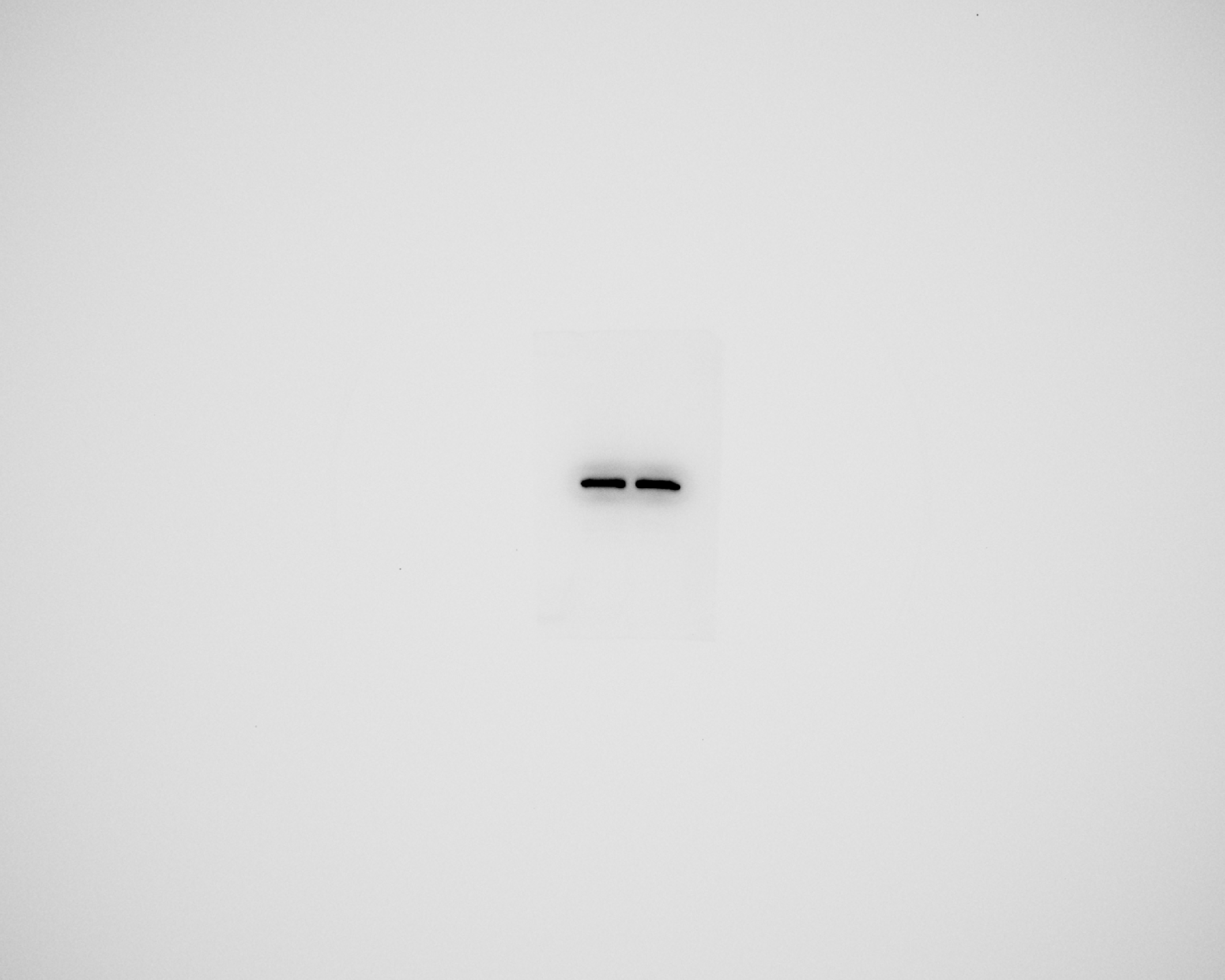

Supplement: Supplementary file 1 [file ijms-26-05519-s001.zip › Supplementary Materials/Supplementary Material S3/New image WB-COX-1/actin 1-Comparison plot.jpg]

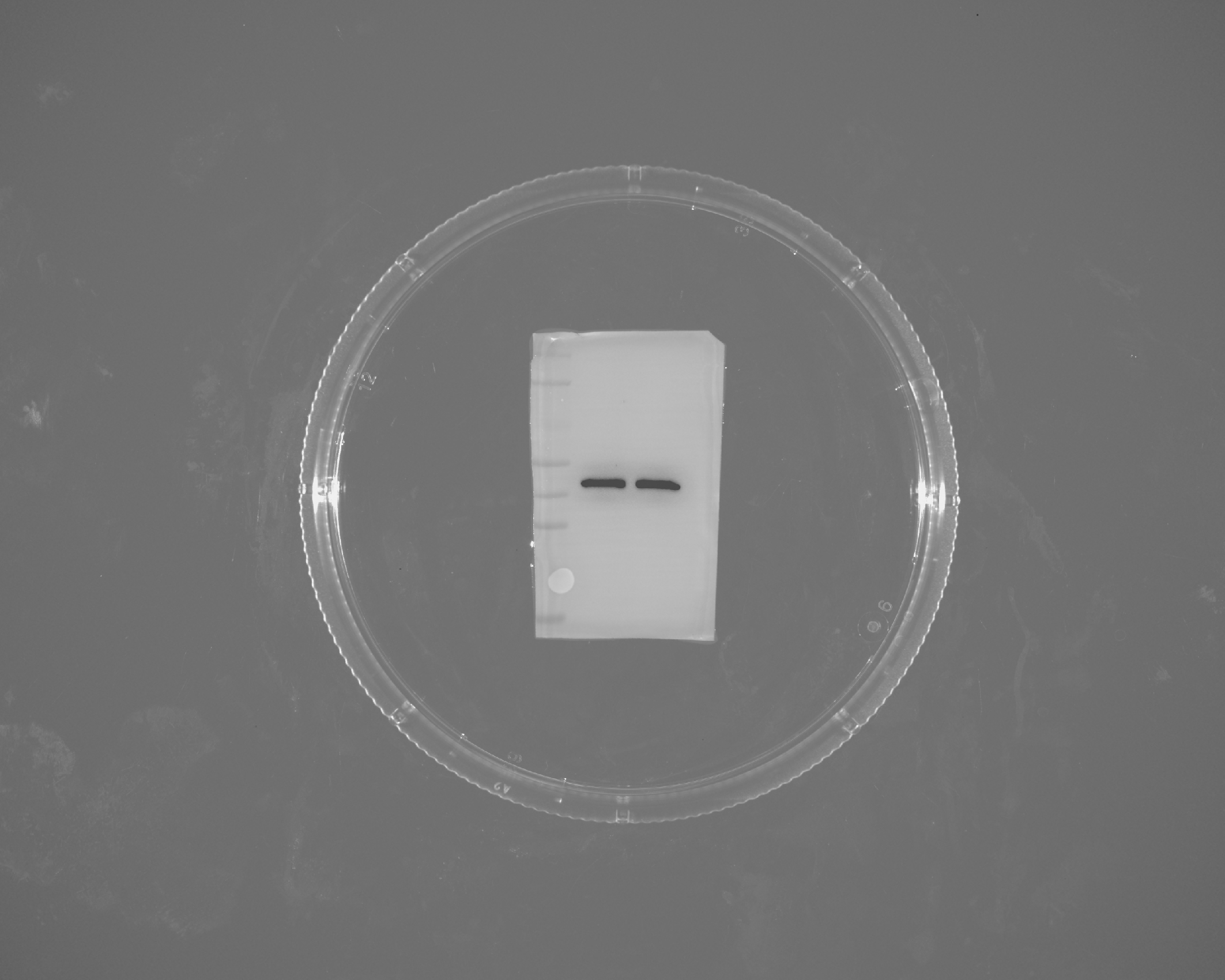

Supplement: Supplementary file 1 [file ijms-26-05519-s001.zip › Supplementary Materials/Supplementary Material S3/New image WB-COX-1/actin 1-Molecular weight marker.jpg]

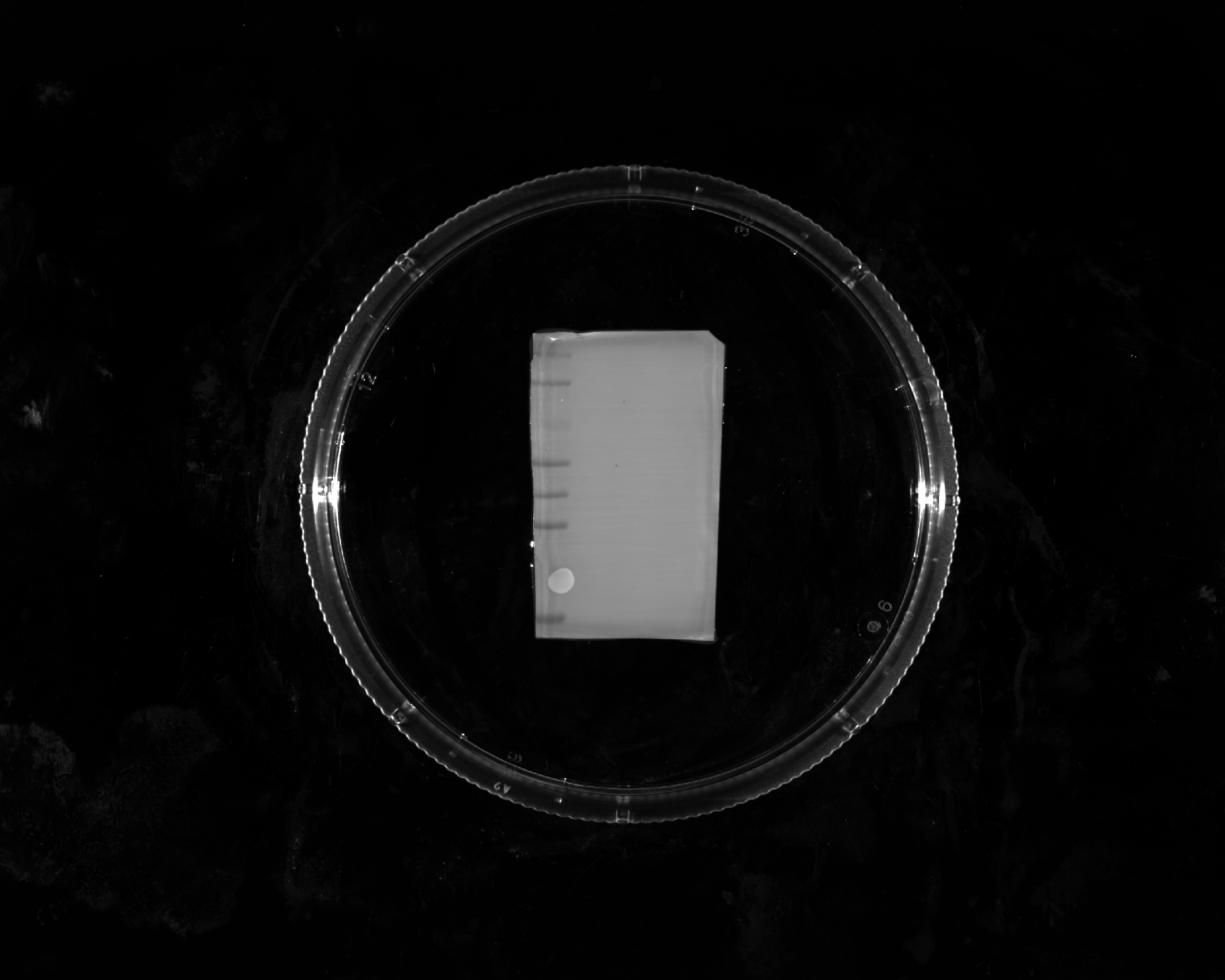

Supplement: Supplementary file 1 [file ijms-26-05519-s001.zip › Supplementary Materials/Supplementary Material S3/New image WB-COX-1/actin 1-Original.jpg]

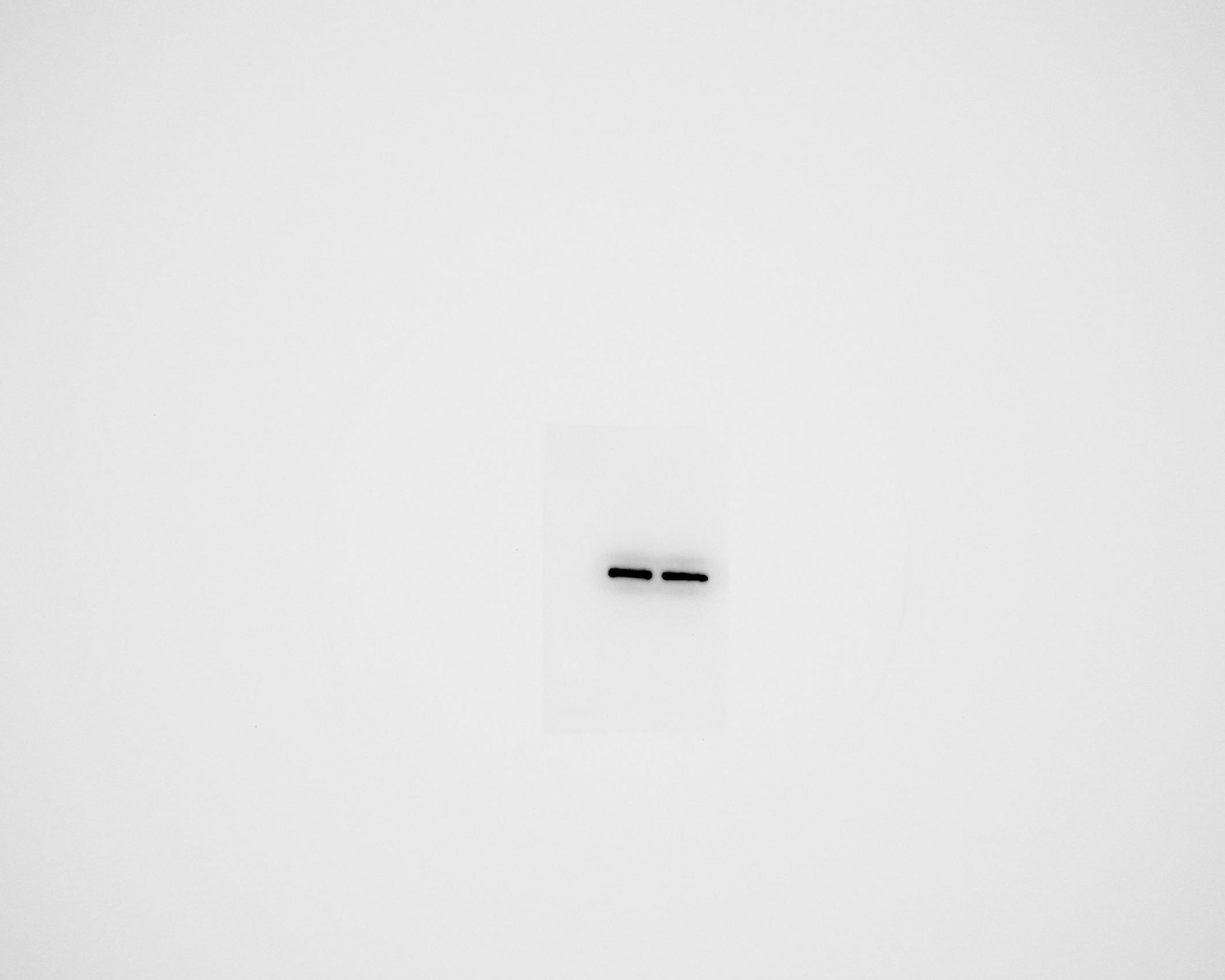

Supplement: Supplementary file 1 [file ijms-26-05519-s001.zip › Supplementary Materials/Supplementary Material S3/New image WB-COX-1/actin 2-Comparison plot.jpg]

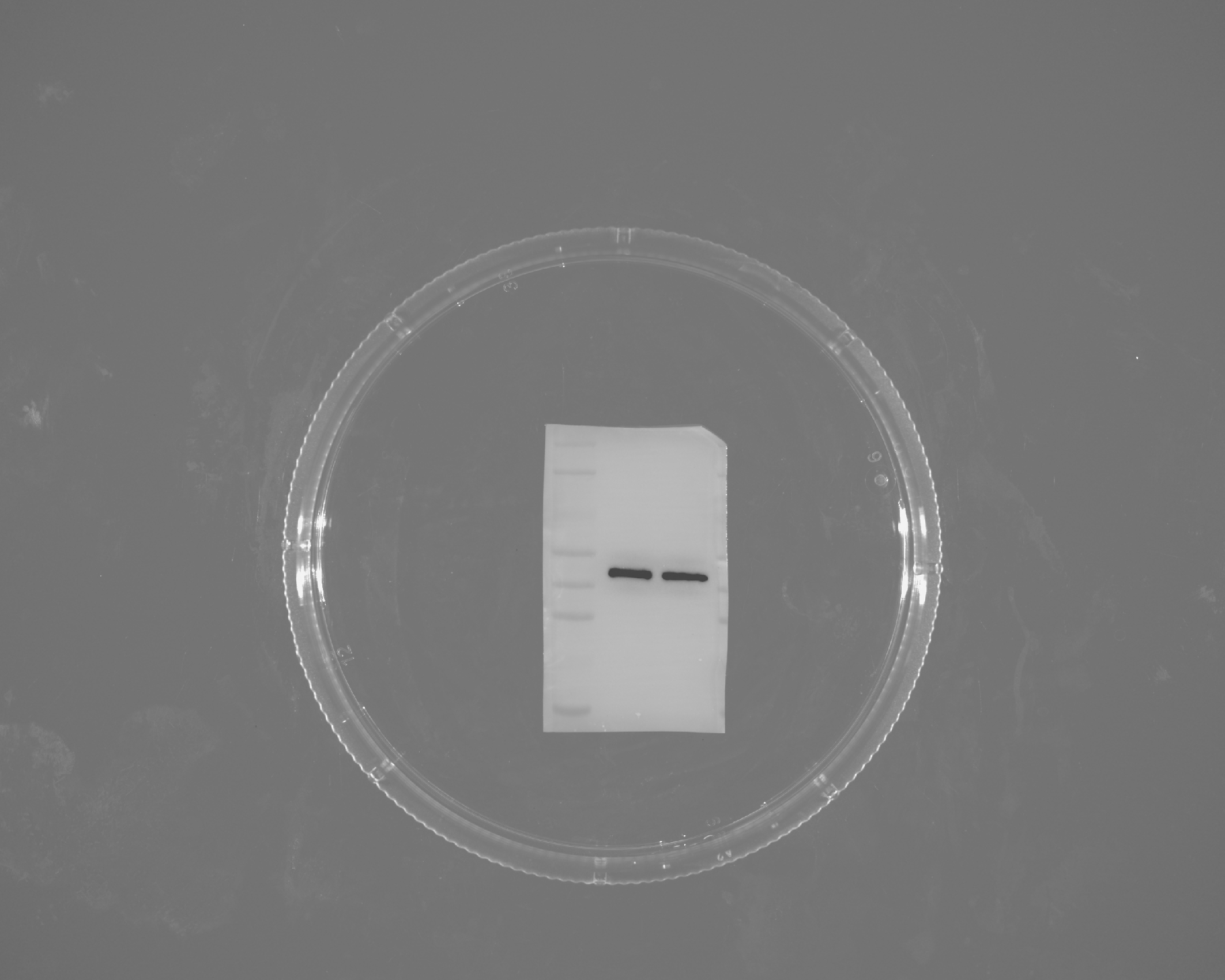

Supplement: Supplementary file 1 [file ijms-26-05519-s001.zip › Supplementary Materials/Supplementary Material S3/New image WB-COX-1/actin 2-Molecular weight marker.jpg]

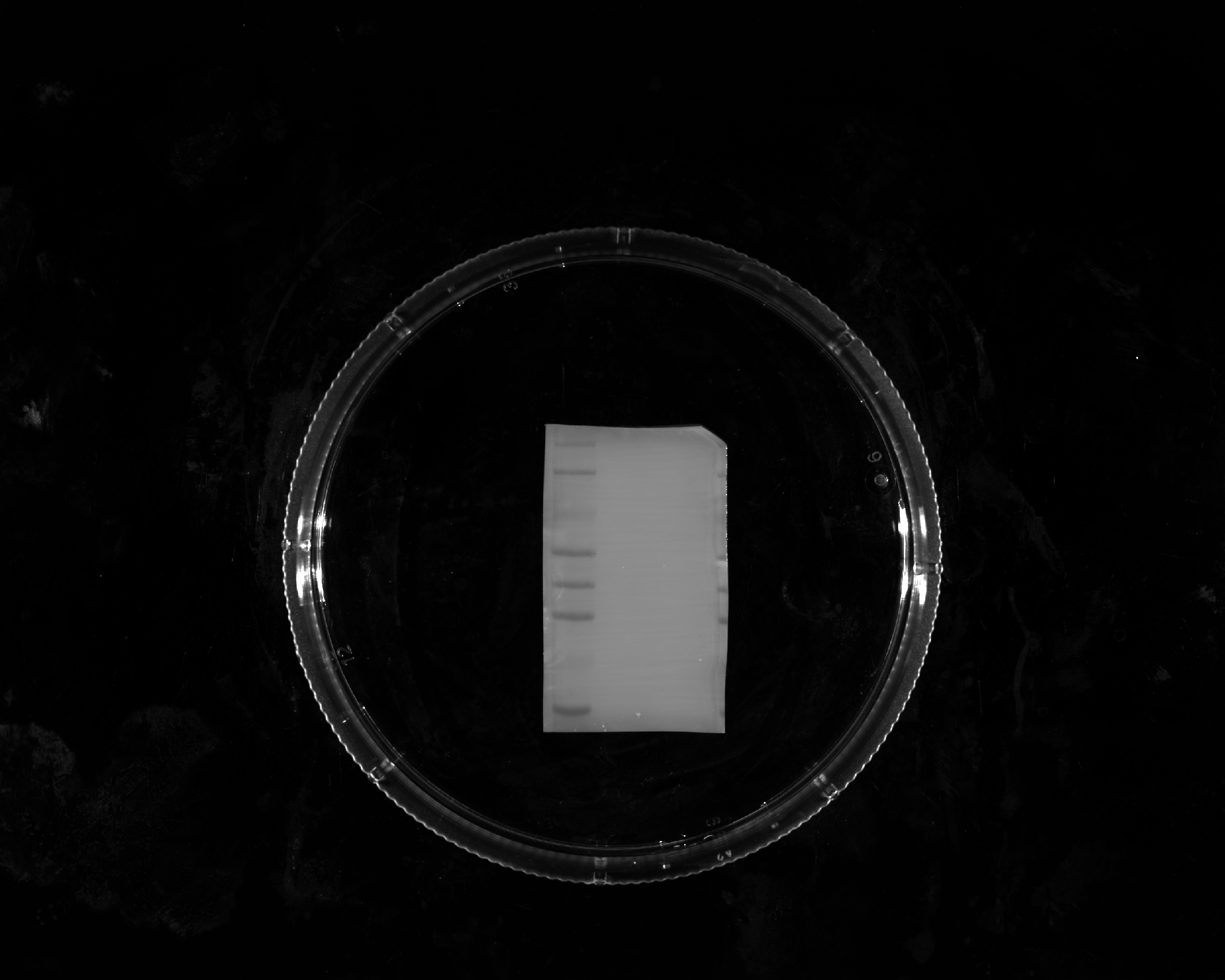

Supplement: Supplementary file 1 [file ijms-26-05519-s001.zip › Supplementary Materials/Supplementary Material S3/New image WB-COX-1/actin 2-Original.jpg]

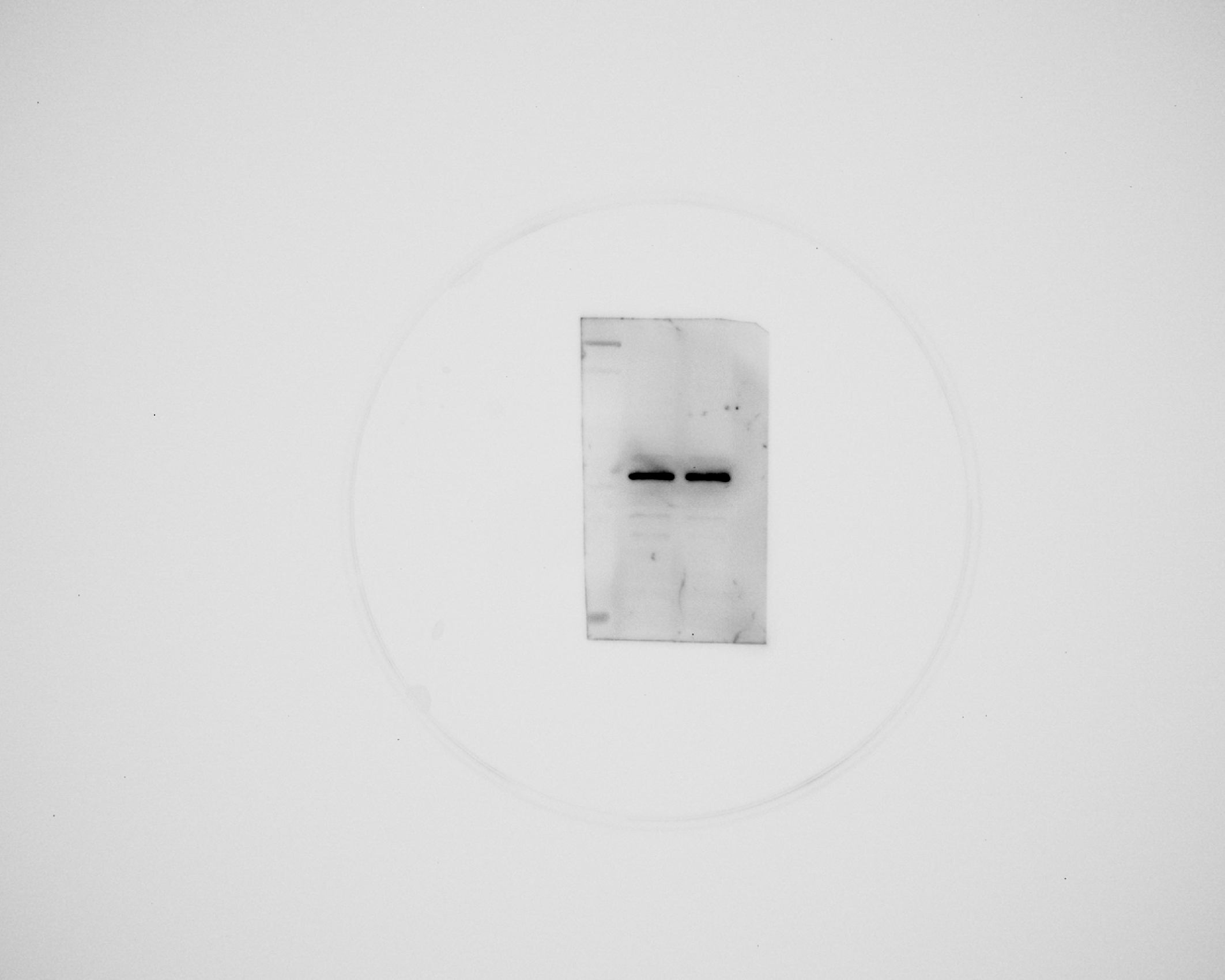

Supplement: Supplementary file 1 [file ijms-26-05519-s001.zip › Supplementary Materials/Supplementary Material S3/New image WB-COX-1/actin 3-Comparison plot.jpg]

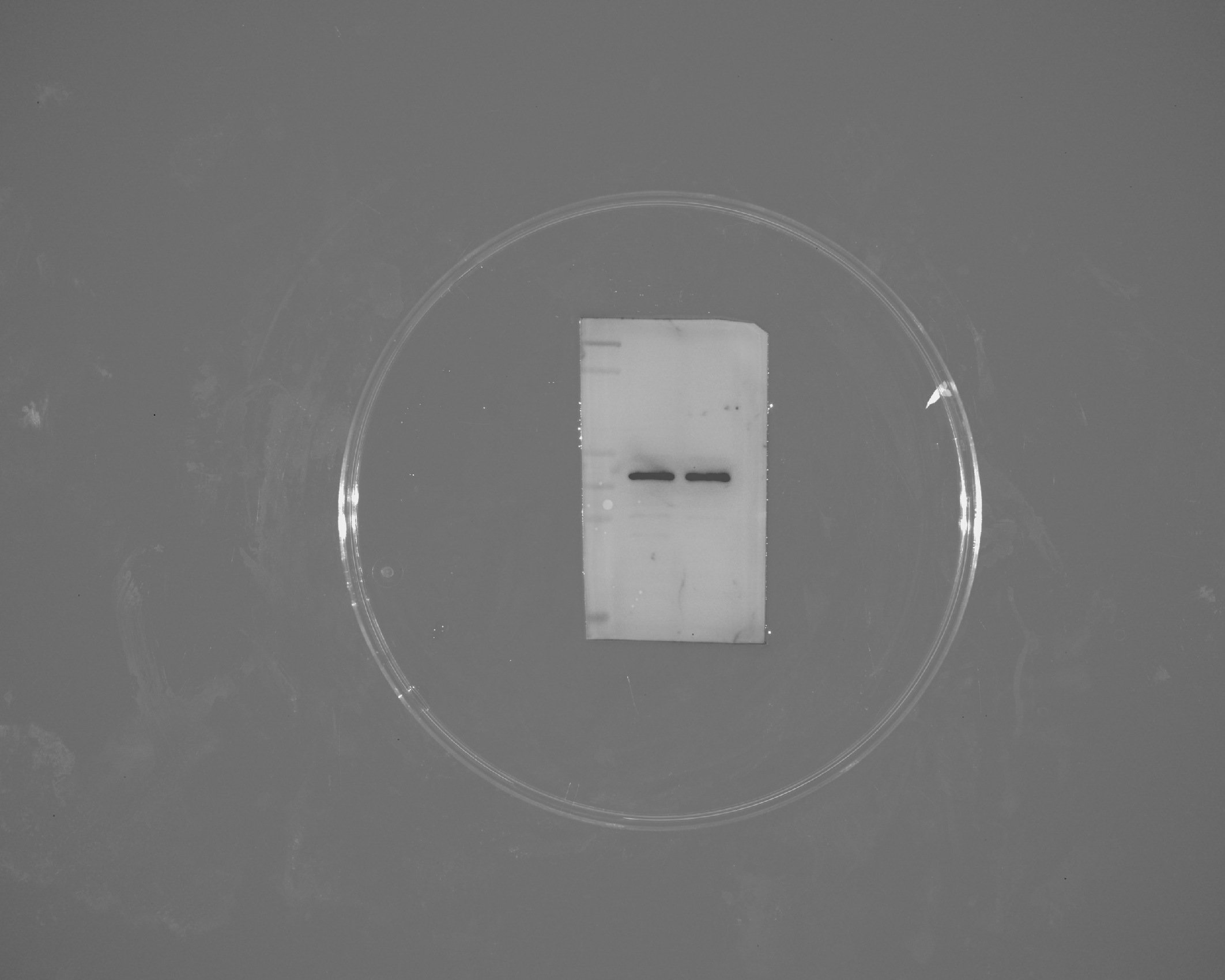

Supplement: Supplementary file 1 [file ijms-26-05519-s001.zip › Supplementary Materials/Supplementary Material S3/New image WB-COX-1/actin 3-Molecular weight marker.jpg]

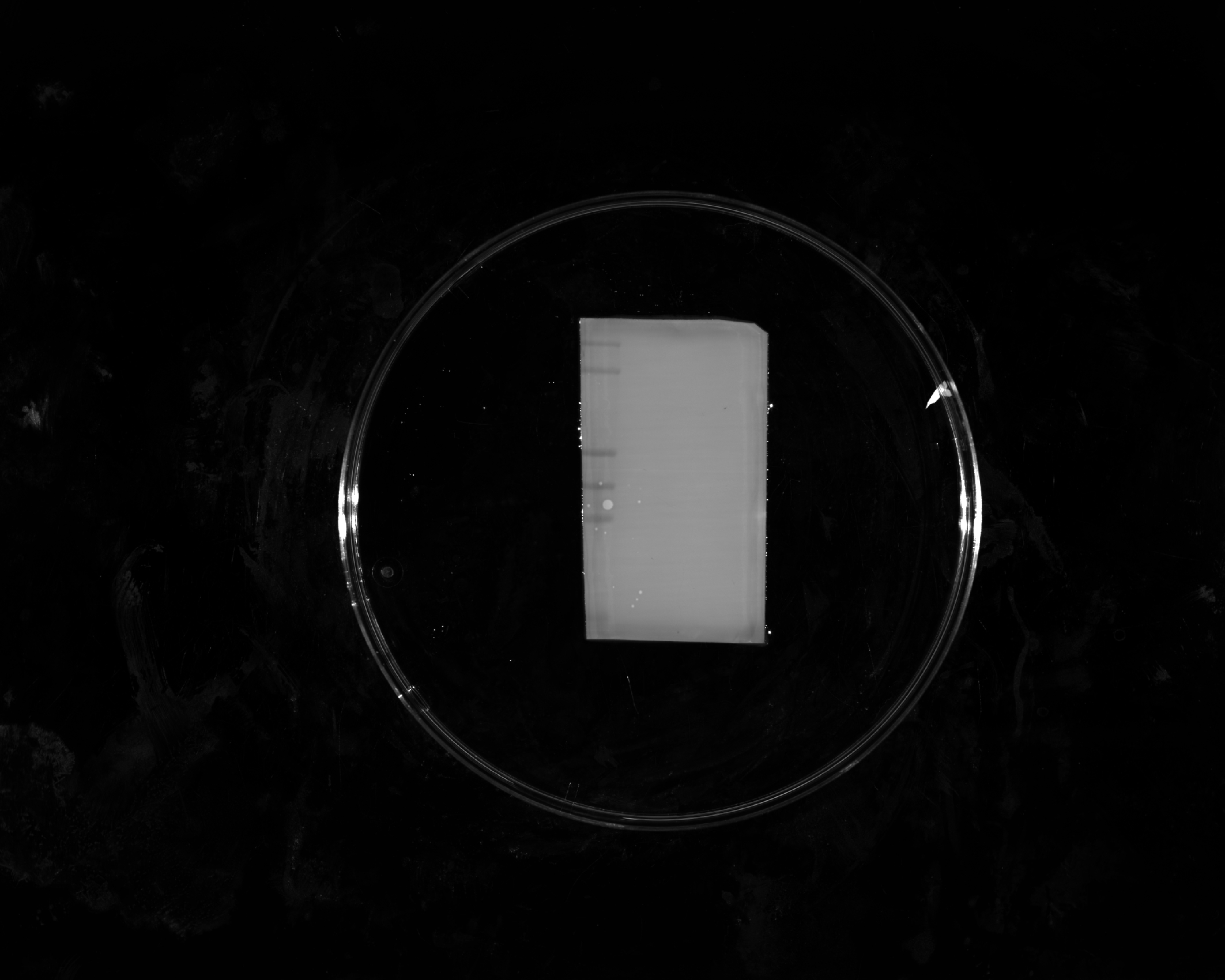

Supplement: Supplementary file 1 [file ijms-26-05519-s001.zip › Supplementary Materials/Supplementary Material S3/New image WB-COX-1/actin 3-Original.jpg]

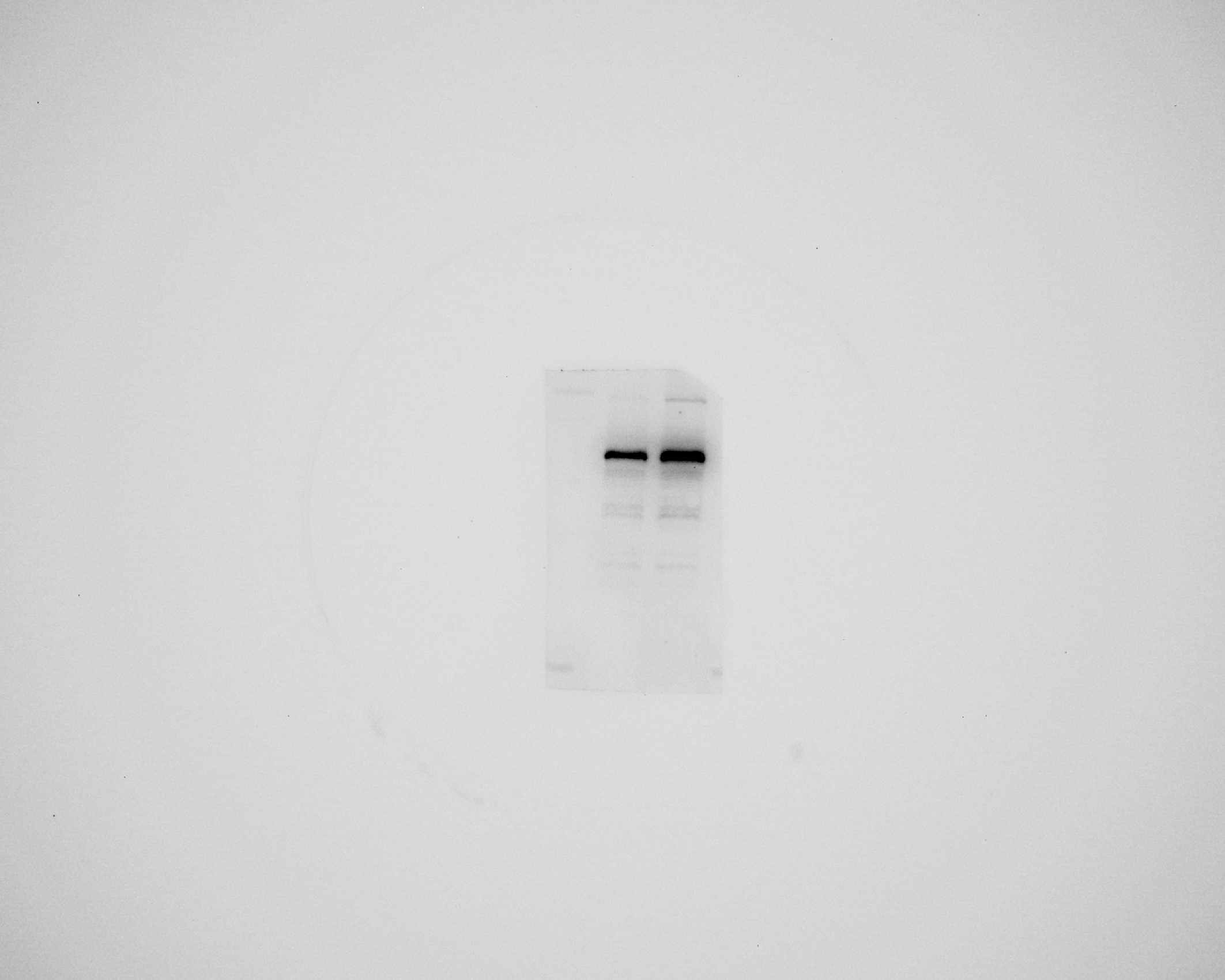

Supplement: Supplementary file 1 [file ijms-26-05519-s001.zip › Supplementary Materials/Supplementary Material S3/New image WB-COX-1/cox1 1-Comparison plot.jpg]

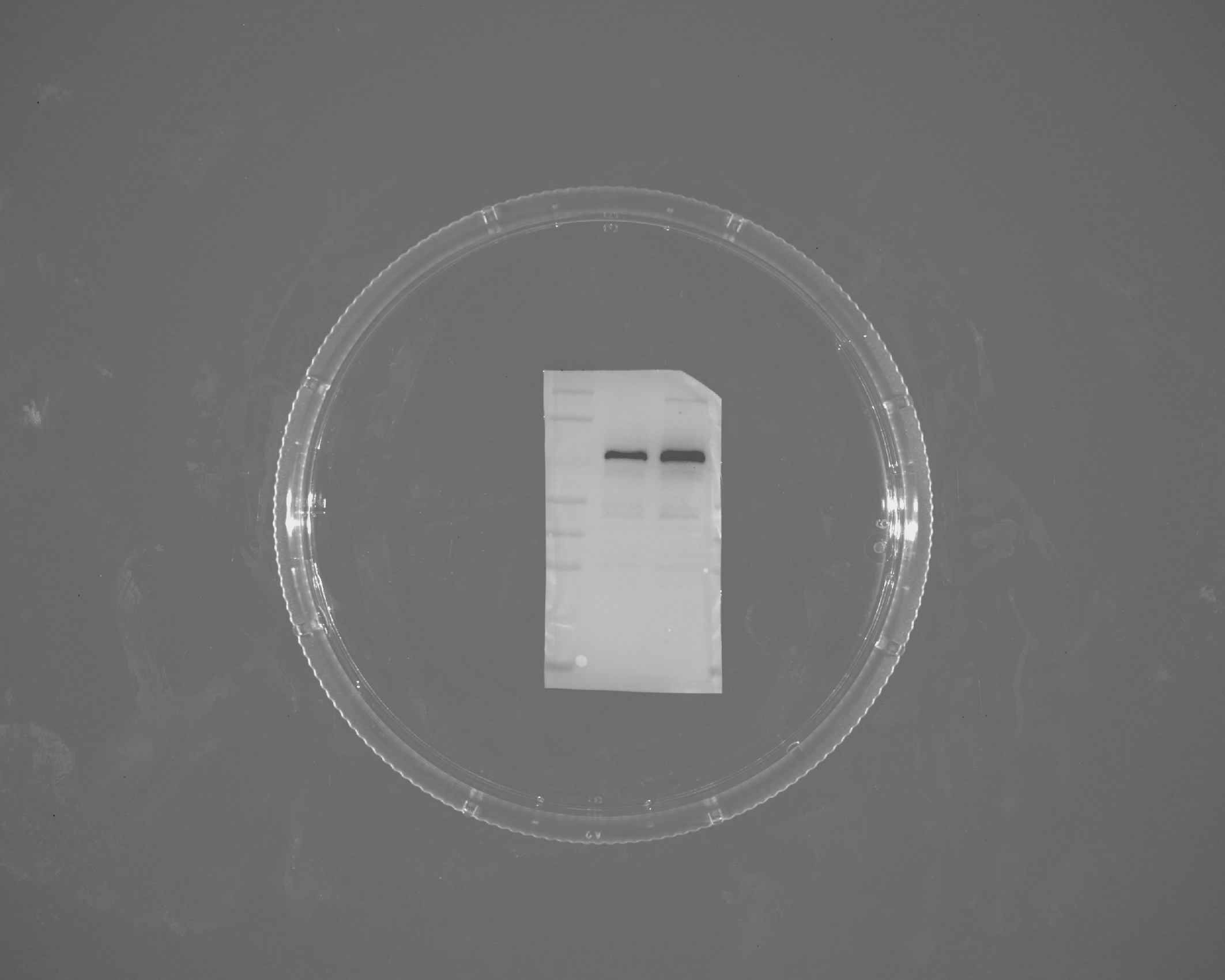

Supplement: Supplementary file 1 [file ijms-26-05519-s001.zip › Supplementary Materials/Supplementary Material S3/New image WB-COX-1/cox1 1-Molecular weight marker.jpg]

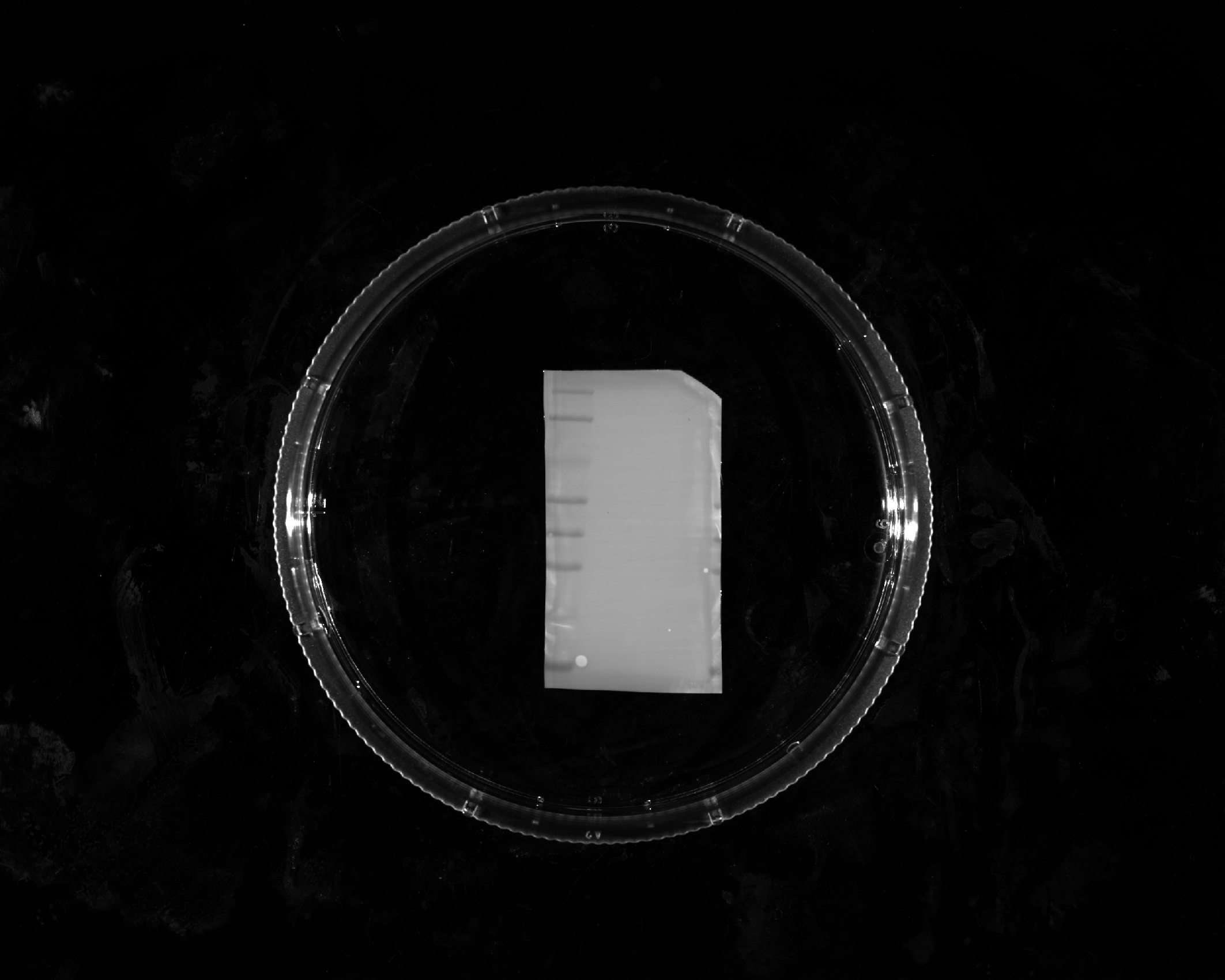

Supplement: Supplementary file 1 [file ijms-26-05519-s001.zip › Supplementary Materials/Supplementary Material S3/New image WB-COX-1/cox1 1-Original.jpg]

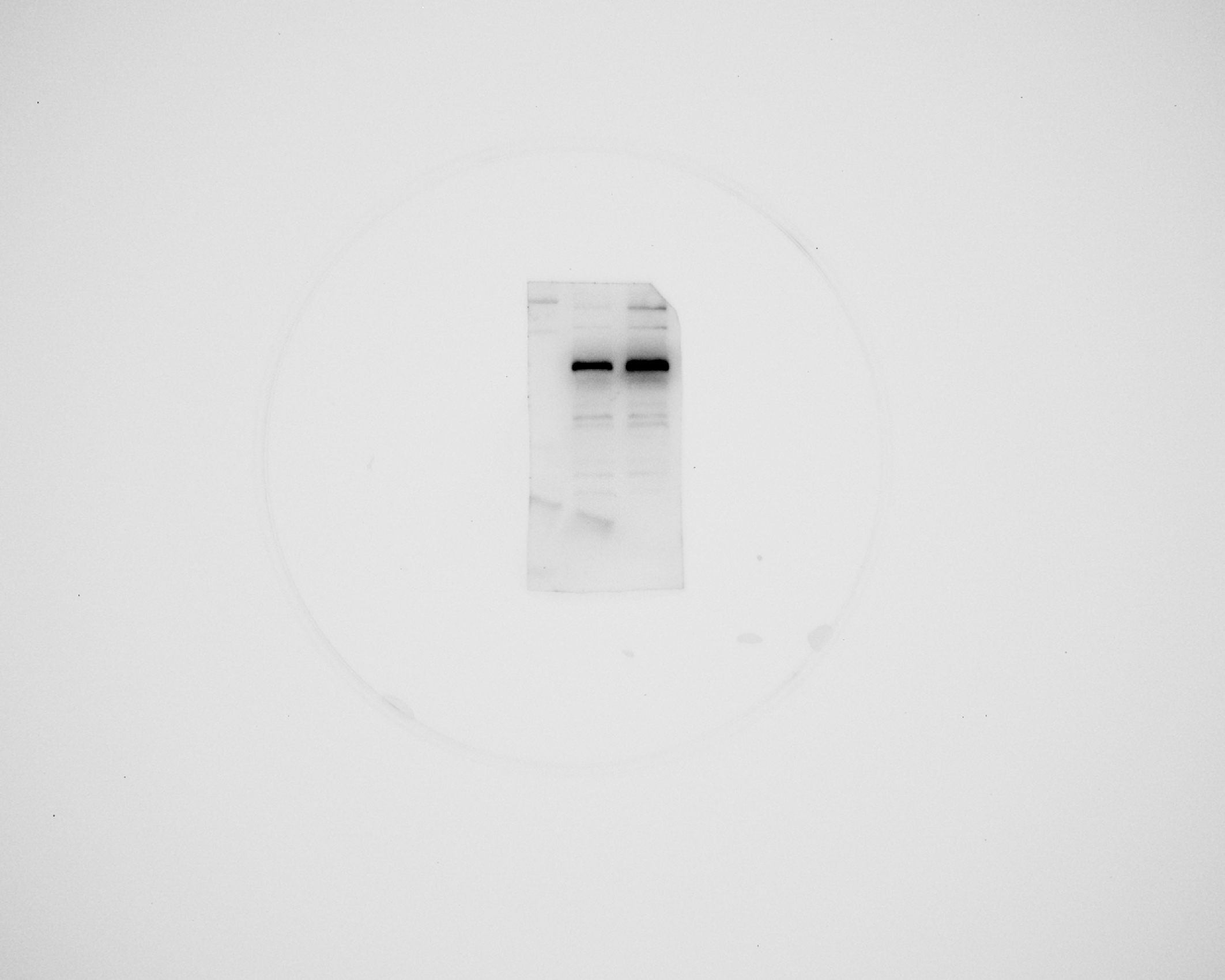

Supplement: Supplementary file 1 [file ijms-26-05519-s001.zip › Supplementary Materials/Supplementary Material S3/New image WB-COX-1/cox1 2-Comparison plot.jpg]

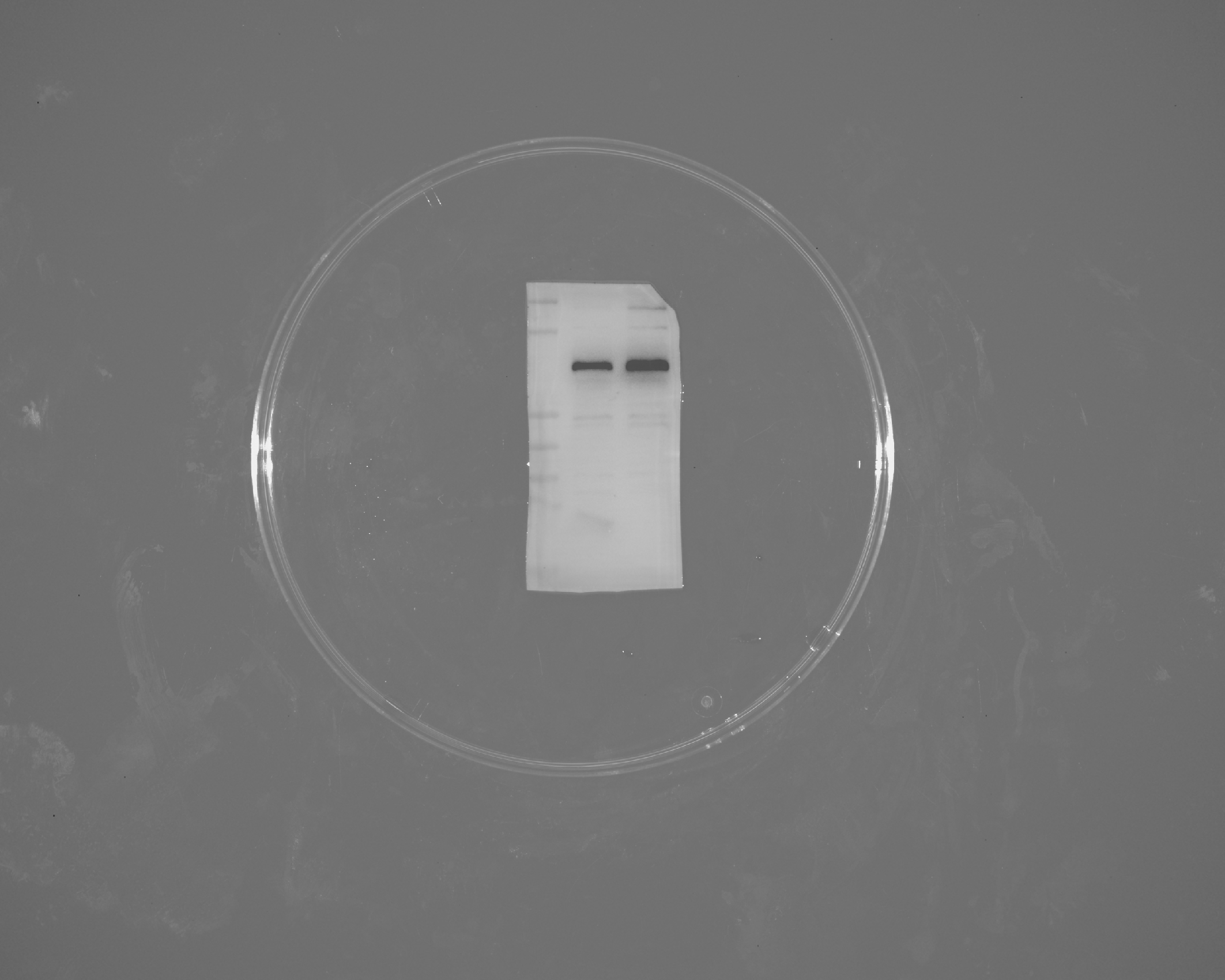

Supplement: Supplementary file 1 [file ijms-26-05519-s001.zip › Supplementary Materials/Supplementary Material S3/New image WB-COX-1/cox1 2-Molecular weight marker.jpg]

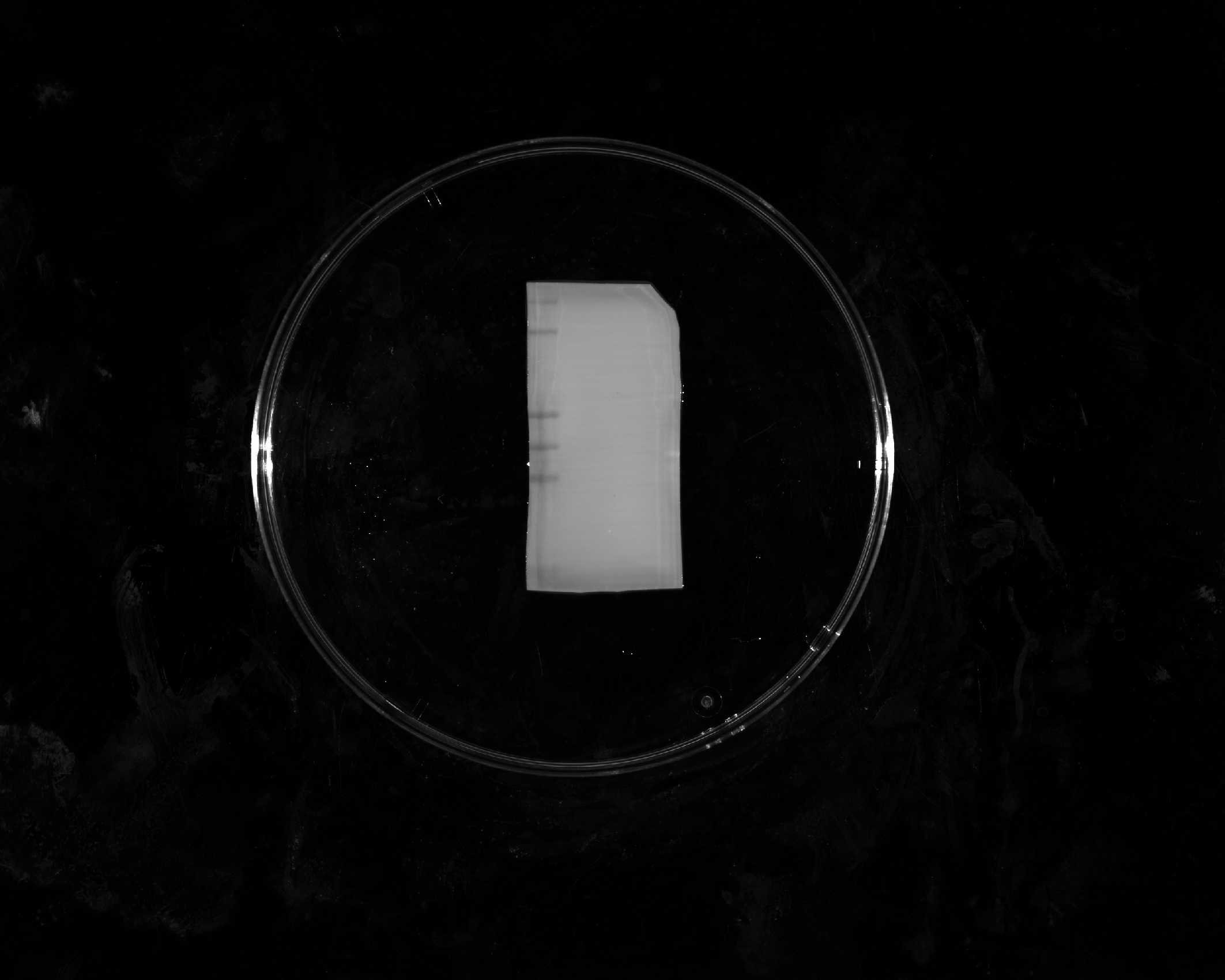

Supplement: Supplementary file 1 [file ijms-26-05519-s001.zip › Supplementary Materials/Supplementary Material S3/New image WB-COX-1/cox1 2-Original.jpg]

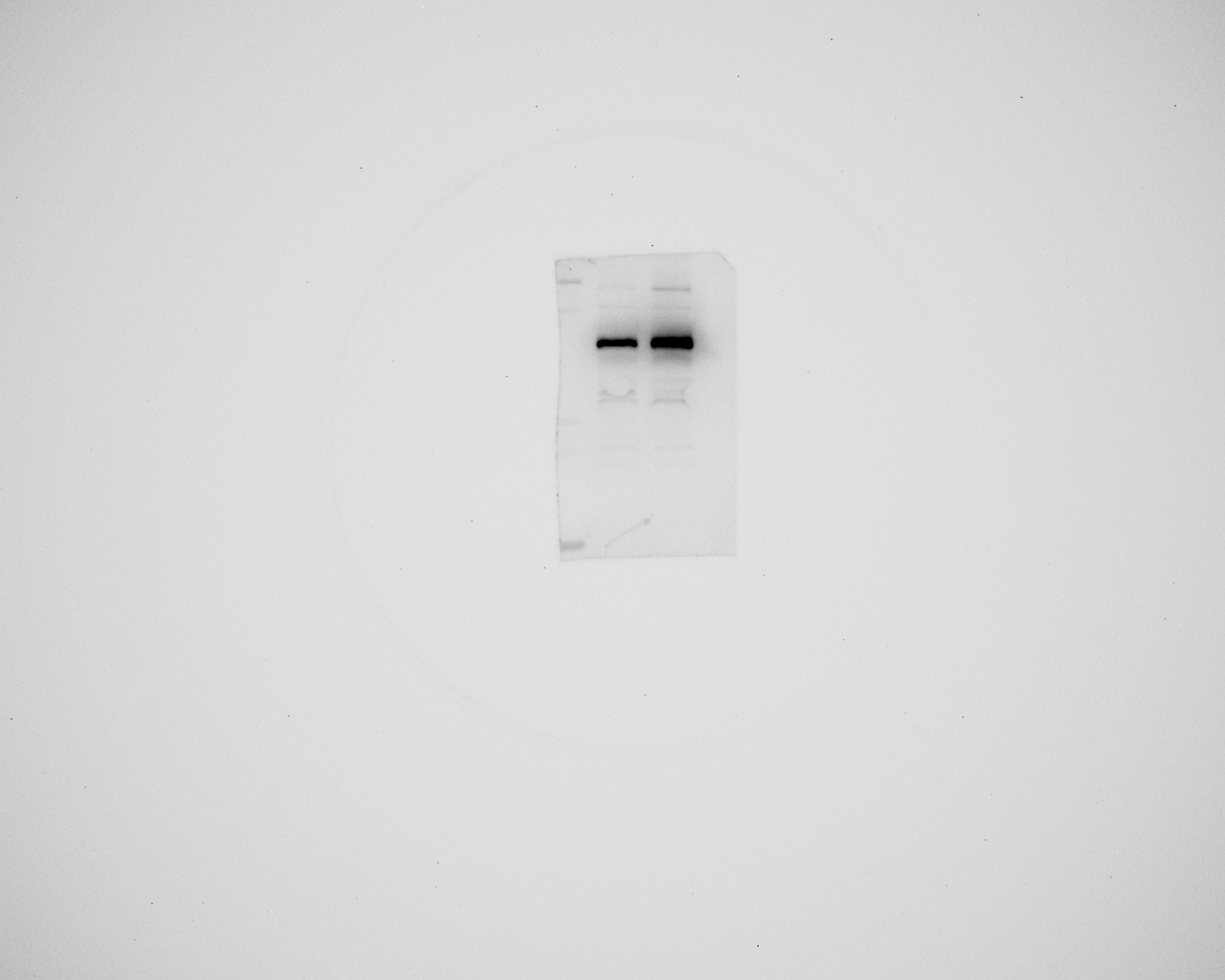

Supplement: Supplementary file 1 [file ijms-26-05519-s001.zip › Supplementary Materials/Supplementary Material S3/New image WB-COX-1/cox1 3-Comparison plot.jpg]

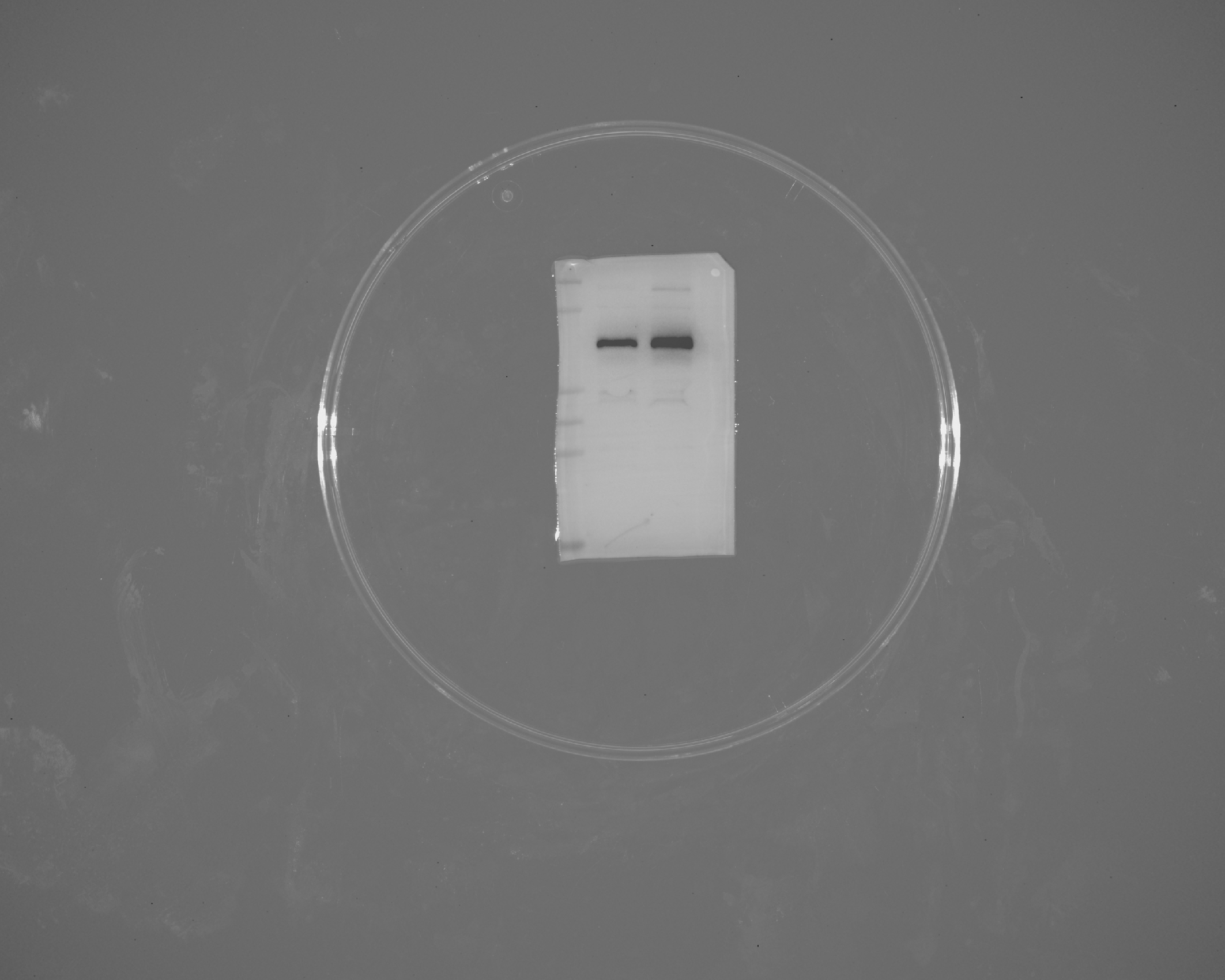

Supplement: Supplementary file 1 [file ijms-26-05519-s001.zip › Supplementary Materials/Supplementary Material S3/New image WB-COX-1/cox1 3-Molecular weight marker.jpg]

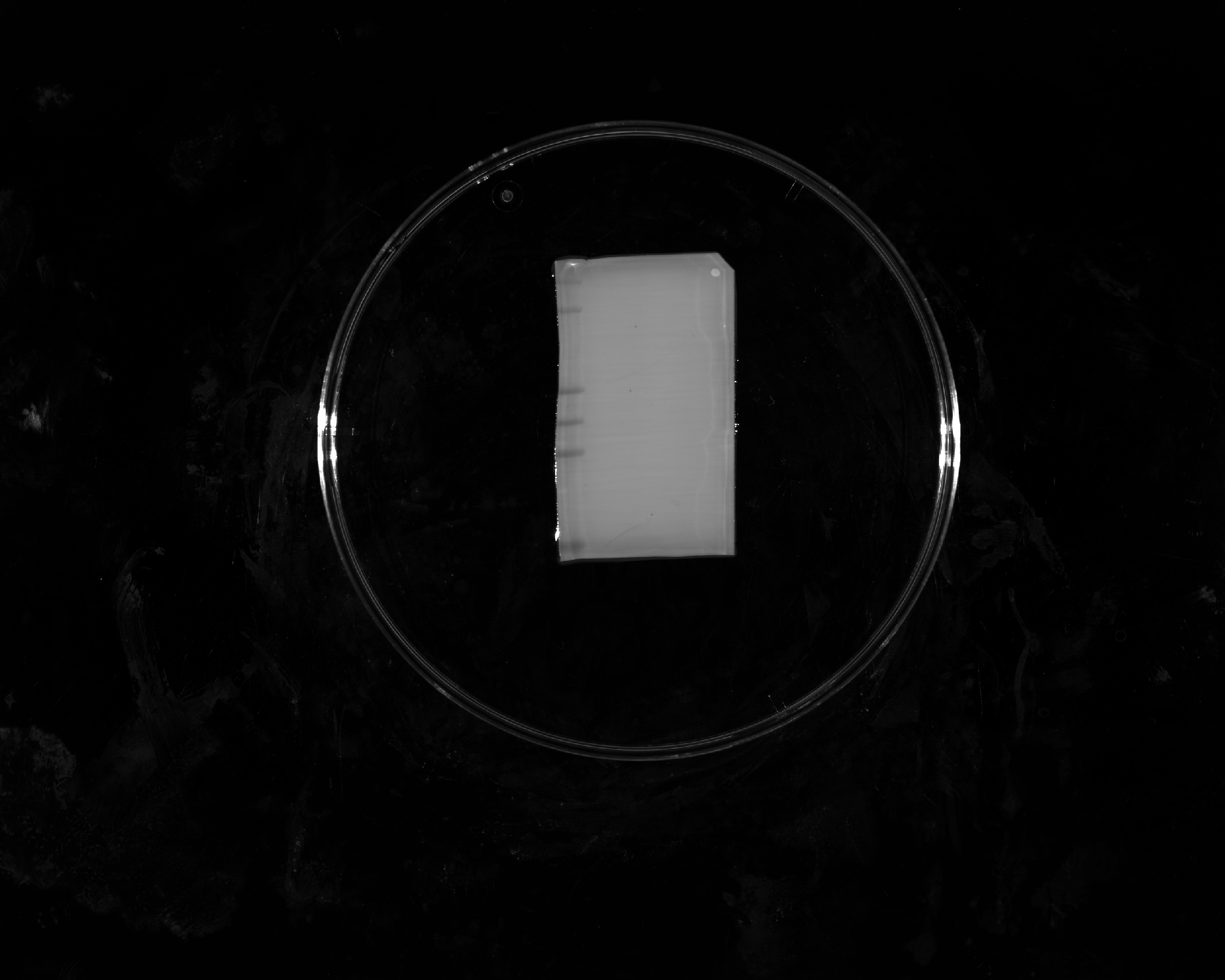

Supplement: Supplementary file 1 [file ijms-26-05519-s001.zip › Supplementary Materials/Supplementary Material S3/New image WB-COX-1/cox1 3-Original.jpg]

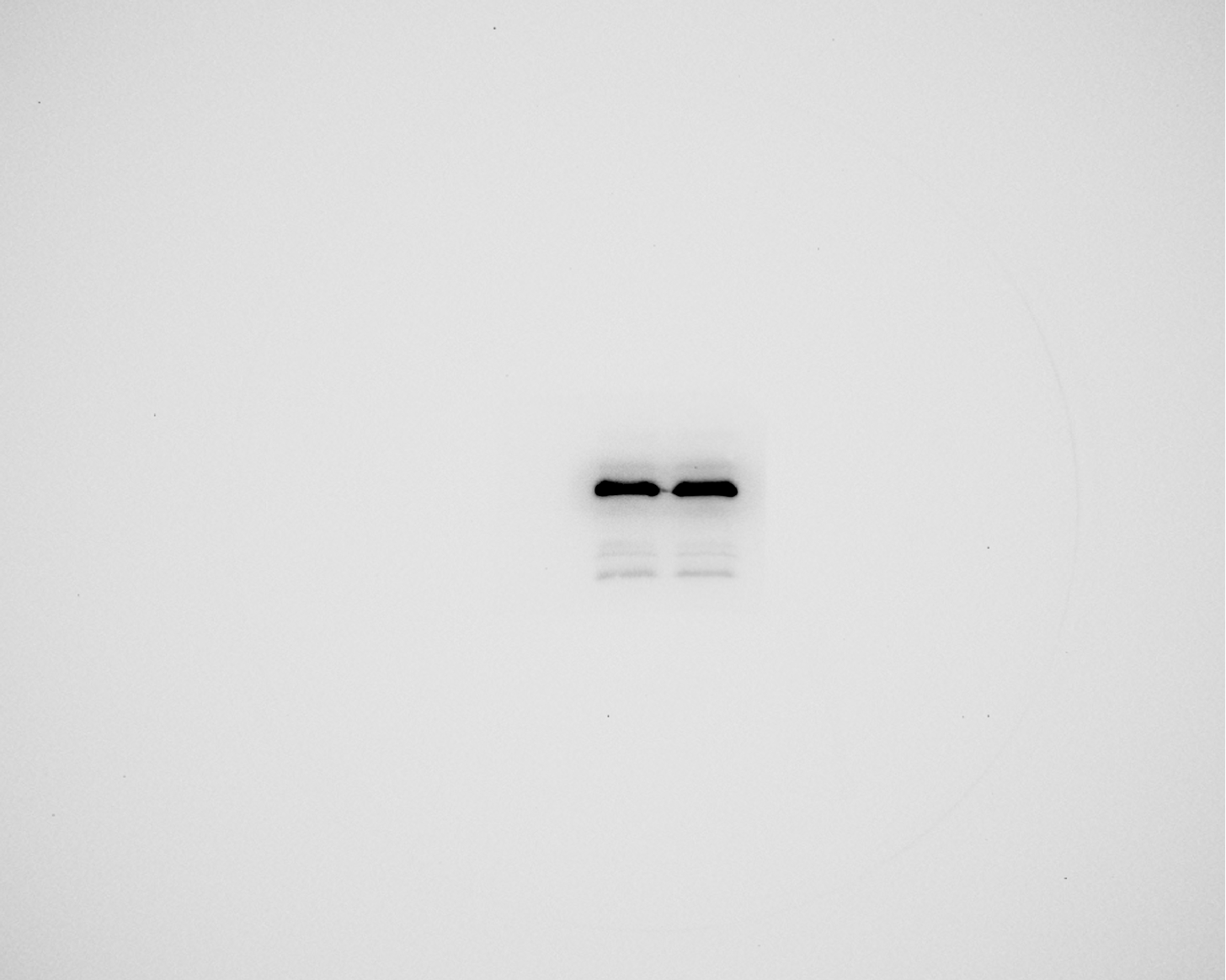

Supplement: Supplementary file 1 [file ijms-26-05519-s001.zip › Supplementary Materials/Supplementary Material S3/Previous image WB-COX-1/Fig6-a-actin -1.jpg]

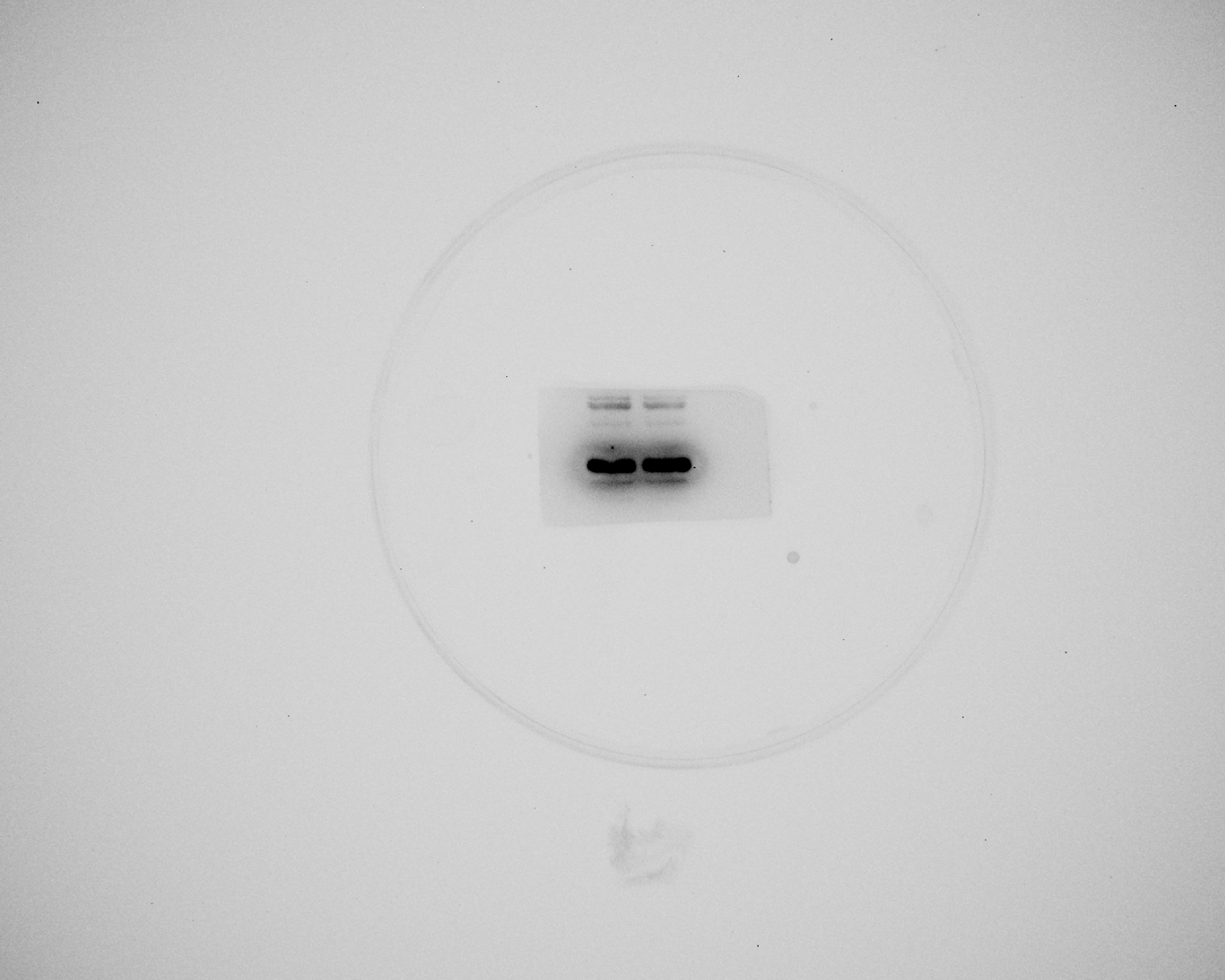

Supplement: Supplementary file 1 [file ijms-26-05519-s001.zip › Supplementary Materials/Supplementary Material S3/Previous image WB-COX-1/Fig6-a-actin -2.jpg]

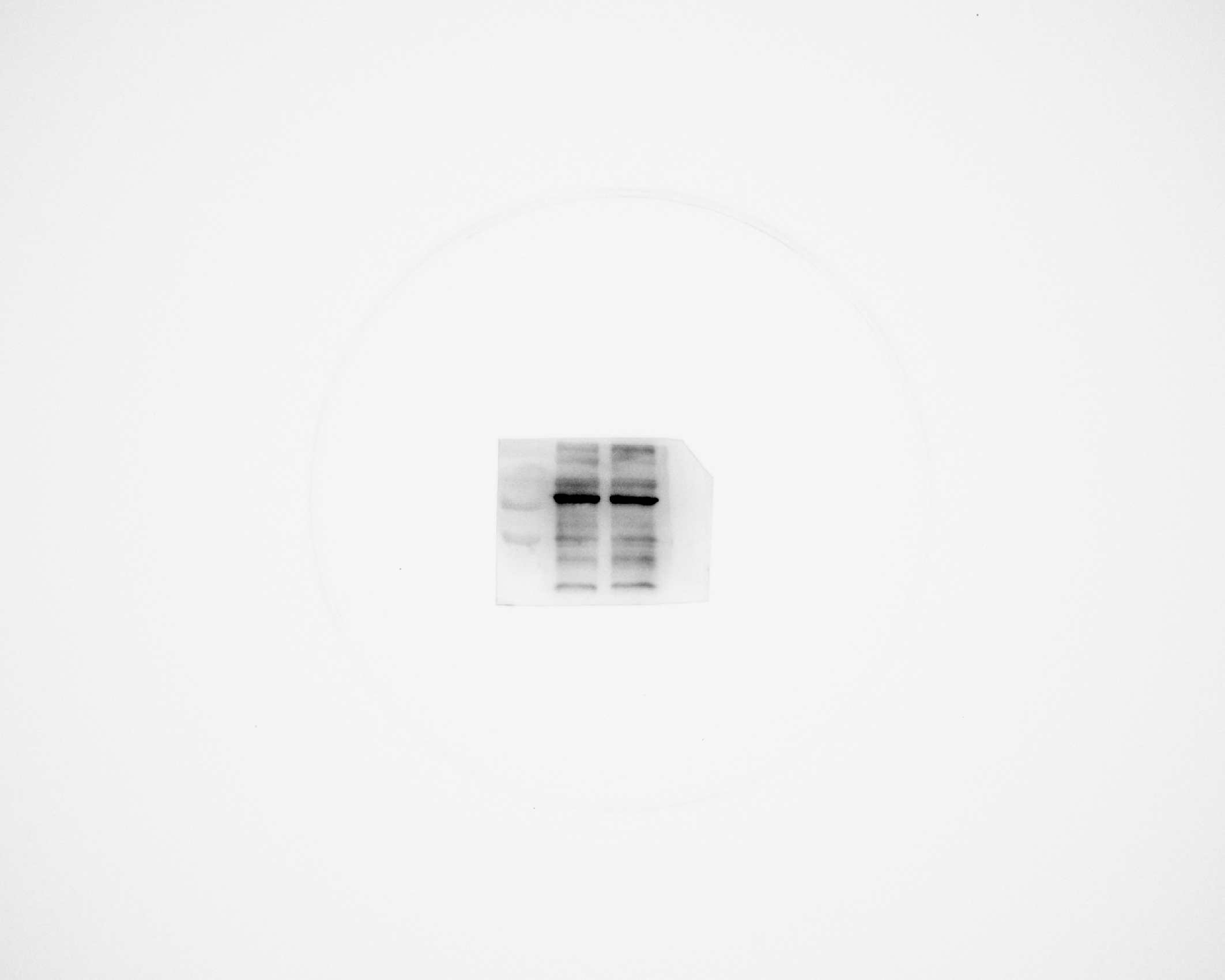

Supplement: Supplementary file 1 [file ijms-26-05519-s001.zip › Supplementary Materials/Supplementary Material S3/Previous image WB-COX-1/Fig6-a-actin -3.jpg]

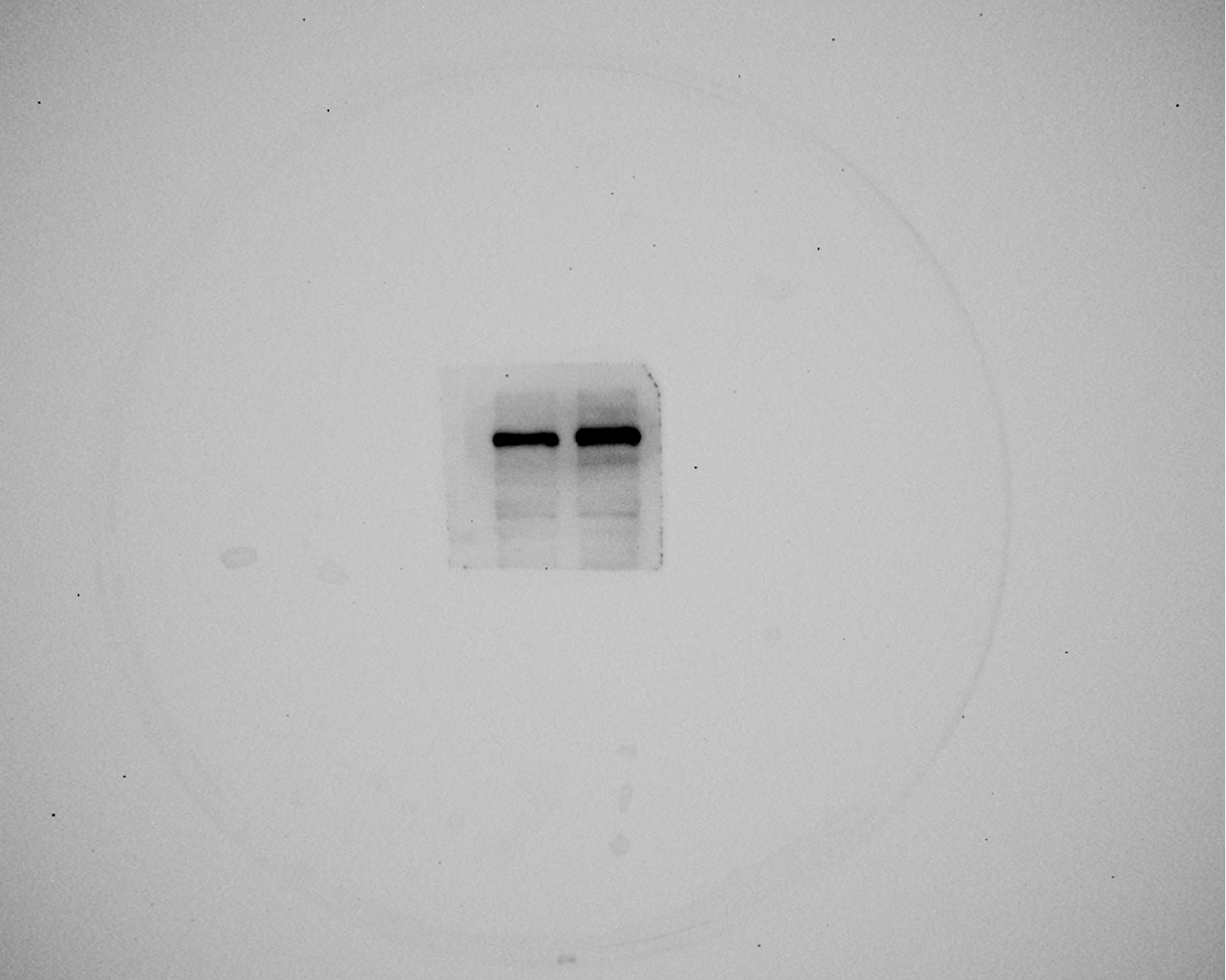

Supplement: Supplementary file 1 [file ijms-26-05519-s001.zip › Supplementary Materials/Supplementary Material S3/Previous image WB-COX-1/Fig6-a-cox1 -1.jpg]

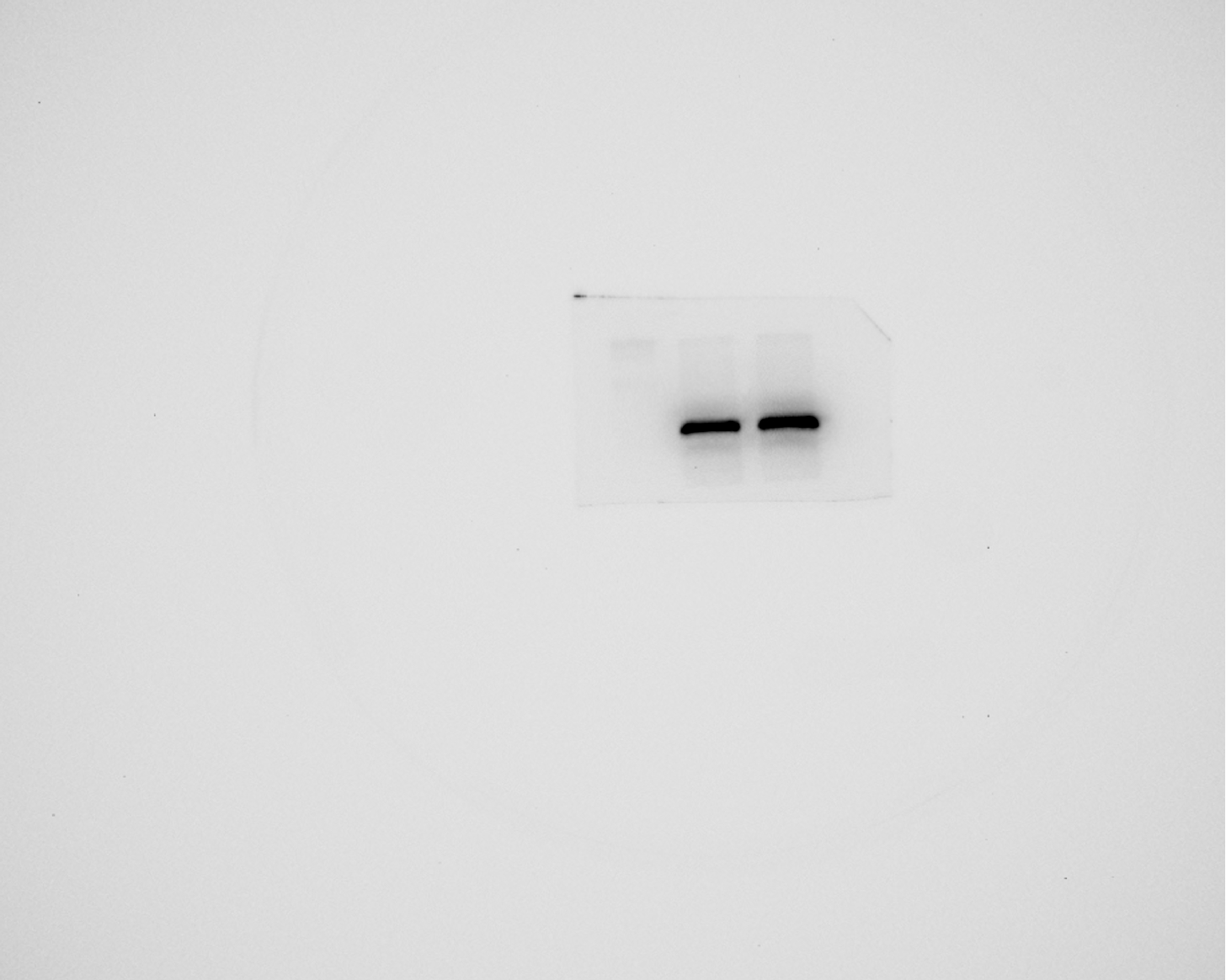

Supplement: Supplementary file 1 [file ijms-26-05519-s001.zip › Supplementary Materials/Supplementary Material S3/Previous image WB-COX-1/Fig6-a-cox1 -2.jpg]

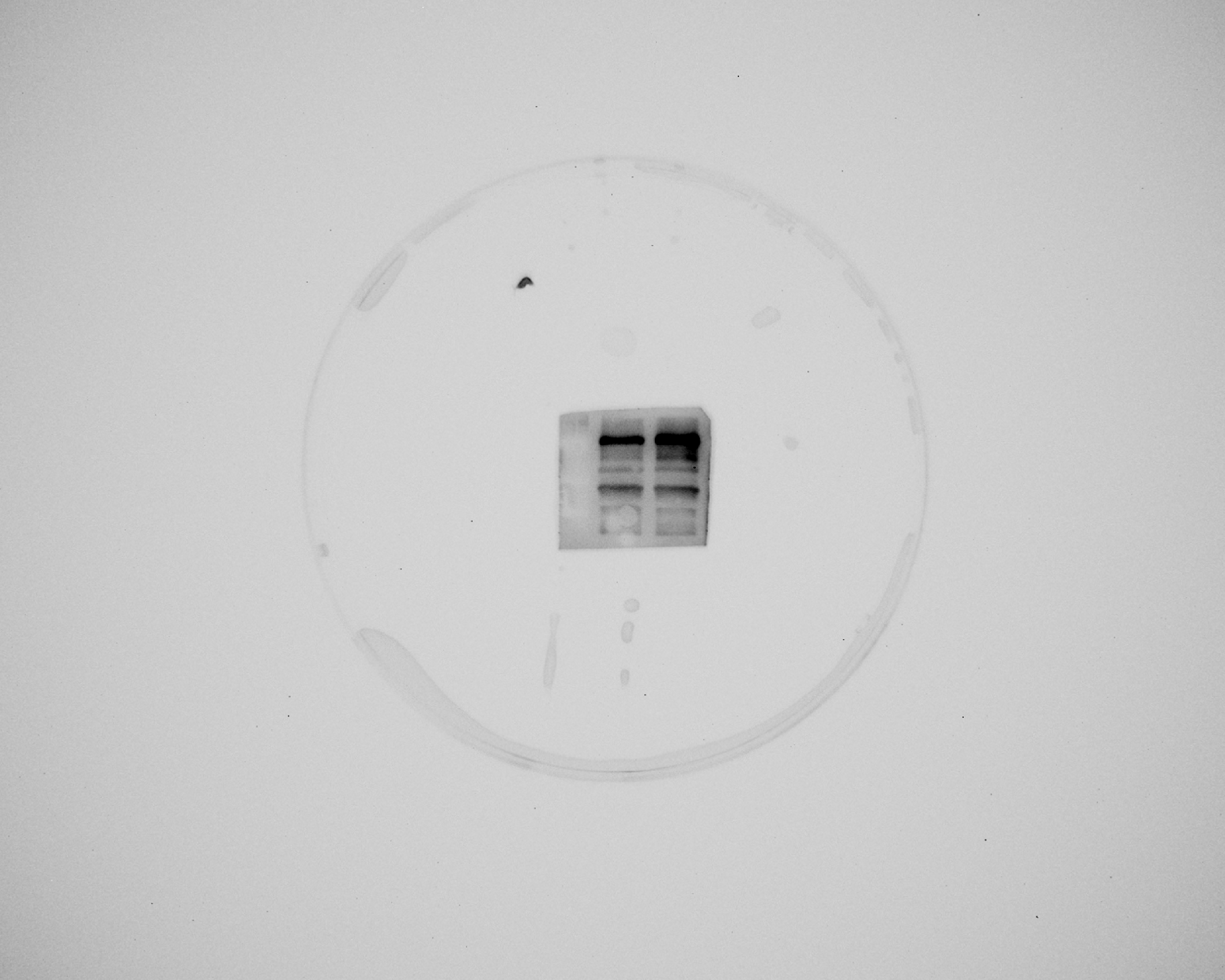

Supplement: Supplementary file 1 [file ijms-26-05519-s001.zip › Supplementary Materials/Supplementary Material S3/Previous image WB-COX-1/Fig6-a-cox1 -3.jpg]

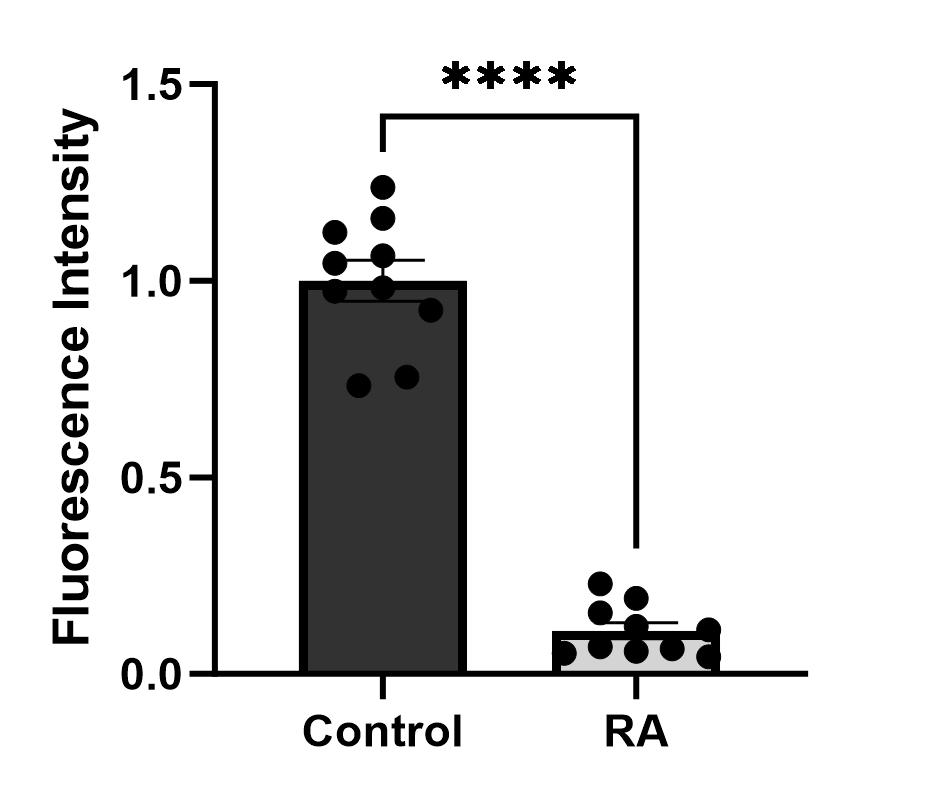

Supplement: Supplementary file 1 [file ijms-26-05519-s001.zip › Supplementary Materials/Supplementary Material S4/COL2A1.jpg]

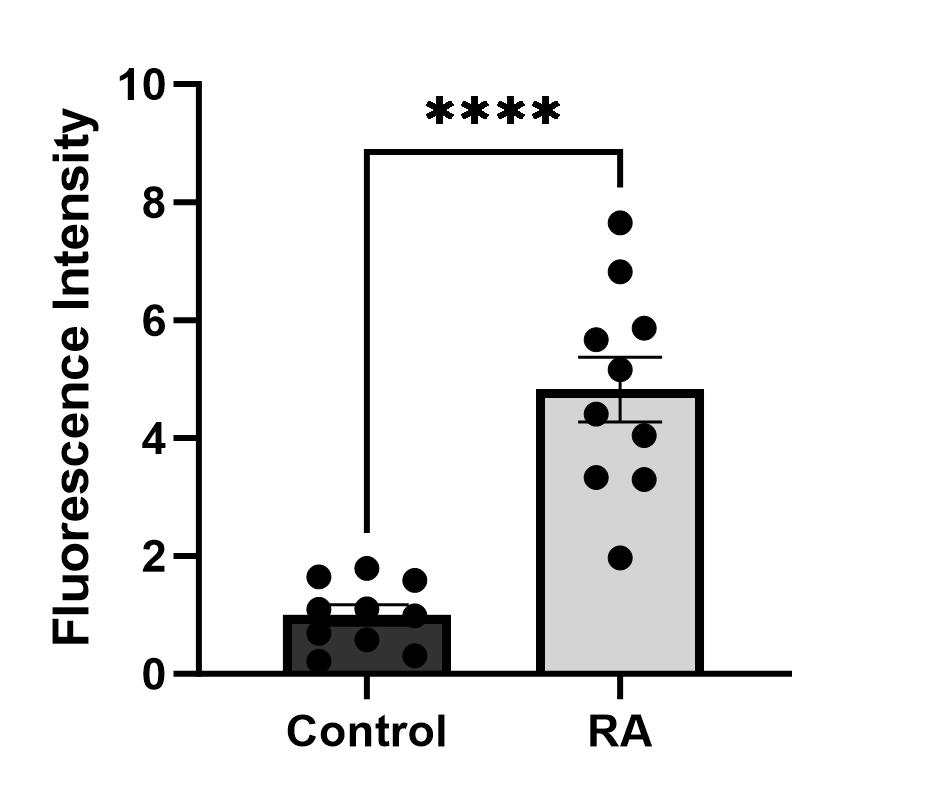

Supplement: Supplementary file 1 [file ijms-26-05519-s001.zip › Supplementary Materials/Supplementary Material S4/MMP13.jpg]
